# Cell-Impermeable Inhibitors Confirm That Intracellular Human Transglutaminase 2 Is Responsible for the Transglutaminase-Associated Cancer Phenotype

**DOI:** 10.3390/ijms241612546

**Published:** 2023-08-08

**Authors:** Eric W. J. Gates, Nicholas D. Calvert, Nicholas J. Cundy, Federica Brugnoli, Pauline Navals, Alexia Kirby, Nicoletta Bianchi, Gautam Adhikary, Adam J. Shuhendler, Richard L. Eckert, Jeffrey W. Keillor

**Affiliations:** 1 Department of Chemistry and Biomolecular Sciences, University of Ottawa, Ottawa, ON K1N 6N5, Canada; egate028@uottawa.ca (E.W.J.G.); ncalvert@uottawa.ca (N.D.C.); n.cundy@hotmail.com (N.J.C.); pnavals@uottawa.ca (P.N.); akirby@uottawa.ca (A.K.); adam.shuhendler@uottawa.ca (A.J.S.); 2 Department of Translational Medicine, University of Ferrara, 44021 Ferrara, Italy; federica.brugnoli@unife.it (F.B.); nicoletta.bianchi@unife.it (N.B.); 3 Department of Biochemistry and Molecular Biology, University of Maryland School of Medicine, Baltimore, MD 21201, USA; gadhikary@som.umaryland.edu (G.A.); reckert@som.umaryland.edu (R.L.E.)

**Keywords:** transglutaminase 2, cancer, fluorescent probe, inhibition, chemical label, cell impermeable, fluorescence microscopy

## Abstract

Transglutaminase 2 (TG2) is a multifunctional enzyme primarily responsible for crosslinking proteins. Ubiquitously expressed in humans, TG2 can act either as a transamidase by crosslinking two substrates through formation of an N^ε^(ɣ-glutaminyl)lysine bond or as an intracellular G-protein. These discrete roles are tightly regulated by both allosteric and environmental stimuli and are associated with dramatic changes in the conformation of the enzyme. The pleiotropic nature of TG2 and multi-faceted activities have resulted in TG2 being implicated in numerous disease pathologies including celiac disease, fibrosis, and cancer. Targeted TG2 therapies have not been selective for subcellular localization, such that currently no tools exist to selectively target extracellular over intracellular TG2. Herein, we have designed novel TG2-selective inhibitors that are not only highly potent and irreversible, but also cell impermeable, targeting only extracellular TG2. We have also further derivatized the scaffold to develop probes that are intrinsically fluorescent or bear an alkyne handle, which target both intra- and extracellular TG2, in order to facilitate cellular labelling and pull-down assays. The fluorescent probes were internalized and imaged in cellulo, and provide the first implicit experimental evidence that by comparison with their cell-impermeable analogues, it is specifically *intracellular* TG2, and presumably its G-protein activity, that contributes to transglutaminase-associated cancer progression.

## 1. Introduction

The transglutaminases (TGases) are a family of multi-functional enzymes primarily responsible for cross-linking proteins [1,2,3]. The family is composed of eight calcium-dependent isozymes that can be found throughout the human body. These enzymes mediate the formation of a covalent link between two substrate proteins through the formation of an N^ε^(ɣ-glutaminyl)lysine bond, through a transamidation reaction involving a catalytic cysteine residue [4]. Transglutaminase 2 (TG2), or tissue transglutaminase, is of specific interest not only for its ubiquitous expression and cross-linking activity, but also for its ability to act as a G-protein [5]. TG2 can adopt two dramatically different conformations that are exclusively associated with its two distinct activities [6]. When acting as a G-protein, the four sub-domains of TG2 are folded in on themselves in a compact ‘closed’ conformation, obscuring the cross-linking active site and forming a GTP binding site on the medial *C*-terminal β-barrel [7]. However, when TG2 adopts its cross-linking enzymatic form, the two *C*-terminal β-barrels are extended away from the catalytic core in an ‘open’ linear conformation, exposing substrate protein binding sites, while dismantling the GTP binding site [8]. Present in both extracellular and intracellular locations [9,10,11,12,13], TG2 has been implicated in numerous pathologies ranging from the survival and epithelial-mesenchymal transition of cancer stem cells to fibrosis to celiac disease [14,15,16,17,18,19,20,21]. Targeted TG2 therapies in the treatment of celiac disease and liver fibrosis have recently been progressed to Phase 2b clinical trials [22,23]; however, there remains a need to further investigate the roles of TG2 in other biological contexts. One of the areas that remains to be thoroughly investigated is the effect of targeting extracellular versus intracellular TG2. Given that the protein is present both inside and outside the cell, extracellular-selective inhibition may allow the unambiguous assignment of various roles of extracellular TG2, without convoluting this interpretation through the simultaneous inhibition of intracellular TG2. The lack of precise chemical tools has also hindered investigation into which TG2 activities are associated with which sub-cellular environments. The role of TG2 in the proliferation and migration of cancer cells has been ascribed to both intracellular (G-protein) [14,24,25,26,27,28] and extracellular (crosslinking) [29,30,31] activities, suggesting that the specific localization of the enzyme associated with a phenotype (and cell type) has yet to be assigned. This ambiguity can be viewed as incomplete target validation that hinders the development of potential therapeutic agents.

Herein, we disclose several novel chemical probes built on a peptidomimetic scaffold designed to be selective for TG2 over other isozymes. The probes include functional groups that decrease cell permeability, are fluorescent, or provide sites for bioorthogonal reactivity. Herein, we show that the first-in-class cell-impermeable irreversible inhibitors are highly potent but fail to alter cancer stem cell progression and invasion. In contrast, the fluorescent version of the probe is both cell permeable and halts cancer cell invasion, providing direct evidence that inhibition of intracellular TG2 (and presumably its G-protein activity) is necessary to generate the anti-cancer phenotype.

## 2. Results and Discussion

### 2.1. Design

**Figure 1:**
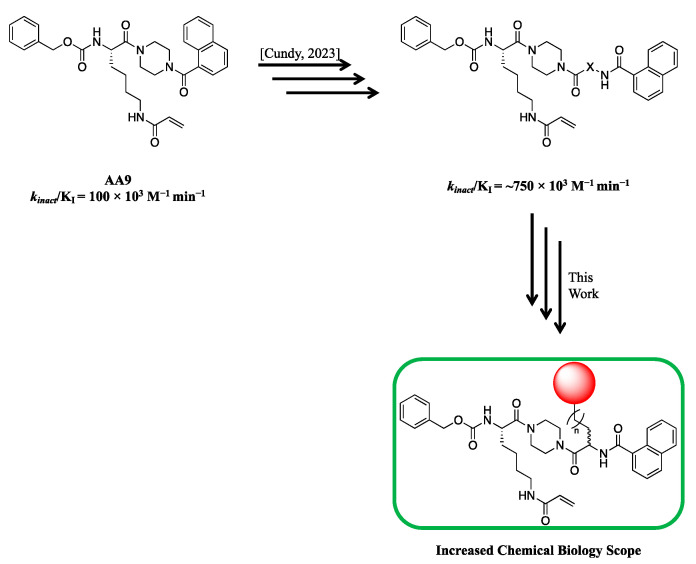
Previous libraries of inhibitors giving rise to the scaffold disclosed in this work [32].

Our previous study of structure–activity relationships in irreversible inhibitors of TG2 revealed a dramatic increase in efficiency when the original lead compound (AA9) [26] was modified to incorporate an additional amino acid spacer residue in its peptidomimetic backbone (Figure 1) [32]. This extension of the peptidomimetic scaffold led to inhibitors that were selective for TG2 over the other isozymes in the TGase family. They were also shown to abolish GTP binding, presumably by locking TG2 in its open conformation [33,34,35,36]. To expressly design first-in-class cell-impermeable inhibitors of TG2, we introduced a bulky negatively charged moiety on the amino acid spacer of the scaffold. This was achieved through solid-phase peptide synthesis (SPPS) of a tri-Asp sequence, connected to the rest of the scaffold through an alkyl or peptidic linker. The extended scaffold was also modified at the same site by incorporating bright fluorophores, producing novel TG2 fluorescent probes. Finally, the same site was modified to add an alkyne group, to allow subsequent copper-assisted azide alkyne cycloaddition (CuAAC) reactions for attaching alternative cargo.

### 2.2. Synthesis

**Scheme 1:**
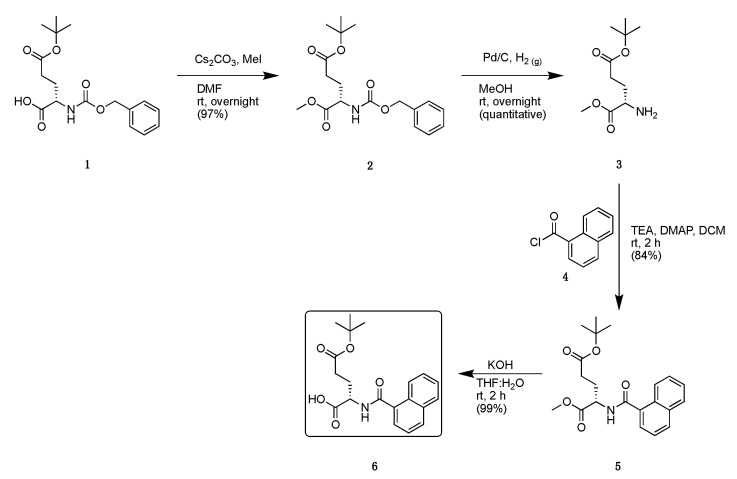
Synthetic scheme to generate naphthoyl-glutamate moiety.

To generate these various chemical tools, a linear synthetic scheme was designed, featuring the formation of the naphthoyl-glutamate moiety **6** (Scheme 1) and a common stable key intermediate **16**. Derivatization of the glutamate residue in the peptidomimetic scaffold of the key intermediate **16** then allowed various chemical tools to be obtained by late-stage diversification.

**Scheme 2:**
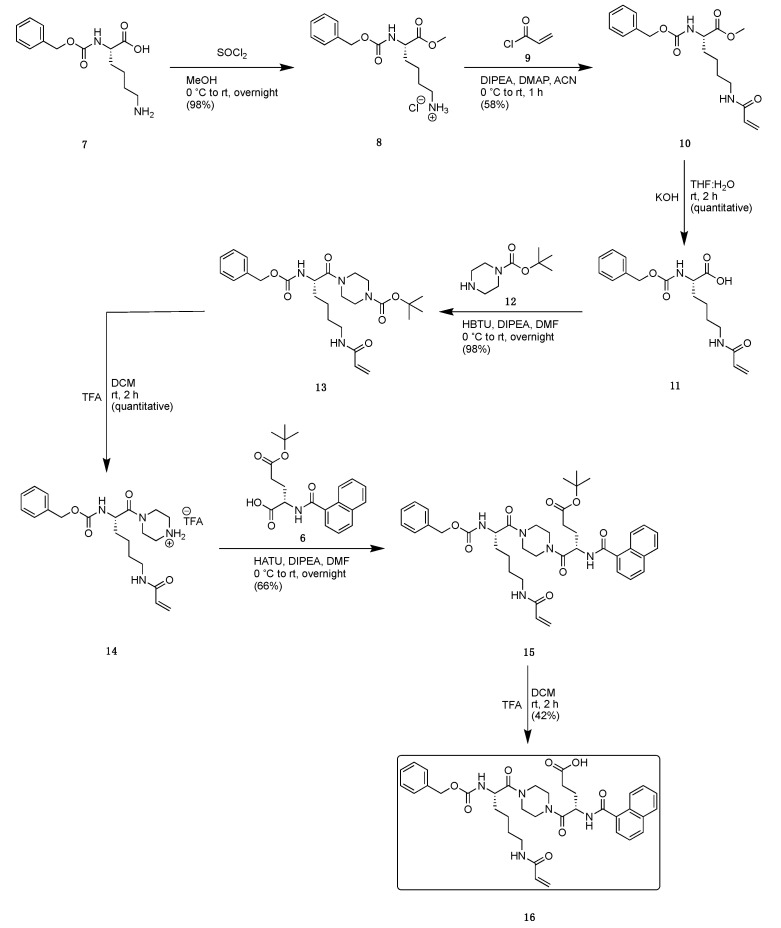
Synthetic scheme to generate key intermediate, allowing for subsequent functionalization of the glutamate sidechain.

Starting from commercially available Z-Glu(OtBu)-OH (**1**), a methylation was performed to generate the corresponding methyl ester **2** (Scheme 1). The Cbz protecting group was then removed by palladium catalyzed hydrogenolysis to liberate the *N*-terminus on glutamate **3**. Acylation with 1-naphthoyl chloride **4** was then performed under basic conditions with triethylamine to generate the protected intermediate **5**. Finally, hydrolysis of the *C*-terminal ester generated the naphthoyl-glutamate intermediate **6** to be used in the convergent synthesis described below (Scheme 2).

Commercially available Z-Lys-OH (**7**) was subjected to methyl esterification using thionyl chloride and methanol to protect the *C*-terminus and generate **8** (Scheme 2). The addition of the acrylamide warhead was achieved by reaction with acryloyl chloride **9** and Hünig’s base to yield the acrylated lysine **10**. The *C*-terminal ester of **10** was then hydrolyzed to produce **11**, which was subsequently coupled to *N*-Boc-piperazine **12**, using HATU, to gain access to the Boc-protected amine **13**. Removal of the Boc group with TFA delivered intermediate amine **14** as its TFA salt. Coupling **14** with the glutamate intermediate **6** produced the *t*-butyl ester **15**. A final hydrolysis of this ester using TFA exposed the ɣ-carboxylate of the glutamate and provided key intermediate **16**.

**Scheme 3:**
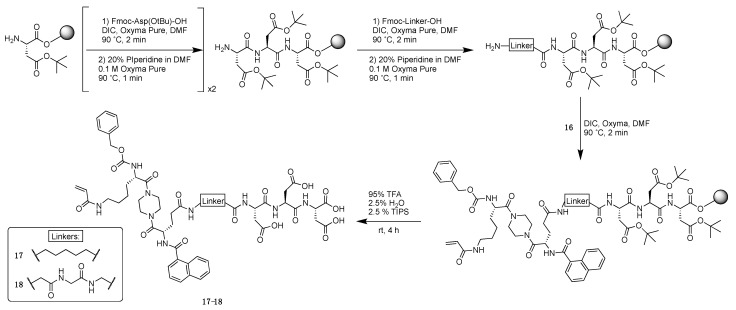
Synthetic scheme from key intermediate to generate cell-impermeable inhibitors **17** and **18**.

Once key intermediate **16** was acquired, cell-impermeable inhibitors were generated by SPPS, allowing all inhibitors to be prepared on-resin in an automated peptide synthesizer (Scheme 3). Starting from manually pre-loaded Fmoc-Asp(OtBu)-Wang resin, two sequential cycles of coupling with Fmoc-Asp and Fmoc deprotection were performed. The resulting tri-Asp peptide was then further derivatized to either incorporate an aminohexanoic acid linker or a tri-Gly linker by further cycles of SPPS. A final coupling with key intermediate **16** executed by the peptide synthesizer produced the protected precursors bound to Wang resin. The resin was subsequently cleaved, and the remaining protecting groups were removed using TFA to yield the cell-impermeable inhibitors **17** and **18**, which were purified by semi-preparative reverse phase HPLC.

**Scheme 4:**
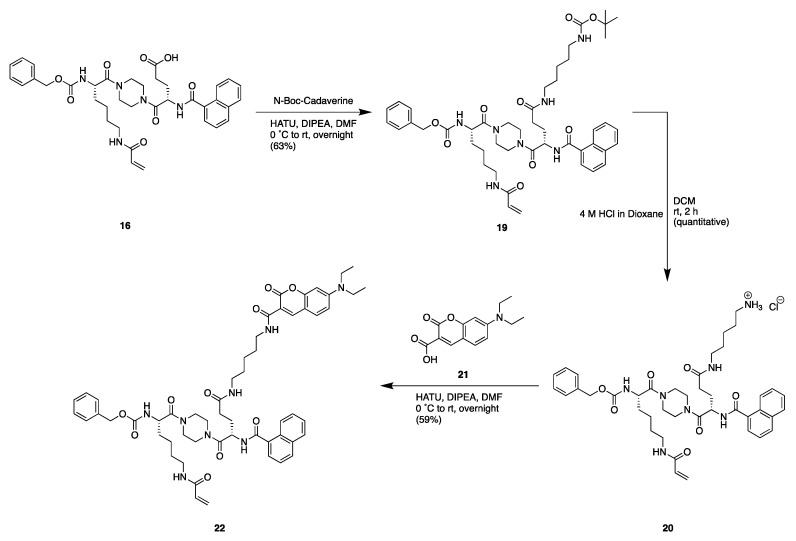
Synthetic scheme from key intermediate to generate fluorescent coumarin probe **22**.

To make fluorescent probes starting from key intermediate **16**, fluorescein, coumarin, and rhodamine B fluorophores were linked to the scaffold through a cadaverine spacer (Scheme 4). Compound **16** was subjected to amide coupling with HATU to attach *N*-Boc-Cadaverine to the glutamate residue. The intermediate amine **19** was then deprotected using 4 M HCl in dioxane and free amine **20** was coupled to the diethylamino coumarin probe **21**. This yielded the coumarin fluorescent probe compound **22** in 59% yield. The addition of FITC to cadaverine intermediate **20** generated fluorescein probe **23** (Scheme S1 in Supplementary Materials).

The preparation of a rhodamine B probe required a different synthetic route relative to the other probes. Starting from commercially available Z-Pro-OH **24**, an amide coupling using HATU was preformed to attach *N*-Boc-cadaverine and generate **25** (Scheme S2 in Supplementary Materials). Palladium-catalyzed hydrogenolysis then deprotected the *N*-terminus of the proline and yielded intermediate **26**. Rhodamine B was then linked to the proline using HATU-mediated amide coupling, providing tertiary amide **27**. The Boc protecting group on the cadaverine linker was then removed using 4 M HCl in dioxane to generate amine **28**. A final amide coupling with HATU and key intermediate **16** provided the rhodamine B probe **29**.

Finally, to generate a ‘clickable’ TG2 labelling agent, the key intermediate **16** was coupled to propargylamine **30** by HATU to generate the propargyl derivative **31** presenting a free alkyne handle (Scheme S3 in Supplementary Materials). To validate that CuAAC was possible on the propargyl scaffold, **31** was exposed to desthiobiotin-PEG3-azide **32**, copper (II) sulfate, and sodium ascorbate (Scheme S4 in Supplementary Materials). The desthiobiotin handle was efficiently installed to give probe **33** in 90% yield.

### 2.3. Kinetics

**Figure 2:**
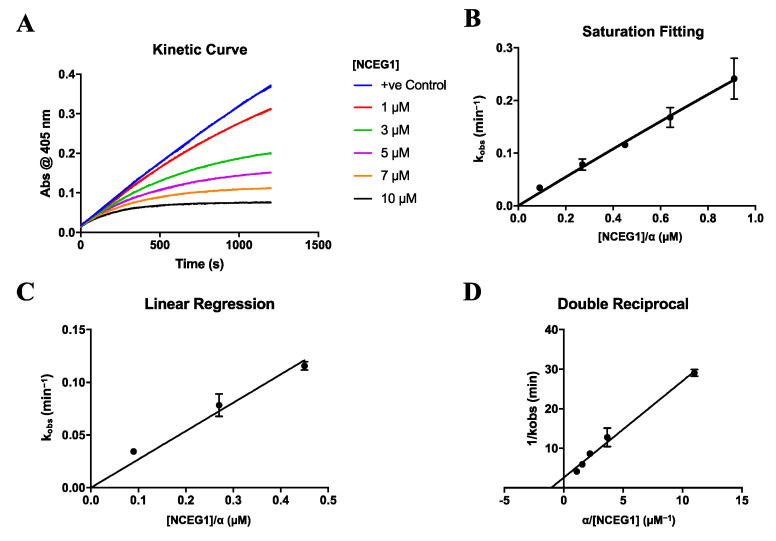
Representative kinetic data for inhibitors. Data shown for compound **17** with (**A**) time-dependent inactivation of TG2 (blank subtracted), (**B**) fitting of observed first-order rate constants to a hyperbolic model for saturation kinetics, (**C**) linear regression of lowest concentration rate constants, and (**D**) double reciprocal fitting of the data shown in (**B**).

Relative to the original scaffold, we expected the potency of these novel chemical biology tools to decrease slightly, due to the additional bulk incorporated on the glutamate sidechain. Although we expected the sidechain moiety to be directed into bulk solvent, we were concerned that its mass and charge would disfavor the binding interaction. Therefore, we kinetically characterized these probes as rigorously as possible, in order to determine their relative affinity and reactivity. For most inhibitors we have made, including the parent inhibitor AA9 (Figure 1), we have been able to measure kinetic parameters under Kitz and Wilson conditions [37]. This experimental approach is based on the use of a continuous activity assay [38] and the measurement of first-order rate constants for the time-dependent inactivation of enzyme (see Figure 2A and Figures S1–S5 in Supplementary Materials). The dependence of these observed rate constants (*k*_obs_) on the inhibitor concentration divided by alpha ([I]/α) can then be fitted to a hyperbolic saturation model where the upper plateau represents the rate constant of inactivation (*k*_inact_), and the concentration providing half the rate constant of inactivation represents *K*_I_ (Figure 2B) [26,27,39]. Aside from inhibitor **22**, the inhibitors disclosed herein exhibited excellent rates of inactivation; however, this assay was unable to provide reliable values for the observed rate constants (*k*_obs_) at high concentrations and, thus, was a poor saturation fitting. To compensate for this, the overall efficiency of the inhibitor (*k*_inact_/*K*_I_) can be derived from the slope of the linear region of the saturation plot (Figure 2C). To gauge the individual parameters, a double reciprocal fitting was also employed where *K*_I_ can be calculated from the x-axis intercept and *k*_inact_ can be calculated from the y-axis intercept (Figure 2D).

Despite our concerns, we were pleased to see that nearly all of our derivatives maintained excellent potency in their inhibition of TG2. In fact, the cell-impermeable inhibitors **17** and **18** were nearly threefold more potent than our original lead compound (AA9) (Table 1) [26]. We ascribe the increased affinity of these new inhibitors to the amino acid residue added to the peptidomimetic backbone, as recently described elsewhere [32]. The fluorescent probes also showed potent inhibition of TG2; with a *k*_inact_/*K*_I_ value of 1186 × 10^3^ M^−1^min^−1^, FITC derivative **23** is one of the most efficient TG2 inhibitors known. The coumarin probe **22** showed only modestly efficient inhibition of TG2, with a *k*_inact_/*K*_I_ value of 188 × 10^3^ M^−1^min^−1^, although it should be noted that this derivative displayed decreased solubility in aqueous solutions above 25 µM. The propargyl derivative **31** exhibited highly efficient inhibition of TG2 similar to the cell-impermeable inhibitors, with a *k*_inact_/*K*_I_ value of 497 × 10^3^ M^−1^min^−1^. The diversity of the cargo that can be attached at the glutamate residue, without abrogation of inhibitory efficiency, implies that this site can be broadly varied for numerous applications. Considering the putative binding mode [27] for these peptidomimetic inhibitors featured the naphthoyl moiety bound in the large hydrophobic binding pocket of TG2, it seems reasonable to hypothesize that the glutamate sidechain cargo must be directed out into solvent, where it has little effect on binding affinity. Molecular docking confirmed this hypothesis, with key intermediate **16**, cell-impermeable **18 (NCGE2)**, and fluorescent probe **29 (NCEG-RHB)** all having their sidechain and cargo directed away from the enzyme (Figure S6 in Supplementary Materials).

### 2.4. Isozyme Selectivity

In the development of probes for TGases, isozyme selectivity is always a significant challenge. Since the catalytic machinery of the active sites of all TGases features a conserved Gly-Gln-Cys-Trp-Val sequence, selectivity for one isozyme over the others requires exploitation of slight differences in the protein substrate binding sites [40,41]. In order to assess the isozyme selectivity of our cell-impermeable inhibitors, TG2 and four other therapeutically relevant TGases (Factor XIII, TG1, TG3, and TG6) were exposed to a concentration of inhibitor representing the same apparent competition with respect to the assay substrate (AL5 [38] or A101 [42,43]). In other words, both the substrate and inhibitor concentrations were varied so that the [I]/α values were identical [27]. Under these conditions, both **17** and **18** displayed excellent selectivity by irreversibly inactivating TG2 with no detectable inhibition of the other isozymes (see Figures S7 and S8 in Supplementary Materials). This provides confidence that in further applications, these cell-impermeable inhibitors will selectively target TG2 over other transglutaminases.

### 2.5. Pharmacokinetic Properties

The cell-impermeable inhibitors **17** and **18** were designed to violate virtually all of the rules for cellular permeability described by Lipinski and Veber [44,45]. They were also empirically evaluated with respect to their pharmacokinetic properties, in particular to determine their cell permeability. In a PAMPA test, the −Log Pe values were very high (Table 2). The measured values of >9.02 and >8.79 are much higher than the traditionally accepted upper limit of 6, suggesting these derivatives would show low permeability. Indeed, neither compound was even detected in the receptor compartment for the PAMPA, resulting in the lower limit of detection being reported as an approximate value. This suggests that cell permeability is negligible. In cultured MDCK cell permeability assays, both of these compounds exhibited very low permeability again, independent of the Pgp-mediated efflux pathway.

### 2.6. Fluorescent Labelling

To validate that the novel fluorescent probes were indeed labelling TG2 irreversibly, recombinantly expressed and purified human TG2 [47] was exposed to 30 µM of each probe for 25 min at room temperature. The samples were then analyzed by SDS-PAGE, revealing a fluorescent band at 78 kDa in each sample corresponding to full-length TG2. Upon irradiation at the relevant excitation wavelength, the fluorescent emission of each probe confirmed that TG2 was covalently labelled by each fluorescent probe (see Figure S9 in Supplementary Materials for full gel images). Given the high isozyme selectivity of the cell-impermeable inhibitors **17** and **18**, it is reasonable to assume the fluorescent probe versions would display similar selectivity and prove to be very useful for fluorescence microscopy.

**Figure 3:**
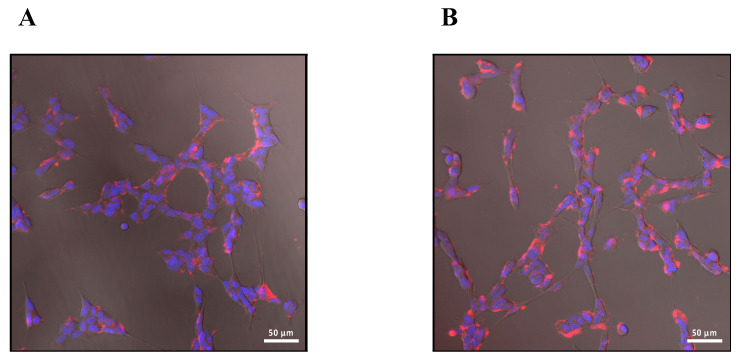
(**A**) Cell microscopy of SH-SY5Y cells incubated with 30 µM **29 (NCEG-RHB)** (red) for 30 min and nucleus stained with Hoechst (blue). (**B**) Cell microscopy of SH-SY5Y cells differentiated with 20 µM retinoic acid for 4 days, incubated with 30 µM **29 (NCEG-RHB)** (red) for 30 min and nucleus stained with Hoechst (blue).

We elected to use the rhodamine B-labelled **29** (**NCEG-RHB**) in subsequent fluorescence microscopy studies in SH-SY5Y cells due to its high solubility and desirable photochemical properties. SH-SY5Y cells can be differentiated upon the addition of retinoic acid, which induces overexpression of TG2 [48,49,50]. As shown in (Figure 3A), compound **29** (aka **NCEG-RHB**) is cell permeant and clearly visible in the cytoplasm, but not in the nucleus. A noticeable increase in red fluorescence is observed in cells pre-treated with retinoic acid treatment, suggestive of an increased expression and labelling of intracellular TG2 (Figure 3B).

### 2.7. Evaluation of Cancer Cell Proliferation and Migration

**Figure 4:**
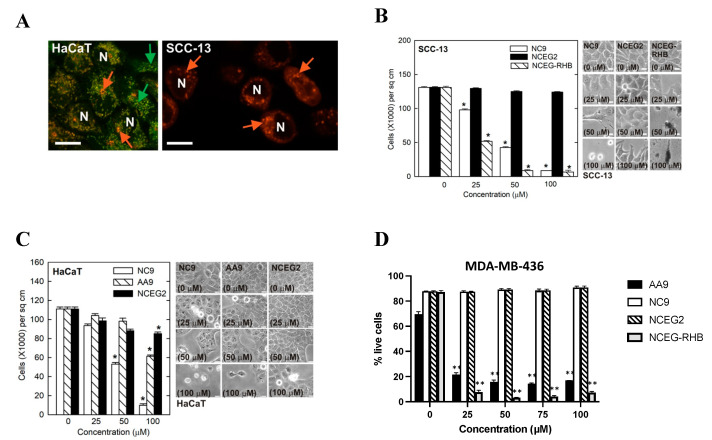
Treatment of cancer cells with cell-permeable and cell-impermeable inhibitors. (**A**) HaCaT and SCC-13 are epidermis-derived cutaneous squamous cell carcinoma cells that were treated for 10 h with NCEG-RHB prior to imaging using a spinning disc confocal microscope. NCEG-RHB localization is detected adjacent to the nuclei (N) in both cell types and is indicated by red arrows. The HaCaT cell cultures were also stained for 15 min with MitoTracker Green before imaging. MitoTracker Green detects mitochondria membrane proteins inside the cell and the labelling is indicated by green arrows. White scale bars = 100 µm. (**B**) SCC-13 cells treated with cell-impermeable inhibitor **18** (**NCEG2**) and cell-permeable inhibitors NC9, AA9, and **NCEG-RHB**. Scale bars represent 50 µm; (*) *p* value < 0.001 (**C**) HaCaT cells treated with cell-impermeable inhibitor **18** (**NCEG2**) and cell-permeable inhibitors NC9 and AA9. Scale bars represent 50 µm; (*) *p* value < 0.001. (**D**) MDA-MB-436 cells treated with cell-impermeable **18** (**NCEG2**) and with cell-permeable inhibitors NC9, AA9 and **NCEG-RHB**. Experiments were carried out in triplicate and are represented as average ± SD of the percentage of living cells; (**) *p* value < 0.01.

The library of research tools disclosed herein are all potent irreversible inhibitors of TG2, as measured in our biochemical activity assay (see above). However, these molecular tools differ markedly in their cell permeability, allowing us to apply these contrasting agents to determine whether the role of TG2 in the proliferation and migration of cancer cells is due to its intracellular or extracellular activity. The unique properties of our cell-impermeable inhibitors make them powerful tools to answer this longstanding question. Remarkably, cell-impermeable inhibitor **18** (**NCEG2**) did not have any effect on cell proliferation of MDA-MB-231, MDA-MB-436, MDA-MB-468, HaCaT, or SCC-13 cell lines at concentrations up to 100 µM (Figure 4 and Supplementary Materials Figure S10). In further support, **18** (**NCEG2**) failed to suppress migration of MDA-MB-436 and MDA-MB-231, a trait commonly associated with TG2 activity in cancer cells (Figure S11) [51]. The rhodamine B-labelled probe **29** (**NCEG-RHB**) was first validated as being cell permeable in SH-SY5Y, HaCat, and SCC-13 cells through fluorescence microscopy (see Figure 3 and Figure 4A). It was then tested in a similar manner to **18**, in SCC-13, MDA-MB-436, and MDA-MB-231 cells. Notably, in three different cancer cell lines, the fluorescent probe **29** (**NCEG-RHB**) also suppressed migration and proliferation significantly—even more so than previous lead inhibitors AA9 and NC9 (Figure 4B,D and Figure S10). In this regard, probe **29** (**NCEG-RHB**) enables both diagnostic and pharmacological applications [52]. In light of the markedly different effects observed for the cell-*permeable* inhibitor **29** compared to the cell-*impermeable* inhibitor **18**, we conclude that targeting the cancer-associated roles of TG2 requires the inhibition of *intracellular* TG2. Further, it is presumably the *intracellular G-protein activity* of TG2 that contributes to cancer progression, since these chemical tools were designed to irreversibly inactivate the transamidase active site, but also to block GTP binding by locking TG2 in its open conformation [26,27].

### 2.8. Pull-Down of TG2 from E. coli Lysate

While this manuscript was in preparation, Hauser et al. published a novel biotinylated TG2 inhibitor (Figure S12) that is highly efficient, exhibiting a *k*_inact_/*K*_I_ value of 7260 M^−1^s^−1^ (or 435,600 M^−1^ min^−1^) [53]. It is noteworthy that this inhibitor exploits the same binding pocket as our inhibitors disclosed herein, implying that this site of similar inhibitor scaffolds is broadly tailorable, with minimal impact on binding affinity. Our propargylated inhibitor **31** shows comparable efficiency to Hauser’s (see Table 1). Further, TG2 that has been labelled with inhibitor **31** should be amenable to incorporation of a wide variety of azide-functionalized cargo, including desthiobiotin, which would allow pull-down with streptavidin resin and subsequent elution under milder conditions than its biotinylated counterpart. More specifically, incorporating desthiobiotin instead of biotin allows for elution from streptavidin resin using low millimolar concentrations of biotin for competitive displacement from the streptavidin binding site instead of the harsh denaturing conditions required for typical biotinylated chemical tools [54]. Inhibitor **31** may, therefore, allow for more protein–protein interactions to be retained during the elution process in the discovery of novel TG2 interactions.

As a proof of concept, previously described BL21 *E. coli* cells were transformed with a plasmid (pGST-PSP-rhTG2) to induce overexpression of recombinant human TG2 (rhTG2) as a GST fusion protein [47]. The bacterial cells were then lysed and exposed to the propargylated inhibitor **31** to allow for labelling. A subsequent CuAAC reaction was then performed in the same cell lysate to incorporate the desthiobitoin-PEG3-azide into labelled TG2. The lysate was then passed over streptavidin resin to isolate all proteins that had reacted with inhibitor **31**. These proteins were then eluted from the resin using a solution of 4 mM biotin, and the eluant was analyzed by SDS-PAGE. As can be seen in Figure S13 in the Supplementary Materials, the only protein efficiently isolated in this experiment corresponds to the GST-TG2 fusion protein (MW = 25 kDa + 78 kDa). This validates not only the TG2-selectivity of probe **31**, but also the efficiency of the click reaction of the GST-TG2-**31** adduct, allowing for the isolation of TG2 from cell lysate in a very mild manner.

## 3. Materials and Methods

### 3.1. Chemical Synthesis

All details and experimental procedures for the chemical synthesis executed within this study along with characterization data can be found in the Supplementary Materials [55,56].

### 3.2. Molecular Docking of Compounds ***16***, ***18***, and ***29*** into TG2 Active Site

The “compute” tool from MOE was used to perform docking analysis of each ligand with TG2 (PDB: 2Q3Z) one by one, following a non-covalent approach where ligand placement was achieved using the Triangle Matcher protocol (London dG) to produce 30 poses. In addition, a rigid receptor refinement protocol was performed (GBVI/WSA dG) and a total of 5 final poses were obtained. Finally, using the builder tool from MOE, the covalent bonds between residue CYS277 and the acrylamide warhead of the bound inhibitors were manually created, prior to minimization of the system (to 0.001 kcal mol^−1^ Å^2^).

### 3.3. rhTG2 Inhibition Assay

Recombinant human TG2 was expressed and purified using previously published protocols [47]. The inhibition was monitored using a continuous chromogenic substrate AL5 [38] under Kitz and Wilson conditions [37,57]. In brief, 125 µL of assay buffer composed of 111.11 mM MOPS, 15.56 CaCl_2_ pH 6.9 was added to a 1.5 mL Eppendorf tube to achieve final assay concentrations of 50 mM MOPS and 7.5 mM CaCl_2_. To the tube was then added the respective concentration of inhibitor from an aqueous working stock (<5% DMSO final conditions). Various volumes of water were then added to ensure the final volumes were equal. The AL5 substrate was added to the Eppendorf tube as a 5 µL 5.56 mM solution in DMSO to obtain a final assay concentration of 100 µM. A 96-well polystyrene microplate then had 180 µL of the assay mixture added to it. The enzymatic reaction was finally initiated by addition of 20 µL 5 mU prediluted TG2 in assay buffer using a multichannel pipette. The reaction was monitored at 405 nm using a BioTek Synergy 4 plate reader for 20 min at 25 °C. A positive control with no inhibitor and a blank with no enzyme nor inhibitor were also included. The blank subtracted kinetic curves were then analyzed. The observed first-order rate constants of inactivation were gathered through a one-phase association model using the GraphPad software package. The rate constants were then fit to various models to elucidate the inhibition parameters. If saturation was achieved, they were fit to a saturation model versus the inhibitor concentration divided by alpha (where $\alpha =1+\frac{\left[Substrate\right]}{Km}$). A linear regression of the linear region of this saturation fitting was calculated to obtain a ratio of *k*_inact_/*K*_I_. A further double reciprocal model was used if saturation could not be observed due to the limitations in sensitivity of the assay or if solubility issues were present.

### 3.4. TGase Isozyme Selectivity

The selectivity of the cell-impermeable inhibitors was evaluated against four other therapeutically relevant TGases (TG1, TG3a, TG6, and FXIIIa, all purchased from Zedira GmbH (Darmstadt, Germany). By varying both the inhibitor and assay substrate concentrations with each isozyme, we ensured that the [I]/α values remained constant as each isozyme had a different K_m_ for its respective substrate. In order to obey Kitz and Wilson conditions, the substrate concentrations were varied, and the inhibitor concentrations were adjusted to account for this change in affinity. TG1, TG6, and TG2 were all monitored using the aforementioned rhTG2 inhibition assay procedure. The AL5 substrate concentrations for TG1 and TG6 were 112 µM and 435 µM, respectively, with 0.1 µM TG1 or 0.32 µM TG6. To monitor the activity of TG3a and TG6, a FRET-quenched substrate A101 (Zedira Gmbh) was used [42,43]. In brief, 125 µL assay buffer #1 (containing TRIS, CaCl_2_, and NaCl pH 7.5), 18 µL assay buffer #2 (containing TCEP and acceptor substrate H-Gly-OMe pH 7.5), and a respective concentration of inhibitor aqueous working stock balanced with various volumes of water were combined in a 96-well black plate with clear bottom/top. The enzymatic reactions were then initiated by addition of 20 µL of prediluted enzyme with a microchannel pipette to provide 0.11 µM FXIIIa or 0.17 µM TG3a. The isopeptidase activity of the respective TGase was then monitored at Ex/Em = 313/418 nm with a BioTek Synergy 4 plate reader at 25 °C. The final assay conditions contained a final volume of 200 µL 69 mM TRIS, 10 mM CaCl_2_, 208 mM NaCl, 5 mM TCEP, and 13 mM H-Gly-OMe pH 7.5. A positive control with no inhibitor and a blank with no enzyme nor inhibitor were included. The kinetic curves then had the blank subtracted and were compared versus the other isozymes.

### 3.5. Fluorescent Labelling of Purified rhTG2

Recombinant human TG2 was expressed and purified from *E. coli* using a previously in-house-developed method [47]. The purified TG2 was stored in cleavage buffer (20 mM TRIS, 150 mM NaCl, 1 mM EDTA, 1 mM TCEP, 15% glycerol, pH 7.2) at −80 °C. The concentration obtained was 1.166 mg/mL (~15 µM TG2) by a Bradford assay. Totals of 10 µL of this stock, 5 µL of a 20 mM TRIS buffered solution of 50 mM CaCl_2_ (pH 7.2), and 10 µL of a 75 µM stock of the respective fluorescent probe (2.5% DMSO in mQ H_2_O) were added to a 1.5 mL Eppendorf tube and then vortexed. The labelling was allowed to occur for 25 min at room temperature. A total of 25 µL of a 2× Laemelli buffer with 5% BME was added to the Eppendorf tube, and the tube was heated to 100 °C for 5 min to ensure denaturation. The solutions were then allowed to cool and 20 µL was loaded onto a BioRad Mini-PROTEAN TGX 4–20% polyacrylamide precast SDS-PAGE gel along with 10 µL BioRad Precision Plus Protein Unstained Standards. Electrophoresis (120 V) was then performed for 1 h in 25 mM TRIS, 192 mM glycine, 0.1% SDS buffer. The gel was first visualized using a BioRad ChemiDoc MP Imager exciting at each specific wavelength (**23** blue epi illumination 530/28 nm filter, **22** UV trans illumination 530/28 nm filter, and **29 (NCEG-RHB)** green epi illumination 605/50 nm filter) to observe the fluorescent bands, and then the gel was stained with Coomassie Blue and visualized again.

### 3.6. SH-SY5Y Fluorescent Microscopy

SH-SY5Y cells (CRL-2266, ATCC) were thawed and grown under cell culture conditions (5% CO_2_, 37 °C, humidified) to 80% confluency in Dulbecco’s Modified Eagle Medium (DMEM) supplemented with penicillin/streptomycin (P/S, 100 U/mL/100 µg/mL) and heat-inactivated fetal bovine serum (FBS, 10%). Cells were passaged three times before being seeded into 12-well glass-bottom imaging plates. Cells were either grown to confluency using the previously mentioned methods or differentiated using an adapted protocol from Singh et al. [50]. Briefly, cells to be differentiated were seeded at 20% confluency and grown in DMEM supplemented with 3% heat inactivated FBS, P/S, and 20 µM trans-retinoic acid (RA). The medium was changed daily for 4 days, at which point cells were 80% confluent and used for experimentation.

Triplicate wells of both differentiated and undifferentiated cells, once 80% confluent, were treated with or without 20 µM of probe in their respective medium containing 0.1% DMSO and incubated under cell culture conditions for 2 h. Following incubation, cells were washed three times with 37 °C, sterile Dulbecco’s phosphate-buffered saline (PBS) and incubated for 20 min in phenol red-free medium with 1 µg/mL Hoechst 33342 under cell culture conditions. Each well was imaged on a Zeiss LSM 880 confocal microscope with both 20× and 63× objectives, simultaneously collecting both brightfield, nuclear fluorescence (Hoechst 33342, ex/em 405/461 nm) and any fluorescence associated with the remaining intracellular probe (ex/em 514/600 nm).

### 3.7. Cell Proliferation Assay

MDA-MB-468 cells were cultured in Dulbecco’s Modified Eagle Medium (DMEM)/F-12 DMEM, Gibco Laboratories, New York, NY, USA), 10% FBS (Gibco Laboratories, New York, NY, USA), 50 U/mL penicillin, and 50 μg/mL streptomycin (Gibco Laboratories, New York, NY, USA), while MDA-MB-231 and MDA-MB-436 cells were cultured in DMEM High Glucose with/stable L-Glutamine (EuroClone, Pero, MI, Italy), 10% FBS, and antibiotics, all grown at 37 °C and in 5% CO_2_ humidified atmosphere.

The compounds were added to the cultures at the concentrations of 25, 50, 75, and 100 μM and 0.1% DMSO represented the negative control. After 48 h, the cells were trypsinized for 2 min at 37 °C, trypsin was inactivated using 1 mL of the recovered supernatants (containing 10% FBS), centrifuged at room temperature 5 min at 1200 rpm, washed with PBS and resuspended in complete medium. Finally, 50 μL of cells were diluted in 500 μL of Count & Viability Reagent (Luminex, Prodotti Gianni, Milan, Italy) and analyzed by MUSE^®^.

The experiments were carried out in triplicate and the average ± SD of the percentage of living cells was reported. Statistical analysis was performed calculating *p* value by a Student’s *t*-test, two-tailed, with homovariance and significance expressed by (*) *p* value < 0.05.

### 3.8. Real-Time Cell Migration Assay

We assayed the motility of MDA-MB-436 cells in the presence of vehicle or TG2 inhibitors **18 (NCEG2)**, **19 (NCEG-RHB)**, and NC9 at 25 μM concentration with the xCELLigence RTCA system (Real-Time Cell Analyzer System, Acea Biosciences Inc., San Diego, CA, USA) [58]. About 3 × 10^5^ cells/wells were put into the top chambers of CIM-16 plates and the bottom chambers were filled with medium containing 5% FBS (Gibco Laboratories, New York, NY, USA) as a chemoattractant. Signal detection was done every 15 min for 24 h and each determination was performed in triplicate. Impedance values were expressed as a dimensionless parameter (Cell Index, CI), and values greater than 0.1 were considered positive. The rate of cell migration was also quantified by calculating the steepness, inclination, gradient, and changing rate of the CI curves over time (Slope).

### 3.9. HaCaT and SCC-13 Cell Staining

SCC-13 and HaCaT cells (0.1 × 10^6^) were plated in 35 mm Mat Tek glass bottom cell culture dishes. After 24 h, the cells were treated with 25 µM **18** (**NCEG-RHB**), a rhodamine-B-labelled cell-permeable TG2 inhibitor, for 18 h. The cells were washed three times with phosphate-buffered saline prior to spinning disc confocal microscopy. In addition, the HaCaT cells were treated with 50 nM MitoTracker GreenFM (#M7514) dye, obtained from Invitrogen (Waltham, MA, USA). The cells were then washed three times with PBS before live cell imaging using a Nikon spinning disc confocal microscope. HaCaT and SCC-13 are epidermis-derived cutaneous squamous cell carcinoma cells [59,60]. **NCEG-RHB** (**18**) is detected adjacent to the nuclei (**N**) in both cell types and is indicated by red arrows. MitoTracker GreenFM stains mitochondria membrane proteins inside the cell, which is indicated by the green arrows. The sizing bar in the images represents 100 microns.

### 3.10. Pull-Down of rhTG2 from E. coli Lysate

A total of 400 µL of the aforementioned *E. coli* cell lysate combined with 133 µL of a solution of 75 µM **31** was added to a 1.5 mL Eppendorf tube. The labelling was allowed to occur for 25 min. Totals of 25 µL of a solution of 500 µM azide-PEG3-desthiobiotin **32** and 500 µM cupric sulfate in 20 mM HEPES at pH 8.0 were added to the tube. To initiate the click reaction, 100 µL of 150 µM sodium ascorbate solution in mQ H_2_O was added and gently rocked for 2.5 h at room temperature. Using 100 µL slurry of equilibrated (washed 3× with PBS) streptavidin agarose resin in PBS (pH 7.4), 200 µL of the click reaction mixture was combined in an Eppendorf tube. The tube was gently mixed, and binding was allowed to occur for 0.5 h at room temperature with gentle rocking. The tube was then centrifuged (500× *g* (3000 rpm) for 1 min) to pellet the resin and the supernatant was removed and saved for the gel (FT1). An additional 100 µL of PBS buffer (pH 7.4) was added and the tube was gently mixed, and the resin was pelleted again. The supernatant was removed and saved for the gel (FT2). The wash with PBS buffer was then repeated once more (FT3). To elute the protein from the resin, 50 µL of elution buffer containing 4 mM biotin in PBS (pH 7.4) was added. The tube was gently mixed and allowed to gently shake at 37 °C for 10 min. To resin was pelleted and the supernatant was removed and saved for the gel (EL1). The elution step was then repeated two more times (EL2 and EL3).

For SDS-PAGE analysis of the elution, 15 µL of each fraction (or 15 µL of raw cell lysate) was combined with 15 µL 2× Laemelli buffer (5% BME) and boiled at 100 °C for 5 min for denaturation. The wells of a BioRad Mini-PROTEAN TGX Stain-Free 4–15% polyacrylamide precast SDS-PAGE gel were then loaded with 20 µL of the corresponding sample along with 10 µL of BioRad Precision Plus Protein Unstained Standards. Electrophoresis (120 V) was performed for 50 min and the gel was visualized by Coomassie staining.

## 4. Conclusions

In this work, we have disclosed novel chemical tools designed to selectively label tissue transglutaminase (TG2). The cell-impermeable inhibitors described herein are first-in-class inhibitors that display excellent potency and efficiency of TG2 inhibition in addition to confirmed cellular impermeability. As such, they should prove to be powerful tools for the selective inhibition of the extracellular activities of TG2. Fluorescent probes were also prepared and shown to be highly efficient at labelling TG2, and to be cell permeable. A propargylated inhibitor was also designed and used to irreversibly inactivate TG2, which was then modified by a subsequent click reaction, incorporating desthiobitoin and allowing TG2 to be pulled down from cell lysate.

These probes should all prove to be of broad utility for investigations of the biological roles of TG2. However, the most important conclusion from this work may be from our interrogation of the relative importance of extracellular and intracellular TG2 activity in the propagation and invasion of certain cancer cells. Direct comparison of the results obtained with cell-impermeable inhibitor **18** (**NCEG2**) with those of cell-permeable inhibitor **29** (**NCEG-RHB**) provides the first concrete evidence that *intracellular* TG2 activity contributes to the cancer-associated phenotype, at least in the cell lines studied herein. This validates the rationale for specifically targeting intracellular TG2 when trying to alter cancer progression and advances our understanding of the importance of the sub-cellular context. The future application of these chemical tools should allow further discovery of the sub-cellular roles of TG2 and lead to other therapeutic applications.

## Figures and Tables

**Table 1:**
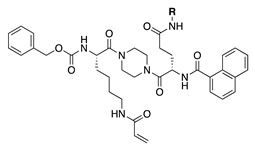
Kinetic data for cell-impermeable inhibitors, fluorescent probes, and propargyl inhibitors.

Compound	R	*K*_I_ (µM)	*k*_inact_ (min^−1^)	*k*_inact_/*K*_I_ (×10^3^ M^−1^ min^−1^)
**17** *	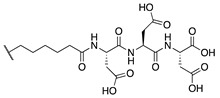	1.0 ± 0.3	0.39 ± 0.10	409 ± 22
**18** (**NCEG2**) *	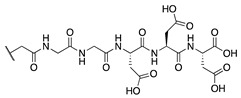	4.1 ± 2.1	2.33 ± 1.12	563 ± 13
**22**	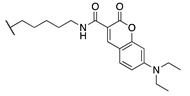	1.3 ± 1.1	0.24 ± 0.07	188 ± 167
**23**	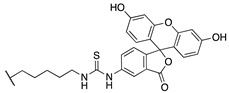	n.d.	n.d.	1186 ± 91 **
**29** (**NCEG-RHB**)	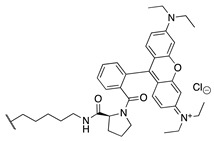	n.d.	n.d.	223 ± 3 **
**31** *	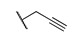	0.9 ± 0.5	0.46 ± 0.26	497 ± 33

* Kinetic data fitted to a double reciprocal model; ** Kinetic data from lowest concentrations of saturation plot fitted to a linear model.

**Table 2:** Representative pharmacokinetic properties for cell-impermeable inhibitors **17** and **18**.

Compound	17	18 (NCEG2)
cLog P *	0.94	−1.19
Log D	<−2.73	<−2.63
−Log P_e_	>9.02	>8.79
P_app(A-B)_ (10^−6^, cm/s)	0.31	0.47
P_app(B-A)_ (10^−6^, cm/s)	0.42	0.56
Efflux Ratio	1.39	1.18
P_app(A-B)_ (10^−6^, cm/s) + Pgp Inh.	0.33	0.57
P_app(B-A)_ (10^−6^, cm/s) + Pgp Inh.	0.35	0.36
Efflux Ratio + Pgp Inh.	1.04	0.66

* cLog P was calculated using SwissADME online web tool [46].

## Data Availability

Data will be provided upon reasonable request.

## Supplementary Materials

The following supporting information can be downloaded at: https://www.mdpi.com/article/10.3390/ijms241612546/s1.
