# Supplementary material for: Cell-Impermeable Inhibitors Confirm That Intracellular Human Transglutaminase 2 Is Responsible for the Transglutaminase-Associated Cancer Phenotype
Source: Int J Mol Sci. 2023 Aug 8;24(16):12546. doi: 10.3390/ijms241612546 (PMC10454375; doi:10.3390/ijms241612546)
Supplement: Supplementary file 1 [file ijms-24-12546-s001.zip › ijms-2482970-supplementary.pdf]

# Supporting Information

*for*

# Cell Impermeable Inhibitors Confirm that Intracellular Human Transglutaminase 2 is Responsible for the Transglutaminase-Associated Cancer Phenotype

*Eric W. J. Gates[1], Nicholas D. Calvert[1], Nicholas J. Cundy[1], Federica Brugnoli[2], Pauline Navals[1], Alexia Kirby[1], Nicoletta Bianchi[2], Gautam Adhikary[3], Adam J. Shuhendler[1], Richard L. Eckert[3], Jeffrey W. Keillor[1]**

[1]Department of Chemistry and Biomolecular Sciences, University of Ottawa, Ottawa, Ontario K1N 6N5, Canada

[2]Department of Translational Medicine, University of Ferrara, Ferrara, 44121, Italy

[3]Department of Biochemistry and Molecular Biology, University of Maryland School of Medicine, Baltimore, Maryland 21201, United States

**Table of Contents**

Supplementary synthetic schemes ........ 3
Supplementary kinetic traces and fitting ........ 6
Molecular docking of compounds in TG2 active site ........ 11
Isozyme selectivity ........ 12
Supplementary fluorescent labelling images ........ 14
Supplementary cellular evaluation figures ........ 15
Biotinylated inhibitor published by Hauser *et al.* ........ 17
SDS-PAGE of pull-down from *E. coli* ........ 17
Synthesis and characterization of intermediates and final compounds ........ 18
NMR spectra of intermediates and final compounds ........ 27
HPLC traces of final compounds ........ 40
References ........ 47

## Supplementary synthetic schemes

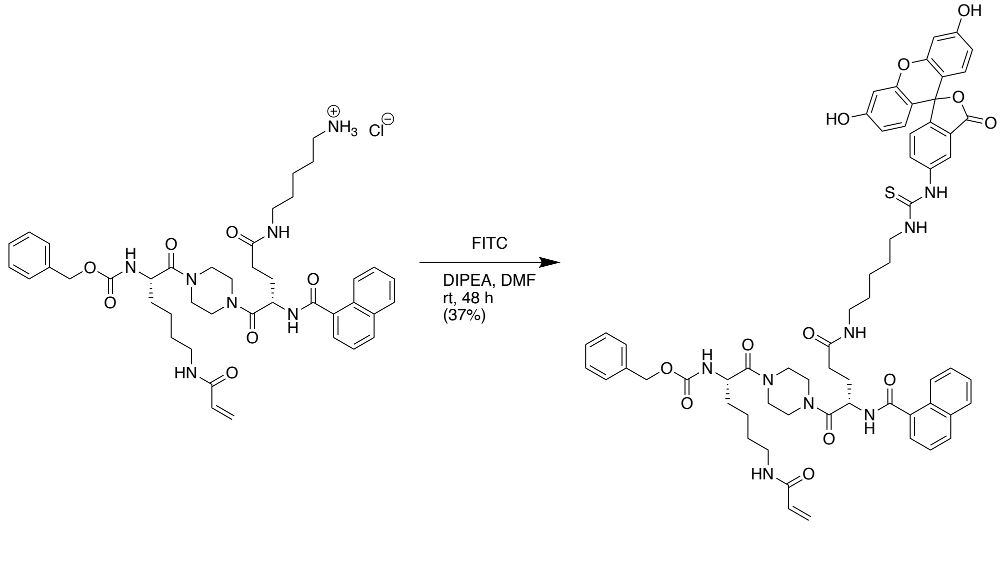


20 23

**Scheme S1.** Synthetic scheme from key intermediate to generate fluorescent FITC probe **23**.

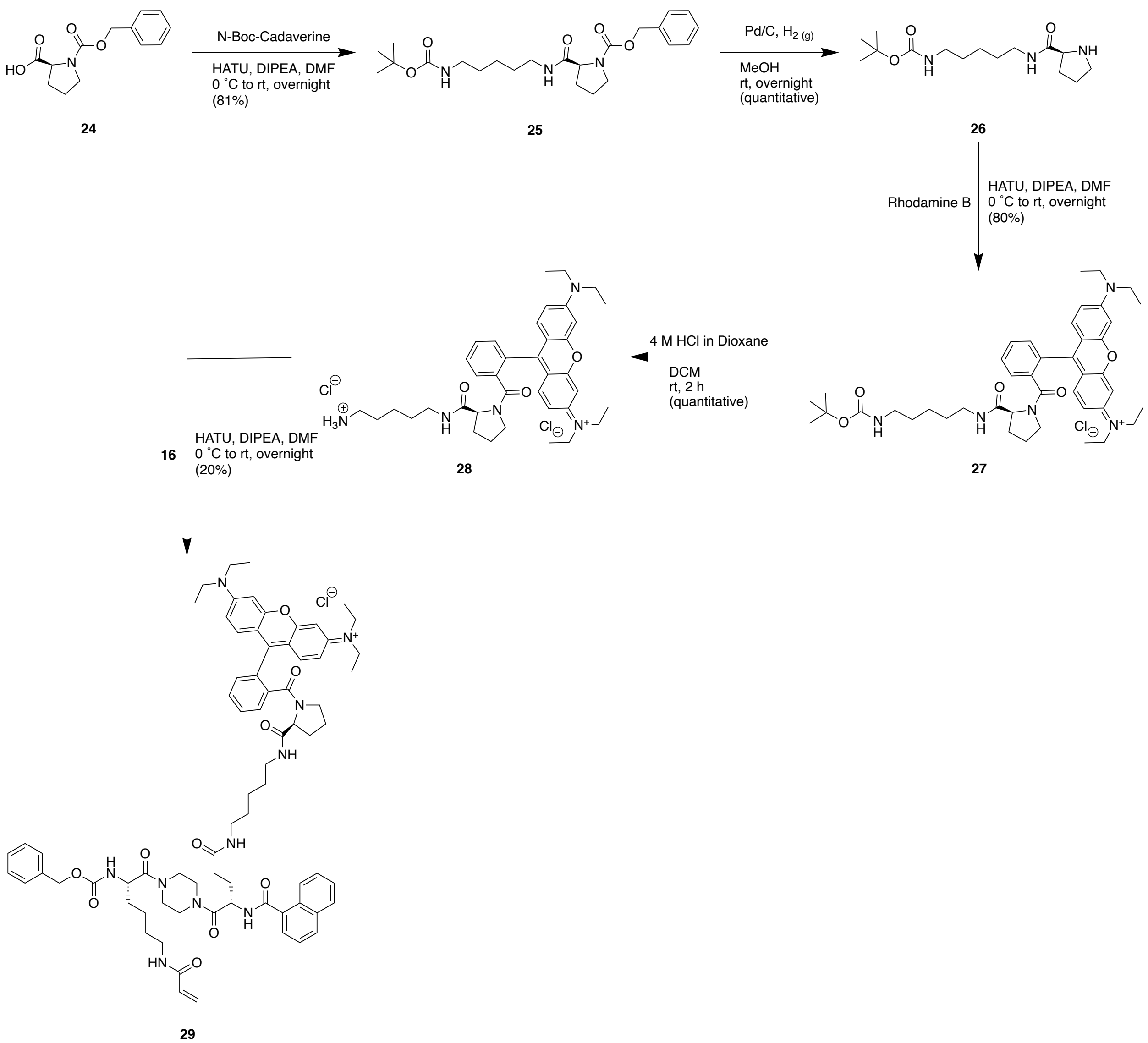


**Scheme S2.** Synthetic scheme from key intermediate **16** to generate fluorescent rhodamine B probe **29**.

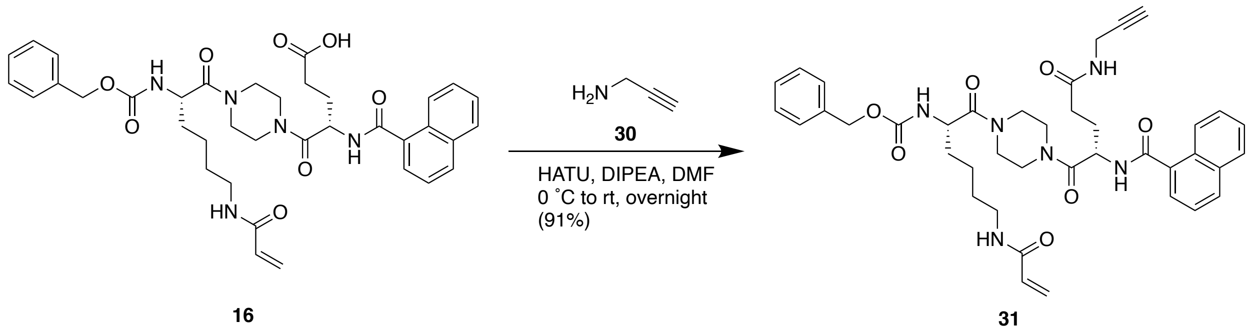


**16** **31**

**Scheme S3.** Synthetic scheme from key intermediate **16** to generate propargyl derivative **31**, appropriate for subsequent ‘click’ modification.

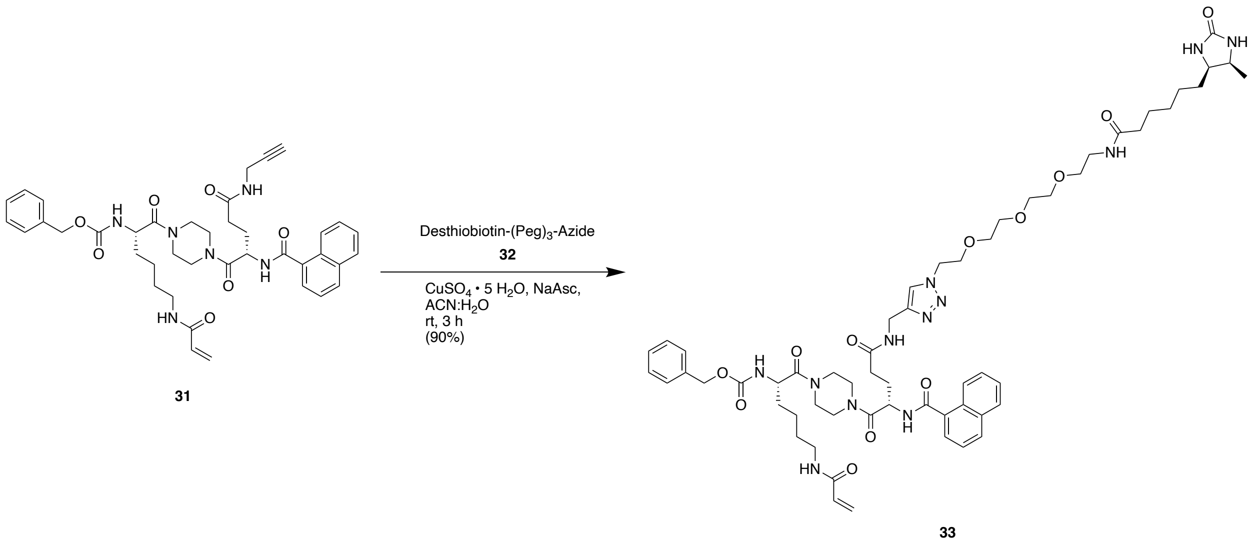


**31** **33**

**Scheme S4.** CuAAC ‘click’ reaction to generate the desthiobiotin derivative **33** used in the pull-down assay.

## Supplementary kinetic traces and fitting

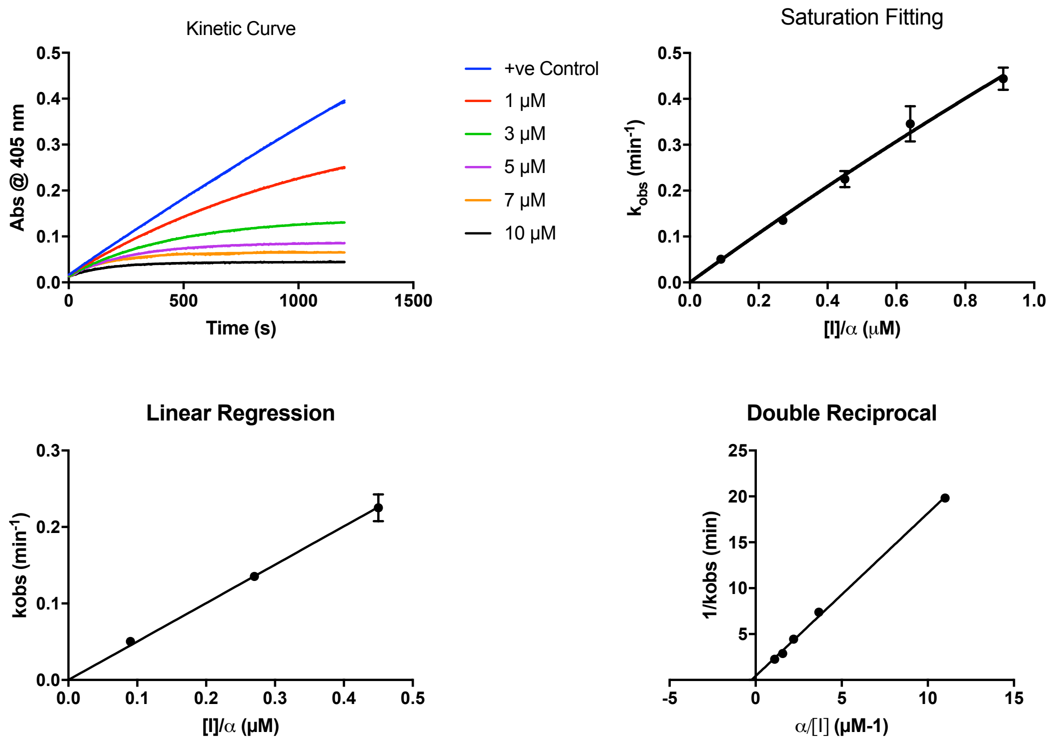


**Figure S1.** Kinetic traces and fitting of **18 (NCEG2)**.

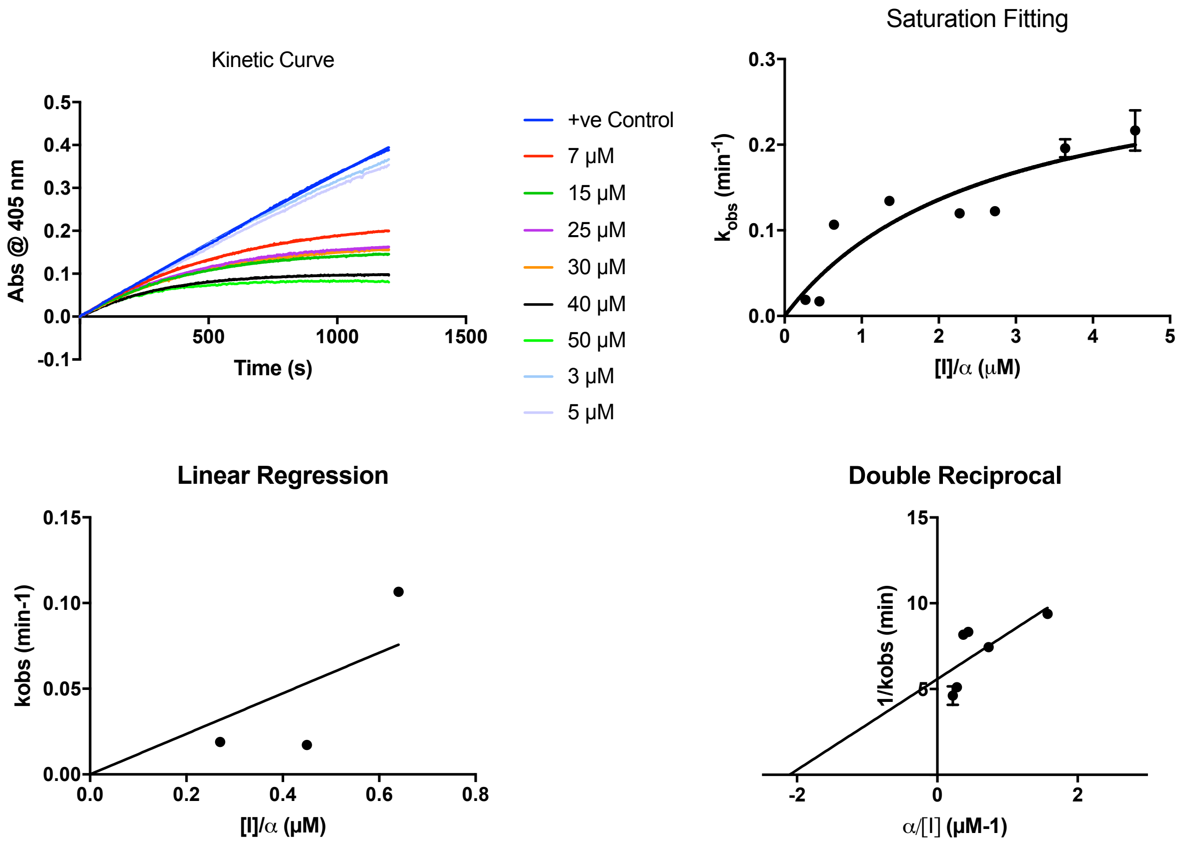


**Figure S2.** Kinetic traces and fitting for **22**.

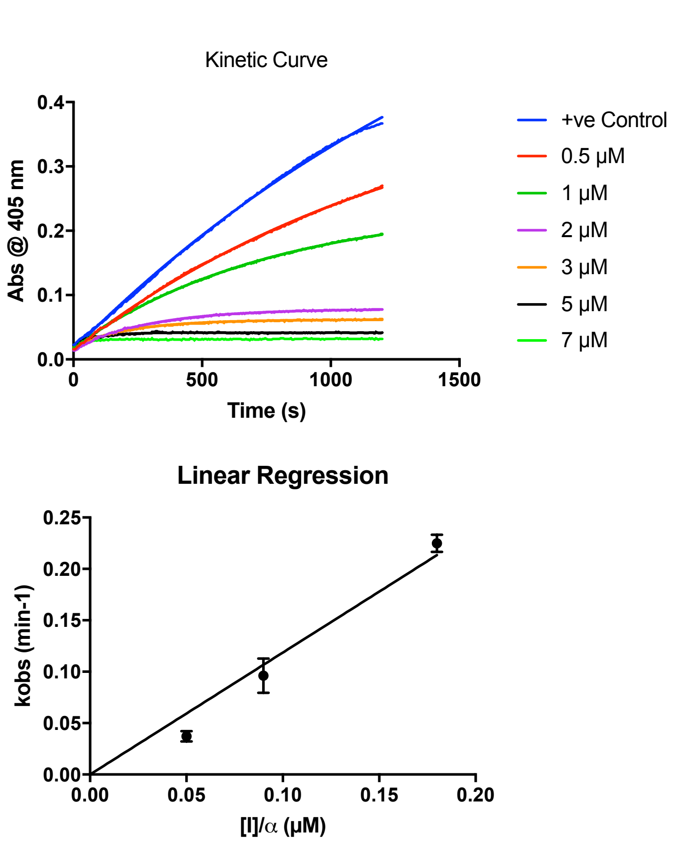


**Figure S3.** Kinetic traces and fitting for **23**.

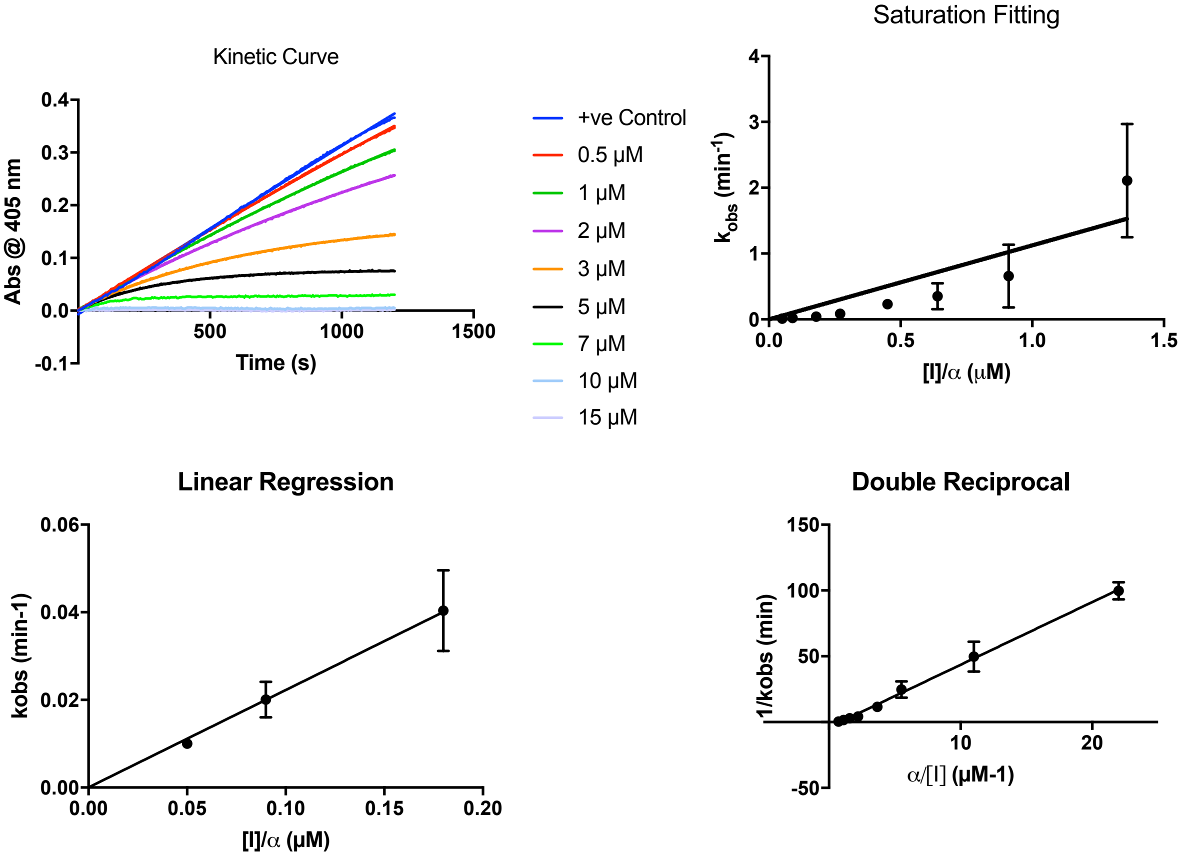


**Figure S4.** Kinetic traces and fitting for **29 (NCEG-RHB)**.

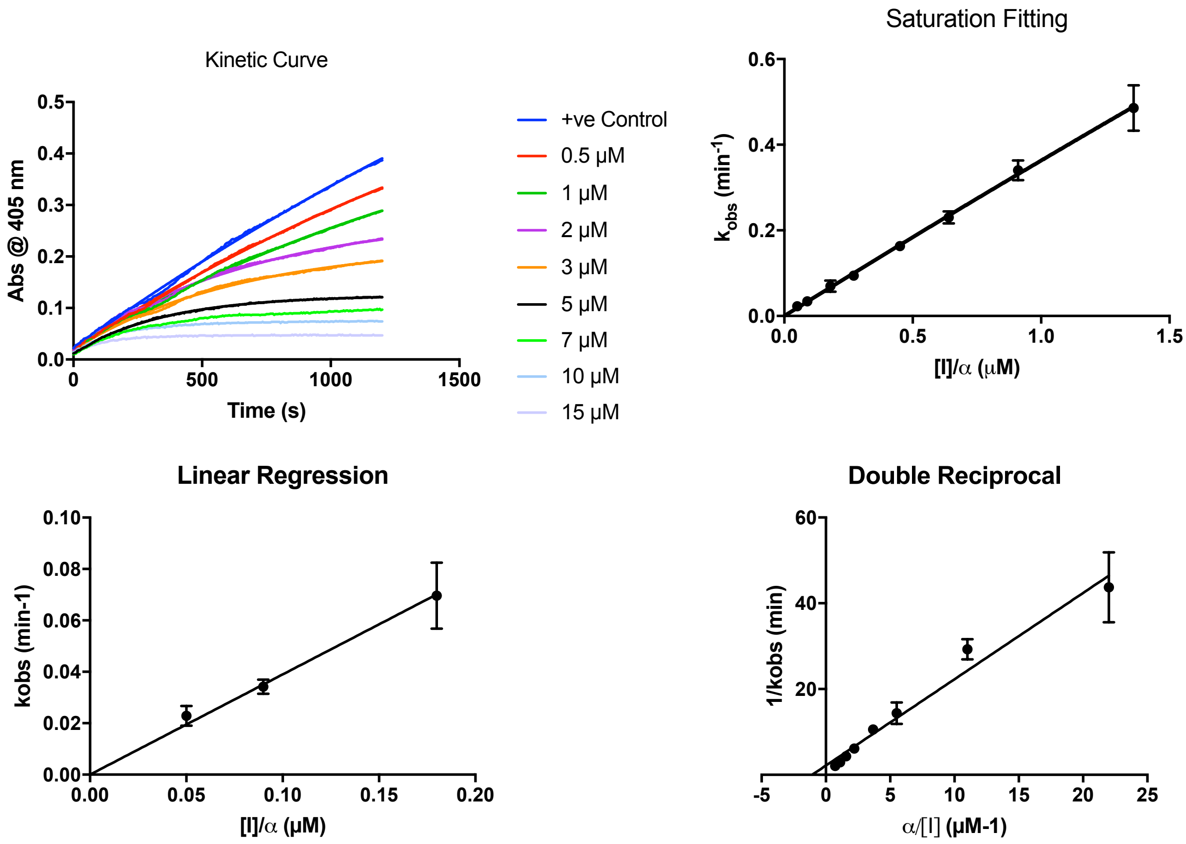


**Figure S5.** Kinetic traces and fitting for **31**.

**Molecular docking of compounds in TG2 active site**

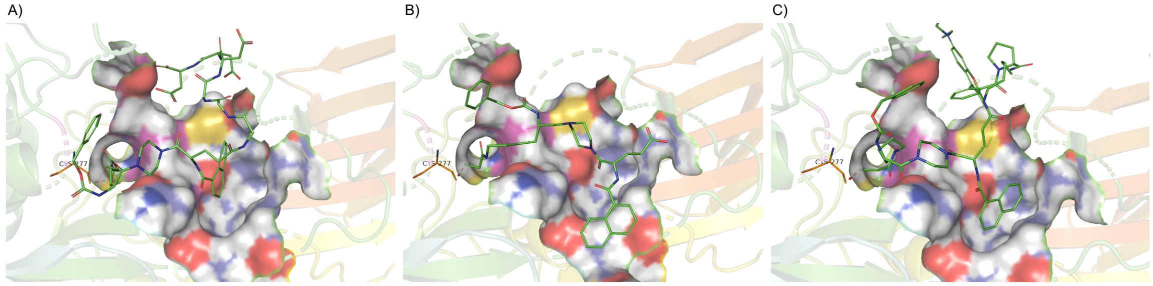


**Figure S6.** Structural representation of inhibitors **16**, **18 (NCEG2)**, and **29 (NCEG-RHB)** covalently bound to the catalytic pocket of TG2 (PDB: 2Q3Z) via CYS277 (orange residue). A) inhibitor **18**, S score =9.63. B) inhibitor **16**, S score -9.92. C) inhibitor **29**, S score -10.93.

## Isozyme selectivity

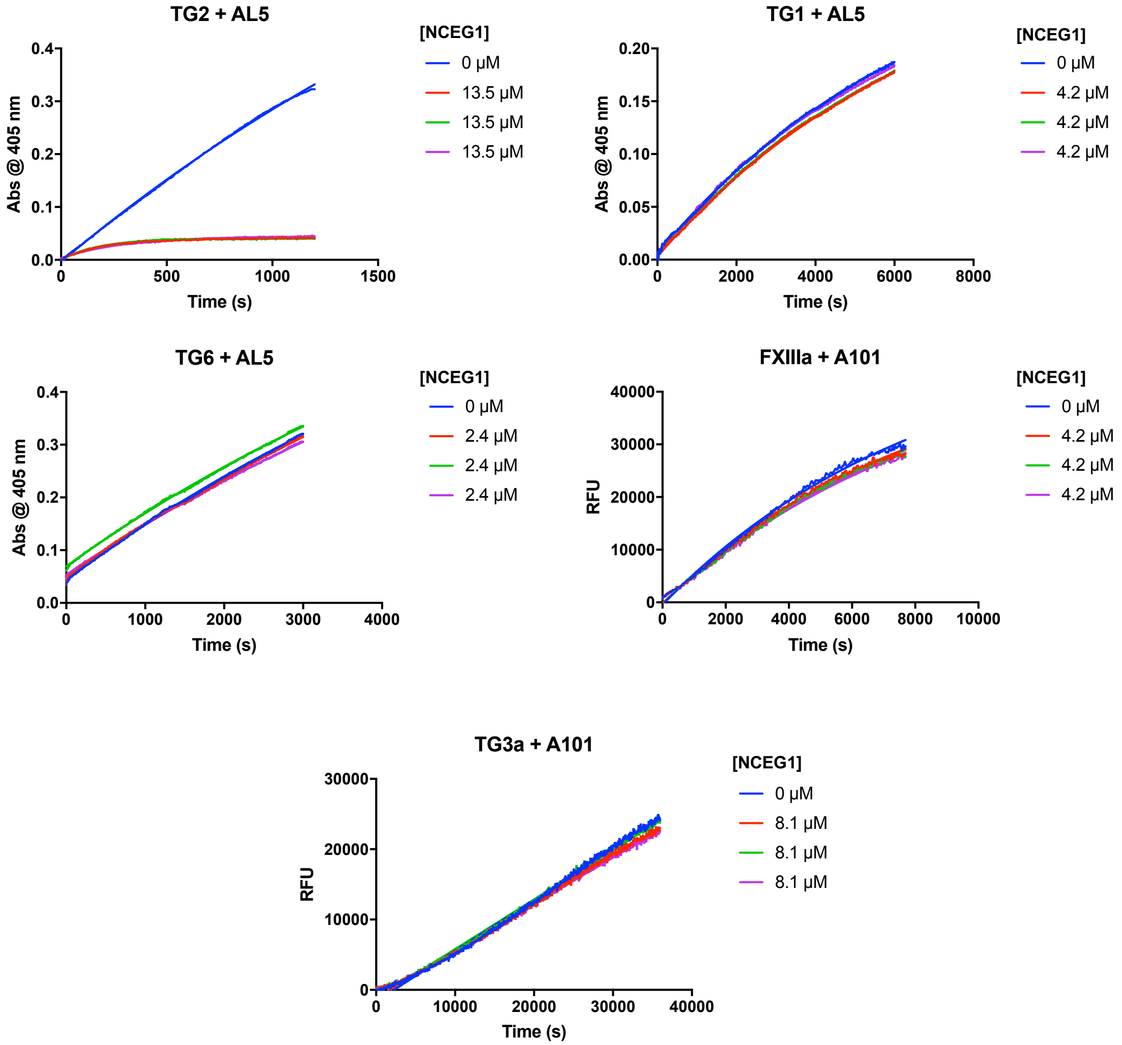


**Figure S7.** Isozyme selectivity of **17** with respect to therapeutically relevant human transglutaminases TG2, TG1, TG6, FXIIIa, and TG3a, using either chromogenic substrate AL5 or fluorescently quenched isopeptidase substrate A101.

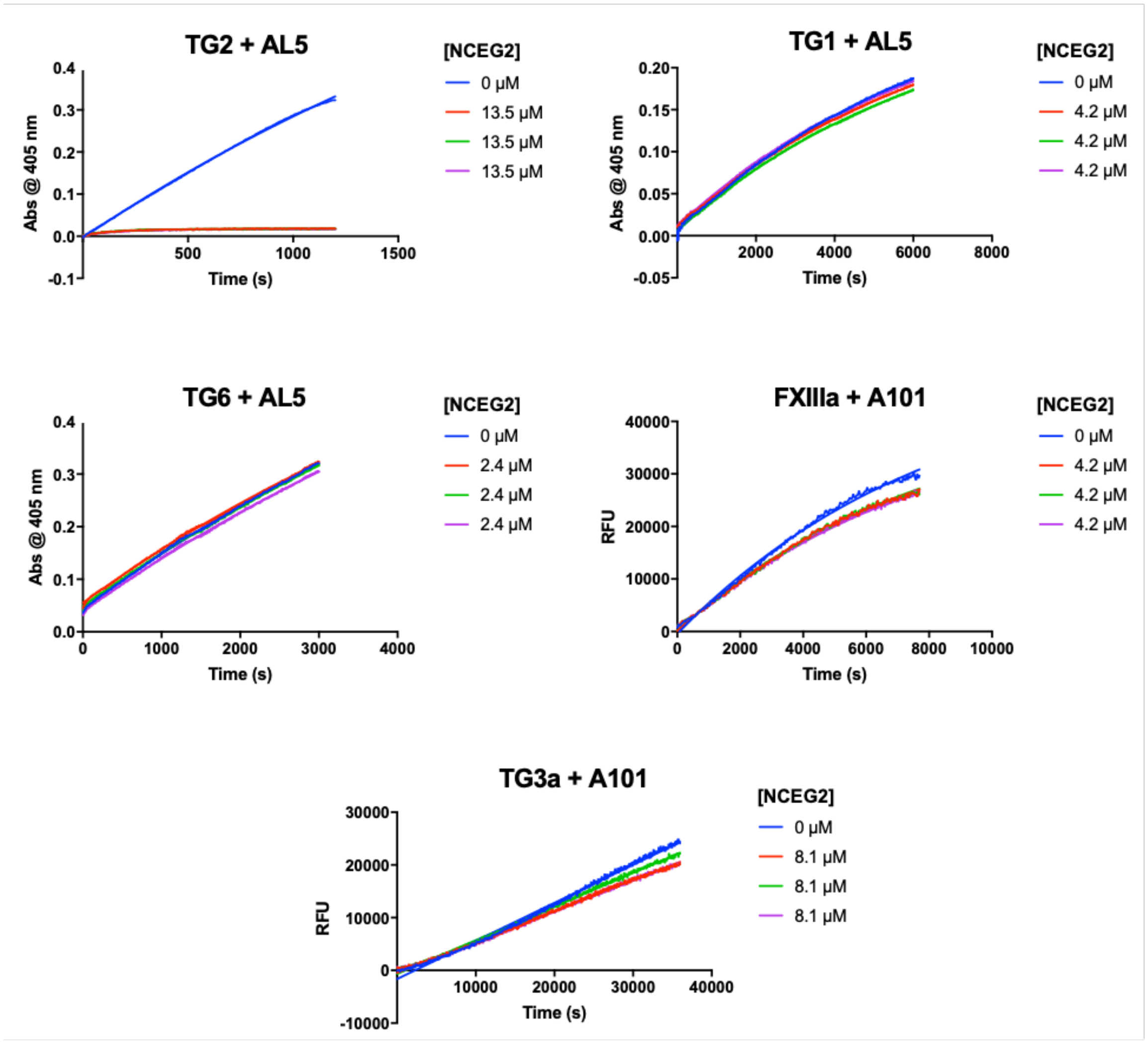


**Figure S8.** Isozyme selectivity of **18** (**NCEG2**) with respect to therapeutically relevant human transglutaminases TG2, TG1, TG6, FXIIIa, and TG3a, using either chromogenic substrate AL5 or fluorescently quenched isopeptidase substrate A101.

## Supplementary fluorescent labelling images

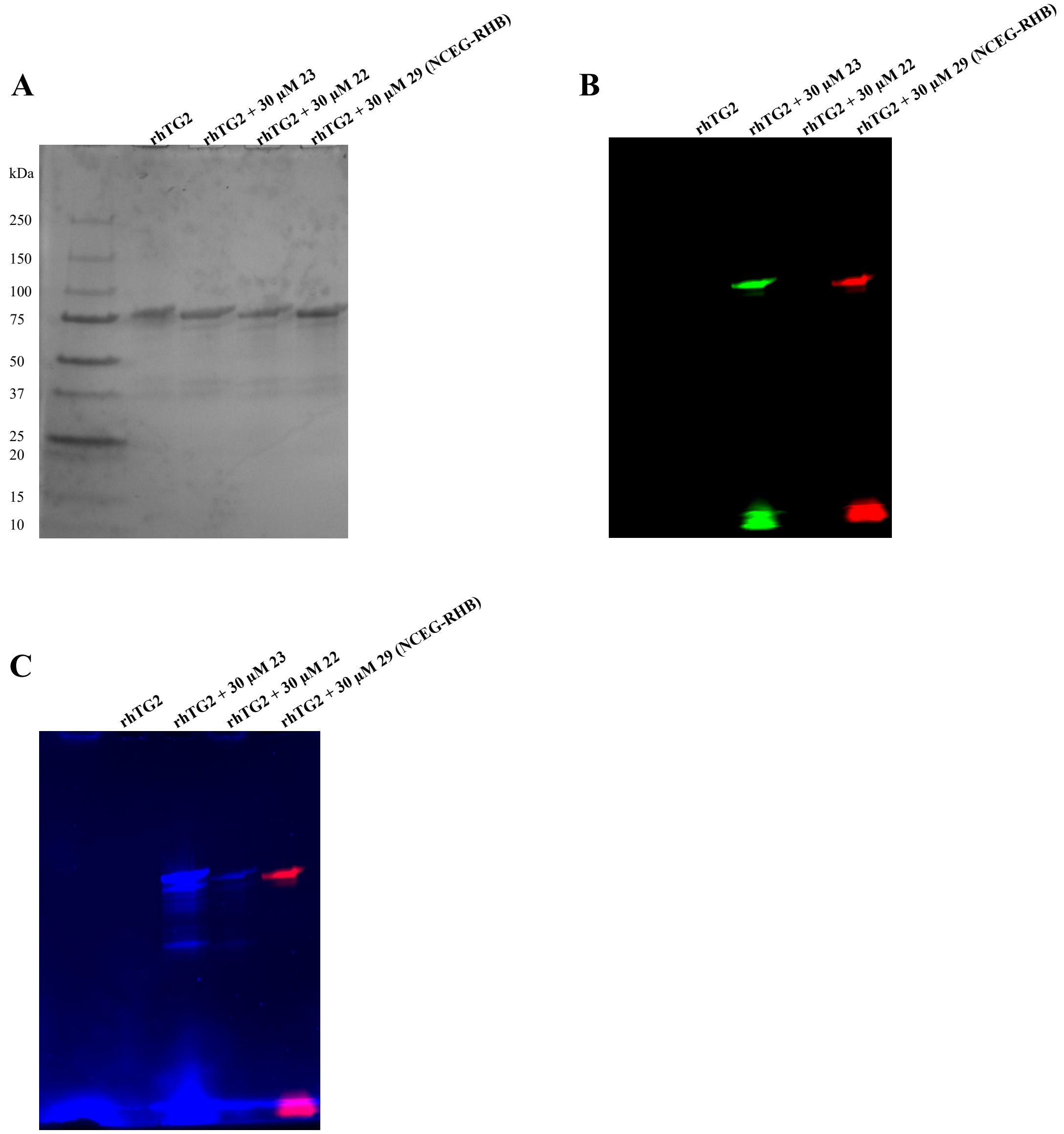


**Figure S9.** **A)** Coomassie stain of full SDS-PAGE used for fluorescent labelling of purified rhTG2. **B)** Multi-channel blue epi illumination with 530/28 nm filter (green band colour) and green epi illumination with 605/50 nm filter (red band colour) of full SDS-PAGE used for fluorescent labelling of purified rhTG2. **C)** Multi-channel UV trans illumination with 530/28 nm filter (blue band colour) and green epi illumination with 605/50 nm filter (red band colour) of full SDS-PAGE used for fluorescent labelling of purified rhTG2.

## Supplementary cellular evaluation figures

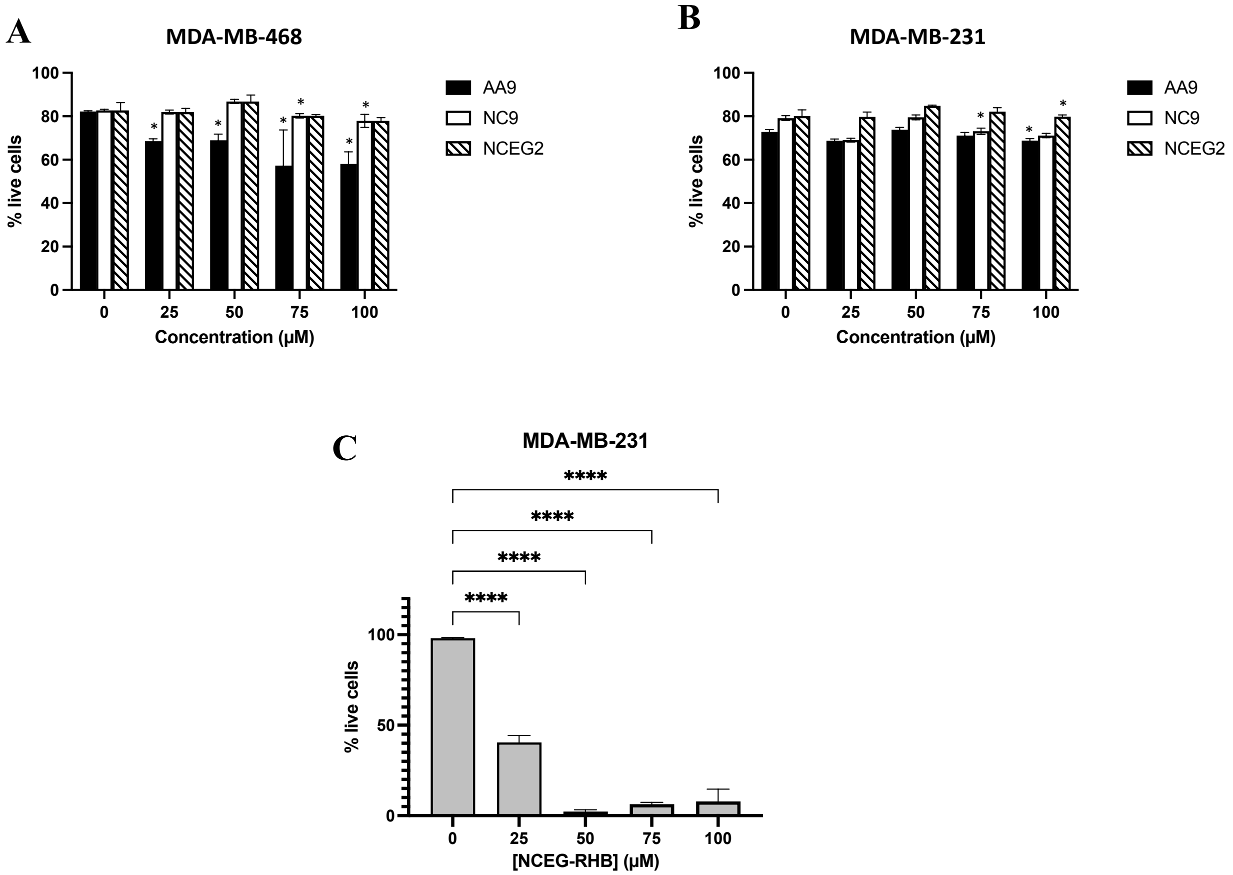


**Figure S10.** Viability assay: Treatment of (**A**) MDA-MB-468 breast cancer cell line with cell impermeable inhibitor **18 (NCEG2)** and cell permeable inhibitors NC9 and AA9, employed at the indicated concentrations and (**B)** MDA-MB-231 breast cancer cell line with cell impermeable inhibitor **18 (NCEG2)** and cell permeable inhibitors NC9, AA9, and **(C) NCEG-RHB** employed at the indicated concentrations. Experiments were carried out in triplicate and are represented as average ± SD of the percentage of living cells; (*) *P* value < 0.05; (****) *P* value < 0.0001.

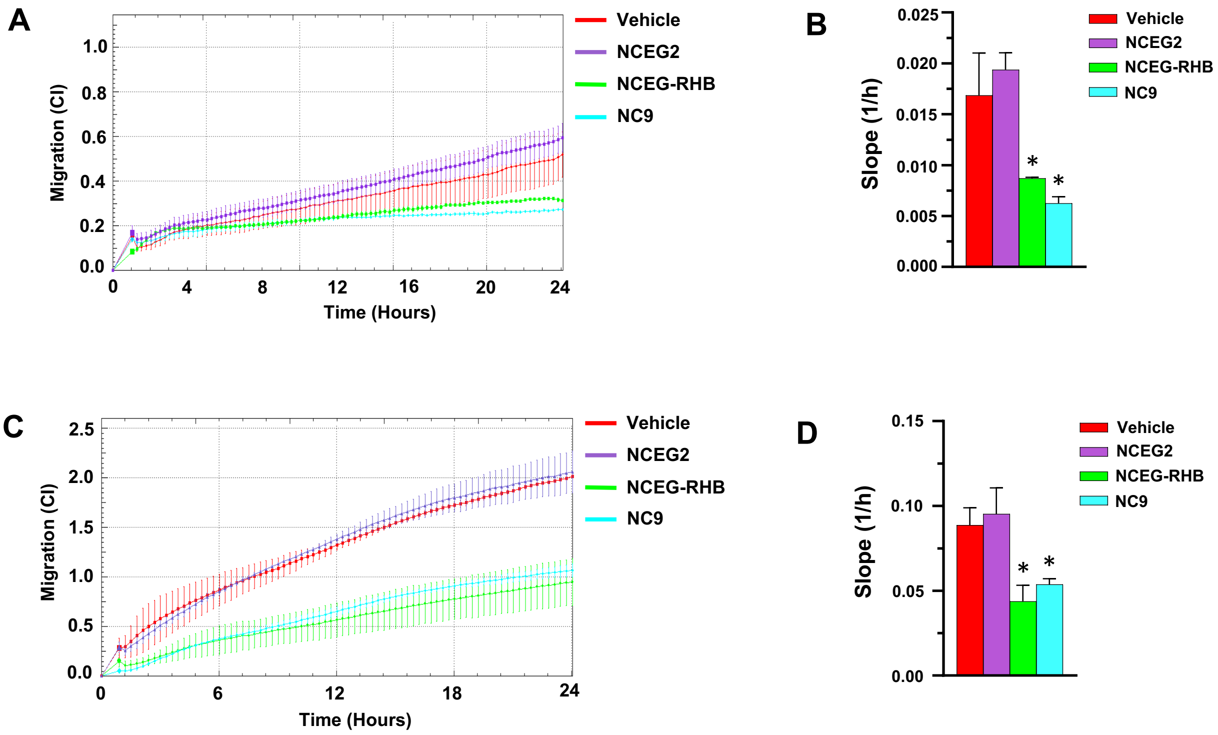


**Figure S11.** Migration assay: **A)** Dynamic monitoring of the migration of MDA-MB-436 cells. We recorded for 24 hours the migration of the cells in the presence only of the vehicle or of the indicated TG2 inhibitors, used at 25 μM concentrations. Data are reported as Cell Index (CI) values ± SD. **B)** Slope analysis shown at 24 h comprising the steepness, inclination, gradient, and changing rate of the CI curves of MDA-MB-436 cells over the time. * $P < 0.05$ *versus* vehicle. **C)** Dynamic monitoring of the migration of MDA-MB-231 cells. We recorded for 24 hours the migration of the cells in the presence only of the vehicle or of the indicated TG2 inhibitors, used at 25 μM concentrations. Data are reported as Cell Index (CI) values ± SD. **D)** Slope analysis shown at 24 h comprising the steepness, inclination, gradient, and changing rate of the CI curves of MDA-MB-231 cells over the time. * $P < 0.05$ *versus* vehicle.

## Biotinylated inhibitor published by Hauser *et al.*

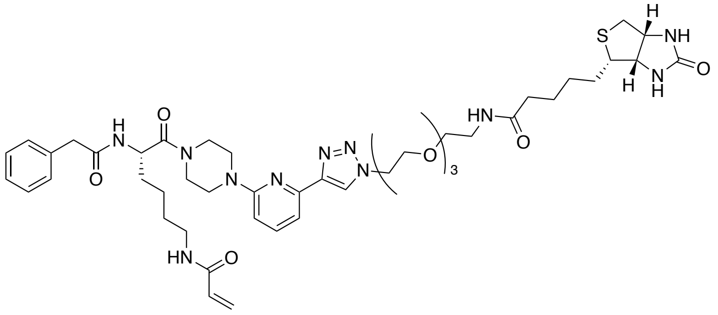

**Hauser's Biotinylated Inhibitor**

**Figure S12.** Structure of biotinylated TG2 inhibitor recently published by Hauser *et al.* [53].

## SDS-PAGE of pull-down from *E. coli*

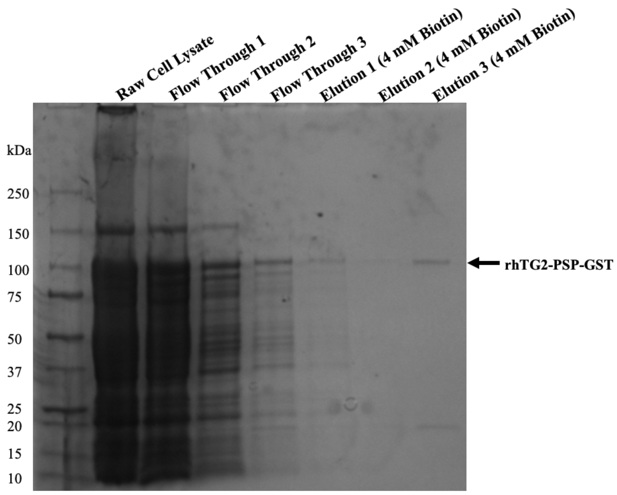


**Figure S13.** SDS-PAGE of pull-down experiment of recombinant human TG2 from *E. coli* cell lysate visualized with Coomassie staining.

## Synthesis and characterization of intermediates and final compounds

## Experimental Section

Where available, commercially available reagents and solvents were purchased from suppliers including Sigma-Aldrich, Oakwood Products, Combi-Blocks, and Fisher Scientific and used without further purification. Solid phase peptide synthesis resin was purchased from MilliporeSigma. Thin layer chromatography (TLC) was performed using SiliCycle aluminium backed TLC plates 200-µm thickness with F-254 indicator and visualized using short wave UV light. Preparatory-TLC was executed on SiliCycle SiliaPlate glass backed TLC with extra hard layer 60Å 250 µm thickness plates and F-254 indicator. Flash chromatography purification was performed using 230-400 mesh silica gel. All $^{1}H$- and $^{13}C$-NMR spectra were referenced to the indicated deuterated solvent and acquired using a Bruker 400-MHz or 600-MHz instrument to report the peaks in ppm. The high-resolution mass spectra were obtained using an electrospray ionization source (ESI) and quadrupole time-of-flight (QTOF) analyser. All final compounds were further evaluated for purity by HPLC where indicated.

### *General synthetic procedure A*

To a round bottom flask equipped with magnetic stir bar was added the acid (1 eq) solubilized in a 1:1 mixture of THF:$H_2O$ (0.1 M). To the flask was added finely ground potassium hydroxide (4 eq). The reaction mixture was then stirred for 2 h. Upon completion, the reaction mixture was diluted with $H_2O$ and washed once with ether. The aqueous phase was then acidified to pH 2 with 1 M HCl. The aqueous layer was then extracted three times with ethyl acetate. The organic layers were combined, washed once with brine, dried over $MgSO_4$, filtered, and concentrated under reduced pressure to yield the product without further purification.

### *General synthetic procedure B*

To a round bottom flask equipped with magnetic stir bar was added the acid (1.5 eq), HATU (1.5 eq), and DIPEA (3 eq) solubilized in DMF (0.5 M) at 0 ˚C. After 0.5 h, the amine (1 eq) was added dropwise as a solution in DMF (0.5 M) with DIPEA (1 eq). The ice bath was removed, and the reaction was allowed to stir overnight at room temperature. Upon completion, the reaction mixture was quenched with $H_2O$ and extracted once with ethyl acetate. The organic layer was then washed with 1 M HCl, $H_2O$, sat. $NaHCO_3$ solution, and brine. The organic phase was then dried over $MgSO_4$, filtered, and concentrated under reduced pressure. The resulting crude oil was then purified *via* flash chromatography to obtain the product.

### *SPPS Wang resin loading procedure*

To pre-load the first residue onto Wang resin, a procedure using a symmetrical anhydride was adapted from Atherton *et al.* [55]. To a dry SPPS reaction vessel was added 1 gram of Wang resin. The resin was suspended in 15 mL dry DMF under inert atmosphere and allowed to swell for 30 min. To form the corresponding anhydride to load onto the resin, 4.937 g Fmoc-Asp(OtBu)-OH (12 mmol, 10 eq) was solubilized in 120 mL dry DCM in a flame-dried round-bottom flask equipped with magnetic stir bar under inert atmosphere. To the flask was added 0.929 mL DIC (6 mmol, 5 eq) and the solution was stirred for 20 min at 0 ˚C. The volatiles were then removed from the round bottom flask under reduced pressure the residue was re-solubilized in a minimum amount of DMF. The solution was then added along with 0.015 g DMAP (1.2 mmol, 0.1 eq) to the SPPS reaction vessel, which was subsequently sealed and occasionally stirred for 1 h. Upon

completion, the resin was washed with DMF and DCM. The resin was then dried under reduced pressure and the loading was quantified according to the procedure published by Gude *et al.* [56]. The resin was finally capped using 0.319 mL acetic anhydride (6 mmol, 5 eq), 1.176 mL DIPEA (12 mmol, 10 eq) in 15 mL DCM for 15 min. The resin was once again washed and dried under reduced pressure.

***General synthetic procedure C***

Wang resin was preloaded with Fmoc-Asp(OtBu)-OH offline using the resin loading protocol described above. Resin was loaded into a CEM Liberty Blue Peptide Synthesizer, and the cell impermeable inhibitors were generated using 2 min couplings at 90 ˚C (0.2 M amino acid (5 eq) in DMF, 1.0 M DIC (10 eq) in DMF, and 1.0 M Oxyma Pure (5 eq) in DMF and 1 min Fmoc deprotections at 90˚C. The deprotection solution contained 20% Piperidine in DMF with 0.1 M Oxyma Pure to prevent aspartimide formation. Upon completion, the peptidic inhibitors were cleaved from resin using a cleavage cocktail of 95% TFA, 2.5% $H_2O$, and 2.5% TIPS for 4 h. The crude peptide was precipitated using cold ether, collected by centrifugation, and purified by semi-preparative HPLC.

***Synthesis of compound 2***

To a round bottom flask equipped with magnetic stir bar was added 5.000 g of commercially available Z-Glu(OtBu)-OH **1** (14.821 mmol, 1 eq) solubilized in 30 mL DMF with 2.656 g cesium carbonate (8.151 mmol, 0.55 eq) and stirred for 1 h. After which 0.923 mL iodomethane (14.821 mmol, 1 eq) was added and the reaction mixture was stirred overnight. Upon completion, the reaction mixture was diluted with ethyl acetate and washed sequentially with 1 M HCl, $H_2O$, sat. $NaHCO_3$ solution, and brine. The organic layer was dried over $MgSO_4$, filtered, and concentrated under reduced pressure to yield 5.04 g (97%) of the product as a white solid.

$^1$H NMR (400 MHz, $CDCl_3$) δ 7.39 – 7.28 (m, 5H), 5.46 (d, *J* = 8.2 Hz, 1H), 5.09 (s, 2H), 4.38 (td, *J* = 8.3, 5.0 Hz, 1H), 3.73 (s, 3H), 2.40 – 2.21 (m, 2H), 2.20 – 2.07 (m, 1H), 2.00 – 1.86 (m, 1H), 1.42 (s, 9H).

$^{13}$C NMR (101 MHz, $CDCl_3$) δ 172.55, 172.02, 156.02, 136.33, 128.59, 128.24, 128.17, 80.87, 67.08, 53.54, 52.53, 31.47, 28.13, 27.67.

HRMS (ESI-QTOF) m/z [M + Na]+ calcd for $C_{18}H_{25}NO_6Na$ 374.1580; found 374.1583.

***Synthesis of compound 3***

To a round bottom flask under inert atmosphere was added 5.04 g compound **2** (14.451 mmol, 1 eq) solubilized in 143 mL methanol. Palladium on carbon (0.305 g, 2.869 mmol, 20 mol%) was then added to the flask which was subsequently evacuated and backfilled three times with $N_{2\,(g)}$. The flask was then evacuated and backfilled three times with $H_{2\,(g)}$ and stirred overnight. Upon completion, the mixture was evacuated and backfilled three times with $N_{2\,(g)}$ and filtered over a pad a celite. The filtrate was then concentrated under reduced pressure to yield the product as a clear oil in quantitative yield (3.057 g) and carried forward without further purification.

$^{1}$H NMR (400 MHz, MeOD) δ 3.79 (s, 3H), 2.50 – 2.30 (m, 2H), 2.13 – 1.90 (m, 2H), 1.45 (s, 9H).

$^{13}$C NMR (101 MHz, MeOD) δ 173.71, 173.44, 81.97, 53.85, 53.12, 31.98, 28.77, 28.32.

HRMS (ESI-QTOF) m/z [M + Na]+ calcd for $C_{10}H_{19}NO_4Na$ 240.1212; found 240.1216.

***Synthesis of compound 5***

To a round bottom flask equipped with magnetic stir bar was added 3.057 g compound **3** (14.343 mmol, 1 eq) solubilized in 143 mL DCM with 2.199 mL triethylamine (15.778 mmol, 1.2 eq). To the flask was added 2.734 g 1-naphthoyl chloride **4** (14.343 mmol, 1.1 eq) dropwise and the reaction was stirred. After five minutes 0.175 g DMAP (1.434 mmol, 10 mol%) was added and the reaction mixture was stirred for 2 h. Upon completion the reaction mixture was washed sequentially with 1 M HCl, $H_2O$, sat. $NaHCO_3$ solution, and brine. The organic layer was then dried over $MgSO_4$, filtered, and concentrated under reduced pressure. The resulting crude oil was purified *via* flash chromatography to yield 4.47 g (84%) of the product as a white solid.

$^{1}$H NMR (400 MHz, $CDCl_3$) δ 8.41 – 8.33 (m, 1H), 7.92 (dt, *J* = 8.3, 1.2 Hz, 1H), 7.88 – 7.84 (m, 1H), 7.67 (dd, *J* = 7.1, 1.2 Hz, 1H), 7.59 – 7.49 (m, 2H), 7.45 (dd, *J* = 8.3, 7.0 Hz, 1H), 6.78 (d, *J* = 7.9 Hz, 1H), 3.81 (s, 3H), 2.54 – 2.24 (m, 3H), 2.18 – 2.01 (m, 1H), 1.41 (s, 9H).

$^{13}$C NMR (101 MHz, $CDCl_3$) δ 172.56, 172.28, 169.46, 133.80, 133.71, 131.09, 130.31, 128.39, 127.36, 126.57, 125.55, 125.39, 124.76, 81.13, 52.74, 52.36, 31.71, 28.14, 27.39.

HRMS (ESI-QTOF) m/z [M + Na]+ calcd for $C_{21}H_{25}NO_5Na$ 394.1630; found 394.1641.

***Synthesis of compound 6***

Compound **6** was synthesized according to general synthetic procedure A. The product was obtained as a white solid (4.30 g, 99%).

$^{1}$H NMR (400 MHz, $CDCl_3$) δ 8.39 – 8.29 (m, 1H), 7.89 (dt, *J* = 8.2, 1.0 Hz, 1H), 7.86 – 7.80 (m, 1H), 7.63 (dd, *J* = 7.1, 1.3 Hz, 1H), 7.57 – 7.46 (m, 2H), 7.40 (dd, *J* = 8.3, 7.0 Hz, 1H), 7.09 (d, *J* = 7.4 Hz, 1H), 4.85 (td, *J* = 7.8, 5.0 Hz, 1H), 2.57 – 2.25 (m, 3H), 2.18 – 2.06 (m, 1H), 1.40 (s, 9H).

$^{13}$C NMR (101 MHz, $CDCl_3$) δ 175.21, 173.05, 170.30, 133.74, 133.16, 131.26, 130.24, 128.39, 127.42, 126.59, 125.62, 125.46, 124.71, 81.56, 52.64, 31.89, 28.10, 26.93.

HRMS (ESI-QTOF) m/z [M + Na]+ calcd for $C_{20}H_{23}NO_5Na$ 380.1474; found 380.1468.

***Synthesis of compound 8***

To a round bottom flask equipped with magnetic stir bar was added 2.000 g of commercially available Z-Lys-OH **7** (7.13 mmol, 1 eq) suspended in 50 mL MeOH under nitrogen atmosphere. The suspension was cooled to 0 ˚C and 0.78 mL thionyl chloride (10.7 mmol, 1.5 eq) was added

dropwise. The reaction mixture was allowed to warm to room temperature and was stirred overnight. Upon completion, the volatiles were removed under reduced pressure to yield the product in quantitative yield (2.35 g) as a white solid and was carried forward without further purification or characterization.

***Synthesis of compound 10***

To a round bottom flask equipped with magnetic stir bar was added 4.348 g compound **8** (13.144 mmol, 1 eq) solubilized in 40 mL ACN with 6.868 mL DIPEA (39.431 mmol, 3 eq) and 0.161 g DMAP (1.314 mmol, 10 mol%) at 0 ˚C. Acryloyl chloride **9** (1.267 mL, 15.772 mmol, 1.2 eq) was solubilized in 12 mL ACN and added dropwise to the reaction mixture *via* dropping funnel. The reaction was stirred for 1 h after which the reaction mixture was concentrated under reduced pressure and the resulting oil was taken up in ethyl acetate. The organic mixture was washed once with 1 M HCl, $H_2O$, sat. $NaHCO_3$ solution, and brine. The organic layer was then dried over $MgSO_4$, filtered, and concentrated under reduced pressure. The resulting crude oil was purified *via* flash chromatography to yield 2.65 g of the product (58%) as a white solid.

$^1$H NMR (400 MHz, $CDCl_3$) δ 7.29 (d, *J* = 5.0 Hz, 5H), 6.58 – 6.47 (m, 1H), 6.20 (dd, *J* = 17.0, 1.8 Hz, 1H), 6.07 (dd, *J* = 17.0, 10.1 Hz, 1H), 5.75 (d, *J* = 8.1 Hz, 1H), 5.51 (dd, *J* = 10.0, 1.8 Hz, 1H), 5.04 (s, 2H), 4.26 (td, *J* = 8.2, 4.7 Hz, 1H), 3.66 (s, 3H), 3.23 (qt, *J* = 10.6, 5.6 Hz, 2H), 1.84 – 1.58 (m, 2H), 1.57 – 1.40 (m, 2H), 1.40 – 1.25 (m, 2H).

$^{13}$C NMR (101 MHz, $CDCl_3$) δ 172.91, 165.85, 156.18, 136.18, 130.89, 128.44, 128.10, 127.95, 126.08, 66.84, 53.66, 52.30, 38.84, 31.79, 28.73, 22.44.

***Synthesis of compound 11***

Compound **11** was synthesized according to general synthetic procedure A. The product was obtained as a white solid (7.807 g, quantitative yield).

$^1$H NMR (400 MHz, $CDCl_3$) δ 7.36 – 7.27 (m, 5H), 6.30 – 6.19 (m, 2H), 6.08 (dd, *J* = 17.0, 10.3 Hz, 1H), 5.75 (d, *J* = 8.0 Hz, 1H), 5.60 (dd, *J* = 10.3, 1.4 Hz, 1H), 5.08 (d, *J* = 1.8 Hz, 2H), 4.35 (q, *J* = 7.3 Hz, 1H), 3.29 (d, *J* = 2.9 Hz, 2H), 1.95 – 1.67 (m, 2H), 1.60 – 1.30 (m, 4H).

$^{13}$C NMR (101 MHz, $CDCl_3$) δ 174.91, 166.49, 156.40, 136.18, 130.43, 128.54, 128.21, 128.06, 127.11, 67.06, 53.52, 39.22, 31.79, 28.73, 22.22.

HRMS (ESI-QTOF) m/z [M + Na]+ calcd for $C_{17}H_{22}N_2O_5Na$ 357.1426; found 357.1446.

***Synthesis of compound 13***

Compound **13** was synthesized according to general synthetic procedure B. The product was obtained after flash chromatography as a white solid (1.816 g, 98%).

$^1$H NMR (400 MHz, $CDCl_3$) δ 7.34 – 7.27 (m, 5H), 6.29 (t, *J* = 5.7 Hz, 1H), 6.21 (dd, *J* = 17.0, 1.7 Hz, 1H), 6.06 (dd, *J* = 17.0, 10.2 Hz, 1H), 5.95 (d, *J* = 8.3 Hz, 1H), 5.54 (dd, *J* = 10.1, 1.7 Hz,

1H), 5.04 (s, 2H), 4.59 (td, $J$ = 8.2, 4.4 Hz, 1H), 3.66 – 3.21 (m, 10H), 1.70 – 1.61 (m, 1H), 1.60 – 1.46 (m, 3H), 1.43 (s, 9H), 1.40 – 1.30 (m, 2H).

$^{13}$C NMR (101 MHz, $CDCl_3$) δ 170.47, 165.76, 156.23, 154.42, 136.24, 130.93, 128.52, 128.18, 127.99, 126.15, 80.42, 66.91, 50.33, 45.36, 41.96, 38.96, 32.75, 28.80, 28.35, 22.26.

HRMS (ESI-QTOF) m/z [M + Na]+ calcd for $C_{26}H_{38}N_4O_6Na$ 525.2689; found 525.2674.

***Synthesis of compound 14***

To a round bottom flask equipped with magnetic stir bar was added 1.816 g compound **13** (3.613 mmol, 1 eq) solubilized in 36 mL of a 20% v/v TFA in DCM solution. The reaction mixture was stirred for 2 h upon which the solvent was removed under reduced pressure. The resulting oil was co-evaporated 3 times with DCM under reduced pressure to yield the product in quantitate yield (1.866 g) as a brown oil. The product was carried forward as the TFA salt without further purification or characterization.

***Synthesis of compound 15***

Compound **15** was synthesized according to general synthetic procedure B. The product was obtained after flash chromatography as a white solid (1.778 g, 66%)

$^{1}$H NMR (400 MHz, $CDCl_3$) δ 8.32 (d, $J$ = 7.9 Hz, 1H), 7.88 (d, $J$ = 8.3 Hz, 1H), 7.83 (dd, $J$ = 7.6, 1.8 Hz, 1H), 7.63 (ddd, $J$ = 7.1, 3.0, 1.2 Hz, 1H), 7.55 – 7.46 (m, 2H), 7.41 (dd, $J$ = 8.3, 7.0 Hz, 1H), 7.31 (d, $J$ = 3.4 Hz, 5H), 7.25 – 7.18 (m, 1H), 6.31 – 6.27 (m, 1H), 6.26 – 6.15 (m, 1H), 6.12 – 5.98 (m, 2H), 5.57 – 5.50 (m, 1H), 5.32 – 5.26 (m, 1H), 5.05 (s, 2H), 4.64 – 4.57 (m, 1H), 3.94 – 3.11 (m, 11H), 2.54 – 2.30 (m, 2H), 2.18 – 2.06 (m, 1H), 1.87 – 1.76 (m, 1H), 1.66 – 1.28 (m, 15H).

$^{13}$C NMR (101 MHz, $CDCl_3$) δ 172.08, 170.60, 170.24, 169.20, 165.63, 156.20, 136.26, 133.64, 130.90, 130.14, 128.51, 128.32, 128.18, 128.00, 127.17, 126.41, 126.08, 125.28, 124.65, 80.89, 66.89, 50.31, 48.40, 45.04, 42.10, 38.85, 32.54, 30.56, 28.84, 28.09, 27.87, 22.24. (Rotamers of piperazine observed at 45.04 ppm and 42.10 ppm)

HRMS (ESI-QTOF) m/z [M + Na]+ calcd for $C_{41}H_{51}N_5O_8Na$ 764.3635; found 764.3645.

***Synthesis of compound 16***

To a round bottom flask equipped with magnetic stir bar was added 0.100 g compound **15** (0.135 mmol, 1 eq) solubilized in 1.5 mL of a 20% v/v TFA in DCM solution. The reaction mixture was stirred for 4 h upon which the solvent was removed under reduced pressure. The resulting brown oil was then precipitated using cold ether. The crude solid was further purified by flash chromatography to yield 0.039 g (42%) of product as a white solid.

$^{1}$H NMR (600 MHz, $CDCl_3$) δ 8.31 – 8.24 (m, 1H), 7.88 (t, $J$ = 8.1 Hz, 1H), 7.85 – 7.80 (m, 1H), 7.66 – 7.61 (m, 1H), 7.57 – 7.45 (m, 2H), 7.43 – 7.27 (m, 7H), 6.34 – 6.19 (m, 2H), 6.13 – 5.96

(m, 2H), 5.61 – 5.51 (m, 1H), 5.40 – 5.30 (m, 1H), 5.10 – 5.02 (m, 2H), 4.71 – 4.60 (m, 1H), 3.93 – 3.15 (m, 11H), 2.66 – 2.55 (m, 1H), 2.53 – 2.43 (m, 1H), 2.22 – 2.08 (m, 1H), 2.00 – 1.80 (m, 1H), 1.72 – 1.42 (m, 4H), 1.40 – 1.28 (m, 2H).

$^{13}$C NMR (151 MHz, $CDCl_3$) δ 175.18, 170.62, 169.64, 166.38, 166.11, 156.40, 136.30, 133.75, 133.34, 133.31, 131.22, 130.67, 130.18, 128.66, 128.48, 128.33, 128.14, 128.12, 127.40, 126.60, 125.64, 125.34, 124.78, 67.16, 67.15, 67.12, 67.10, 50.50, 48.82, 48.77, 48.60, 45.57, 42.15, 39.13, 32.64, 29.51, 28.83, 22.33.

HRMS (ESI-QTOF) m/z [M + Na]+ calcd for $C_{37}H_{43}N_5O_8Na$ 708.3009; found 708.2991.

***Synthesis of compound 17***

Compound **17** was synthesized according to general synthetic procedure C using Fmoc-Aminohexanoic acid-OH as linker on 0.1 mmol scale (3.4 mg isolated).

HRMS (ESI-QTOF) m/z [M – H]$^-$ calcd for $C_{55}H_{68}N_9O_{18}$ 1142.4682; found 1442.4698.

Purity: 95.5%

***Synthesis of compound 18 (NCEG2)***

Compound **18** was synthesized according to general synthetic procedure C using three glycine residues (iterative couplings and deprotections) as linker on 0.1 mmol scale (7.4 mg isolated).

HRMS (ESI-QTOF) m/z [M – H]$^-$ calcd for $C_{55}H_{66}N_{11}O_{20}$ 1200.4486; found 1200.4473.

Purity: 96.4%

***Synthesis of compound 19***

Compound **19** was synthesized according to general synthetic procedure B. The product was obtained after flash chromatography as a white solid (0.098 g, 63%).

HRMS (ESI-QTOF) m/z [M + Na]+ calcd for $C_{47}H_{63}N_7O_9Na$ 892.4585; found 892.4579.

***Synthesis of compound 20***

To a round bottom flask equipped with magnetic stir bar was added 0.319 g **19** (0.367 mmol, 1 eq) solubilized in 2 mL of a 1:1 mixture of 4 M HCl in dioxane and DCM. The reaction was stirred for 2 h after which the solvents were removed under reduced pressure. The product was carried forward as the HCl salt in quantitative yield (0.296 g) and without further purification or characterization.

***Synthesis of compound 22***

Compound **22** was synthesized according to general synthetic procedure B. The product was obtained after flash chromatography as a vibrant yellow solid (0.073 g, 59%).

HRMS (ESI-QTOF) m/z [M + Na]+ calcd for $C_{56}H_{68}N_8O_{10}Na$ 1035.4956; found 1035.4944.

Purity: 85.3%

***Synthesis of compound 23***

To a round bottom flask equipped with magnetic stir bar was added 0.099 g compound **20** (0.123 mmol, 1 eq) solubilized in 2 mL DMF with 0.021 mL DIPEA (0.123 mmol, 1 eq). To the flask was added 0.053 g fluorescein isothiocyanate isomer I (0.135 mmol, 1.1 eq) and 0.043 mL DIPEA (0.246 mmol, 2 eq). The reaction mixture was stirred for 48 h at room temperature. The volatiles were then evaporated by a stream of compressed air overnight. The resulting crude oil was purified *via* flash chromatography twice to yield 0.052 g (37%) of the product as a yellow solid.

HRMS (ESI-QTOF) m/z [M + Na]+ calcd for $C_{63}H_{66}N_8O_{12}SNa$ 1181.4419; found 1181.4425.

Purity: 89.9%

***Synthesis of compound 25***

Compound **25** was synthesized according to general synthetic procedure B. The product was obtained after flash chromatography as a colourless oil (0.470 g, 81%).

$^1$H NMR (400 MHz, $CDCl_3$) δ 7.31 – 7.21 (m, 5H), 5.14 – 4.94 (m, 2H), 4.26 – 4.19 (m, 1H), 3.56 – 3.31 (m, 2H), 3.19 – 2.90 (m, 4H), 2.25 – 2.01 (m, 1H), 1.94 – 1.72 (m, 3H), 1.45 – 1.16 (m, 15H).

$^{13}$C NMR (101 MHz, $CDCl_3$) δ 172.05, 155.98, 136.29, 128.39, 127.98, 127.69, 78.74, 67.08, 60.50, 46.92, 40.20, 39.02, 29.36, 28.98, 28.32, 23.73.

HRMS (ESI-QTOF) m/z [M + Na]+ calcd for $C_{23}H_{35}N_3O_5Na$ 456.2474; found 256.2462.

***Synthesis of compound 26***

To a round bottom flask under inert atmosphere was added 0.470 g compound **25** (1.084 mmol, 1 eq) solubilized in 11 mL methanol. Palladium on carbon (0.023 g, 0.217 mmol, 20 mol%) was then added to the flask which was subsequently evacuated and backfilled three times with $N_{2\,(g)}$. The flask was then evacuated and backfilled three times with $H_{2\,(g)}$ and stirred overnight. Upon completion, the mixture was evacuated and backfilled three times with $N_{2\,(g)}$ and filtered over a pad a celite. The filtrate was then concentrated under reduced pressure to yield the product as a clear oil in quantitative yield (0.325 g) and carried forward without further purification or characterization.

***Synthesis of compound 27***

Compound **27** was synthesized according to general synthetic procedure B excluding the 1 M HCl washes. The product was obtained after flash chromatography as a purple solid (0.663 g, 80%).

$^{1}$H NMR (400 MHz, $CDCl_3$) δ 7.77 – 7.48 (m, 3H), 7.35 – 7.27 (m, 2H), 7.24 – 7.14 (m, 1H), 6.93 – 6.85 (m, 1H), 6.82 – 6.74 (m, 2H), 6.67 (dd, *J* = 3.5, 2.4 Hz, 1H), 4.19 (dd, *J* = 7.7, 5.3 Hz, 1H), 3.65 – 3.46 (m, 10H), 3.32 – 2.87 (m, 5H), 2.11 – 1.14 (m, 31H).

$^{13}$C NMR (101 MHz, $CDCl_3$) δ 172.43, 170.79, 167.88, 167.48, 157.94, 155.64, 136.96, 136.48, 133.35, 132.37, 131.44, 130.61, 130.49, 130.29, 130.23, 130.16, 129.87, 129.80, 129.41, 127.92, 127.34, 115.78, 114.61, 114.13, 114.06, 113.89, 113.61, 113.35, 62.54, 60.17, 50.49, 46.16, 39.25, 32.51, 29.57, 29.05, 28.97, 28.55, 28.53, 28.28, 25.45, 24.02, 23.96, 22.19, 12.66.

HRMS (ESI-QTOF) m/z [M]+ calcd for $C_{43}H_{58}N_5O_5$ 724.4438; found 724.4414.

***Synthesis of compound 28***

To a round bottom flask equipped with magnetic stir bar was added 0.103 g **27** (0.135 mmol, 1 eq) solubilized in 1 mL of a 1:1 mixture of 4 M HCl in dioxane and DCM. The reaction was stirred for 2.5 h after which the solvents were removed under reduced pressure. The product was co-evaporated twice with DCM and then carried forward as the HCl salt in quantitative yield (0.094 g) and without further purification or characterization.

***Synthesis of compound 29***

Compound **29** was synthesized according to general synthetic procedure B excluding the 1 M HCl washes. The product was obtained after flash chromatography as a purple solid (0.035 g, 20%).

HRMS (ESI-QTOF) m/z [M]+ calcd for $C_{75}H_{91}N_{10}O_{10}$ 1291.6920; found 1291.6892.

Purity: 91.9%

***Synthesis of compound 31***

Compound **31** was synthesized according to general synthetic procedure B. The product was obtained after flash chromatography as a white solid (0.103 g, 91%).

$^{1}$H NMR (600 MHz, $CDCl_3$) δ 8.35 – 8.28 (m, 1H), 7.95 – 7.90 (m, 1H), 7.86 (d, *J* = 8.1 Hz, 1H), 7.68 – 7.63 (m, 1H), 7.58 – 7.49 (m, 2H), 7.45 – 7.30 (m, 6H), 6.27 – 6.17 (m, 1H), 6.16 – 6.01 (m, 2H), 5.95 – 5.81 (m, 1H), 5.61 – 5.53 (m, 1H), 5.23 – 5.13 (m, 1H), 5.09 – 5.02 (m, 2H), 4.66 – 4.57 (m, 1H), 4.17 – 4.06 (m, 1H), 4.06 – 3.94 (m, 1H), 3.87 – 3.12 (m, 10H), 2.54 – 2.43 (m, 1H), 2.42 – 2.34 (m, 1H), 2.25 – 2.14 (m, 2H), 1.97 – 1.86 (m, 1H), 1.72 – 1.44 (m, 4H), 1.42 – 1.31 (m, 2H).

$^{13}C$ NMR (151 MHz, $CDCl_3$) δ 172.00, 170.67, 170.00, 169.90, 165.78, 156.25, 136.31, 136.29, 133.80, 133.79, 131.38, 130.94, 130.15, 130.13, 128.74, 128.63, 128.55, 128.31, 128.29, 128.14, 128.11, 128.09, 127.46, 127.44, 127.41, 126.62, 126.41, 126.40, 125.68, 125.65, 125.22, 125.20, 124.76, 80.05, 71.30, 67.06, 50.36, 50.34, 50.25, 49.03, 49.01, 48.99, 48.94, 45.60, 45.54, 45.16, 45.11, 42.05, 41.79, 41.73, 39.04, 39.00, 38.97, 32.74, 32.72, 32.68, 32.65, 32.03, 32.01, 29.29, 29.27, 29.24, 29.19, 29.18, 29.00, 28.93, 28.90, 22.38, 22.29, 22.27.

HRMS (ESI-QTOF) m/z [M + Na]+ calcd for $C_{40}H_{46}N_6O_7Na$ 745.3326; found 745.3307.

Purity: 94.7%

***Synthesis of compound 32***

Compound **32** was synthesized according to general synthetic procedure B using commercially available desthiobiotin and amine-peg3-azide. The product was obtained after flash chromatography as an orange oil (0.320 g, >99%).

$^1H$ NMR (400 MHz, $CDCl_3$) δ 6.62 (t, $J$ = 5.6 Hz, 1H), 5.91 (s, 1H), 5.16 (s, 1H), 3.86 – 3.75 (m, 1H), 3.73 – 3.57 (m, 11H), 3.54 (dd, $J$ = 5.6, 4.6 Hz, 2H), 3.45 – 3.33 (m, 4H), 2.17 (t, $J$ = 7.4 Hz, 2H), 1.63 (p, $J$ = 7.1 Hz, 2H), 1.53 – 1.19 (m, 8H), 1.09 (d, $J$ = 6.5 Hz, 3H).

$^{13}C$ NMR (101 MHz, $CDCl_3$) δ 173.33, 164.08, 70.73, 70.58, 70.22, 70.11, 70.01, 56.19, 51.54, 50.73, 39.23, 35.97, 29.51, 28.63, 25.92, 25.26, 15.80.

HRMS (ESI-QTOF) m/z [M + Na]+ calcd for $C_{18}H_{34}N_6O_5Na$ 437.2488; found 437.2462.

***Synthesis of compound 33***

To a round bottom flask equipped with magnetic stir bar was added a slurry of 0.038 g alkyne **31** (0.053 mmol, 1 eq), desthiobiotin-peg3-azide **32** (0.053 mmol, 1 eq), and copper (II) sulfate pentahydrate (0.053 mmol, 1 eq) in 1 mL 1:1 ACN:$H_2O$. To the flask was then added 0.010 g sodium ascorbate (0.053 mmol, 1 eq) and the reaction was stirred at room temperature for 3 h. Upon completion the reaction mixture was filtered and subsequently concentrated under reduced pressure. The resulting crude oil was purified *via* preparatory-TLC to obtain the product as a clear oil (0.054 g, 90%)

HRMS (ESI-QTOF) m/z [M + Na]+ calcd for $C_{58}H_{80}N_{12}O_{12}Na$ 1159.5916; found 1159.5928.

Purity: 92.4%

# NMR spectra of intermediates and final compounds

## *Compound 2*

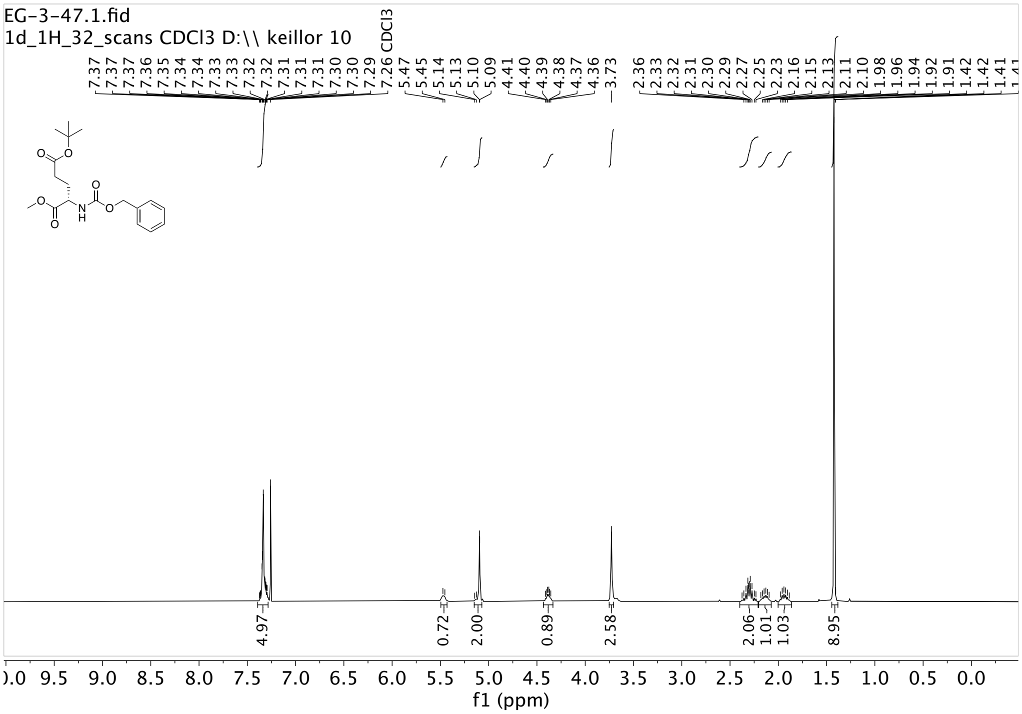


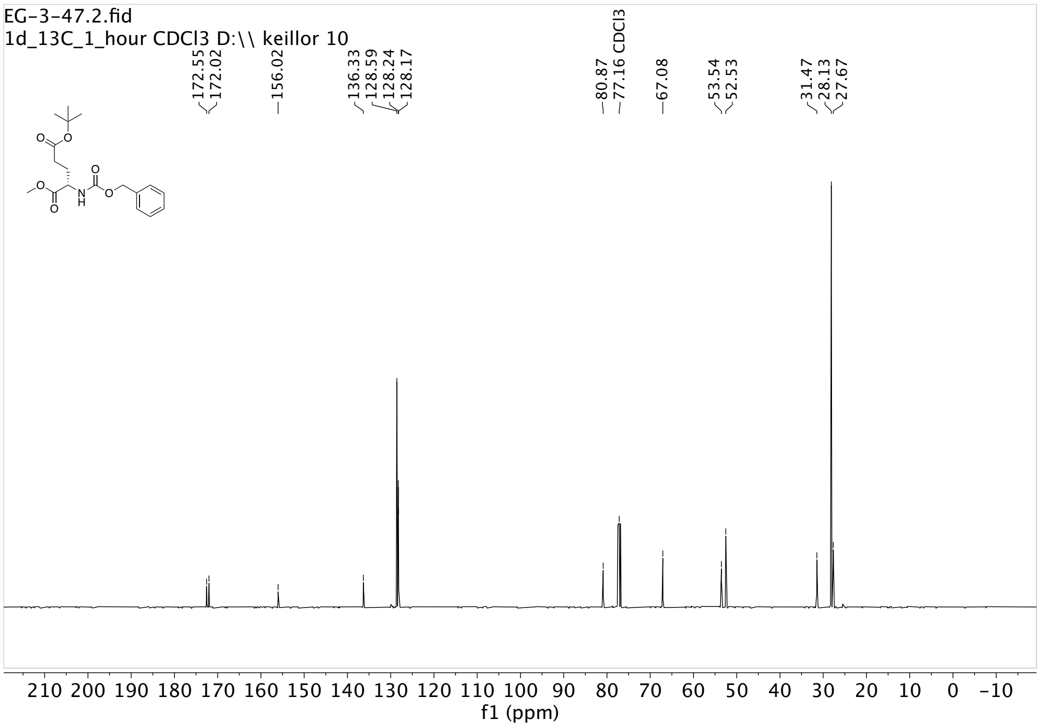

*Compound 3*

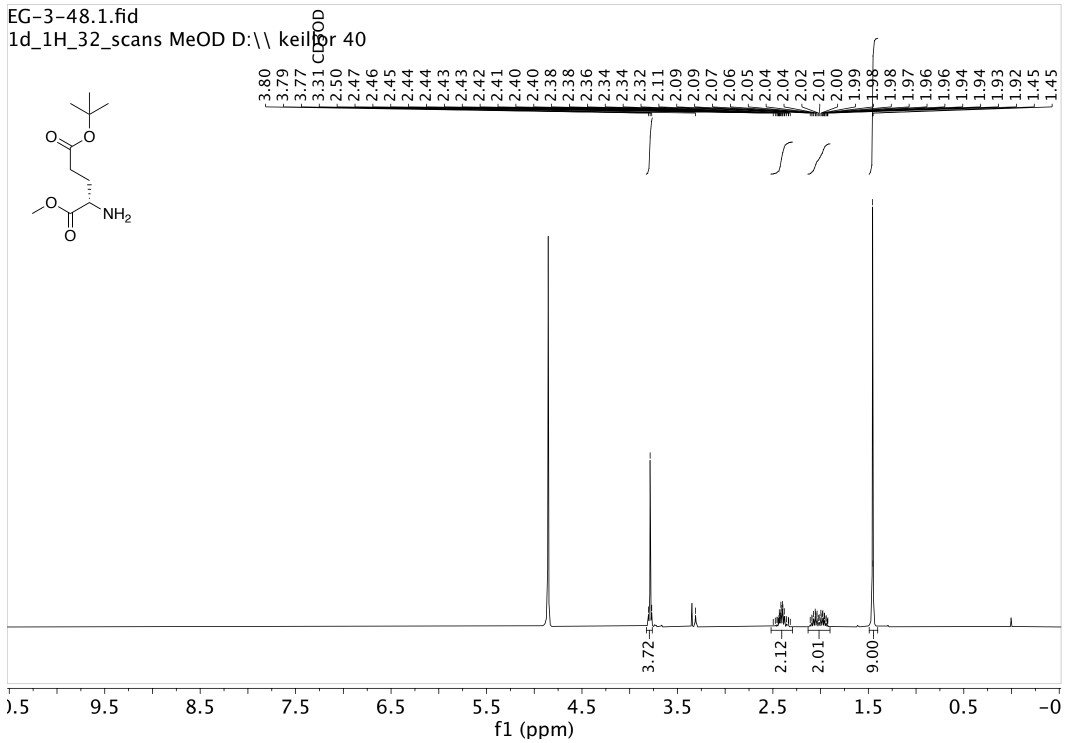
EG-3-48.1.fid
1d_1H_32_scans MeOD D:\\ keillor 40
3.80
3.79
3.77
3.31 CD3OD
2.50
2.47
2.46
2.45
2.44
2.44
2.43
2.43
2.42
2.41
2.40
2.40
2.38
2.38
2.36
2.34
2.34
2.32
2.11
2.09
2.09
2.07
2.06
2.05
2.04
2.04
2.02
2.01
2.00
1.99
1.98
1.98
1.97
1.96
1.96
1.94
1.94
1.93
1.92
1.45
1.45
O
O
O
O
NH2
O
3.72
2.12
2.01
9.00
f1 (ppm)

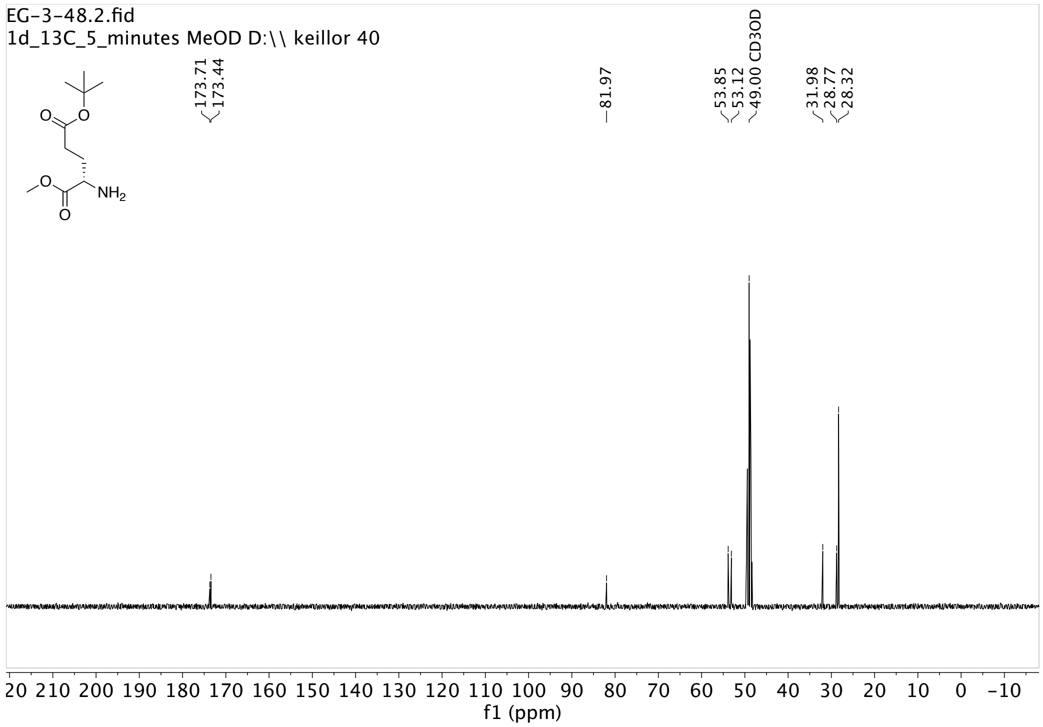
EG-3-48.2.fid
1d_13C_5_minutes MeOD D:\\ keillor 40
173.71
173.44
81.97
53.85
53.12
49.00 CD3OD
31.98
28.77
28.32
O
O
O
O
NH2
O
20 210 200 190 180 170 160 150 140 130 120 110 100 90 80 70 60 50 40 30 20 10 0 −10
f1 (ppm)

***Compound 5***

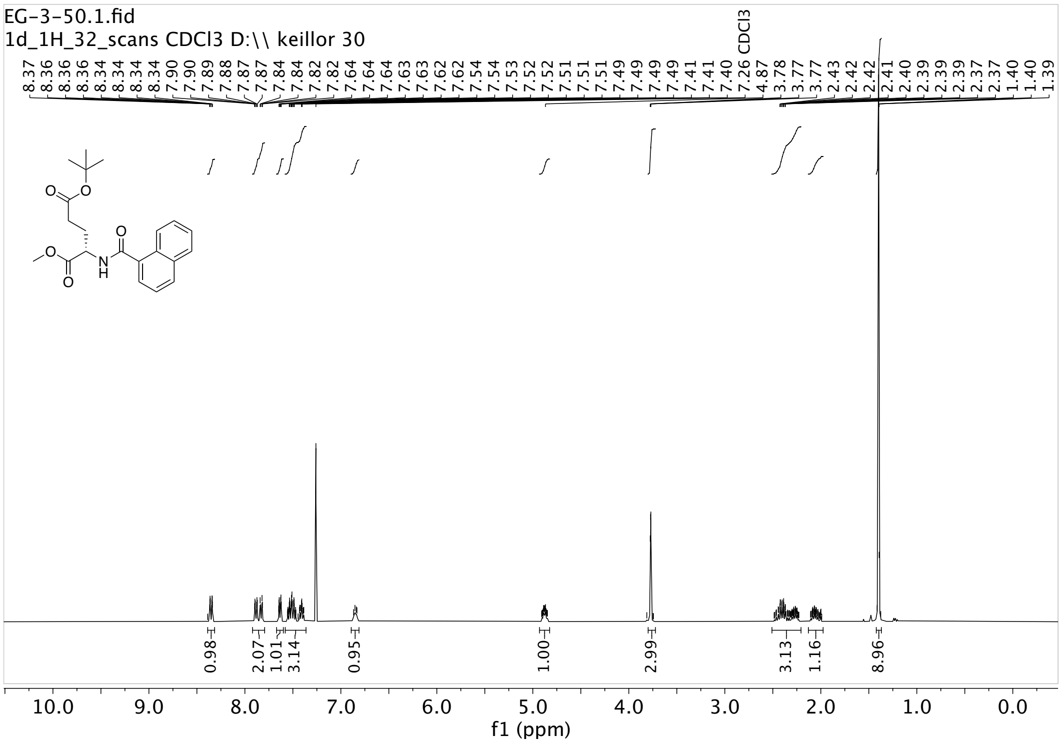

EG-3-50.1.fid
1d_1H_32_scans CDCl3 D:\\ keillor 30
8.37 8.36 8.36 8.36 8.34 8.34 8.34 8.34 7.90 7.89 7.88 7.87 7.87 7.84 7.84 7.82 7.82 7.64 7.64 7.63 7.63 7.62 7.54 7.54 7.53 7.52 7.52 7.51 7.51 7.51 7.49 7.49 7.49 7.41 7.41 7.40 7.26 CDCl3 4.87 3.78 3.77 3.77 2.43 2.42 2.42 2.41 2.40 2.39 2.39 2.39 2.37 2.37 1.40 1.40 1.39
0.98 2.07 1.01 3.14 0.95 1.00 2.99 3.13 1.16 8.96
10.0 9.0 8.0 7.0 6.0 5.0 4.0 3.0 2.0 1.0 0.0
f1 (ppm)


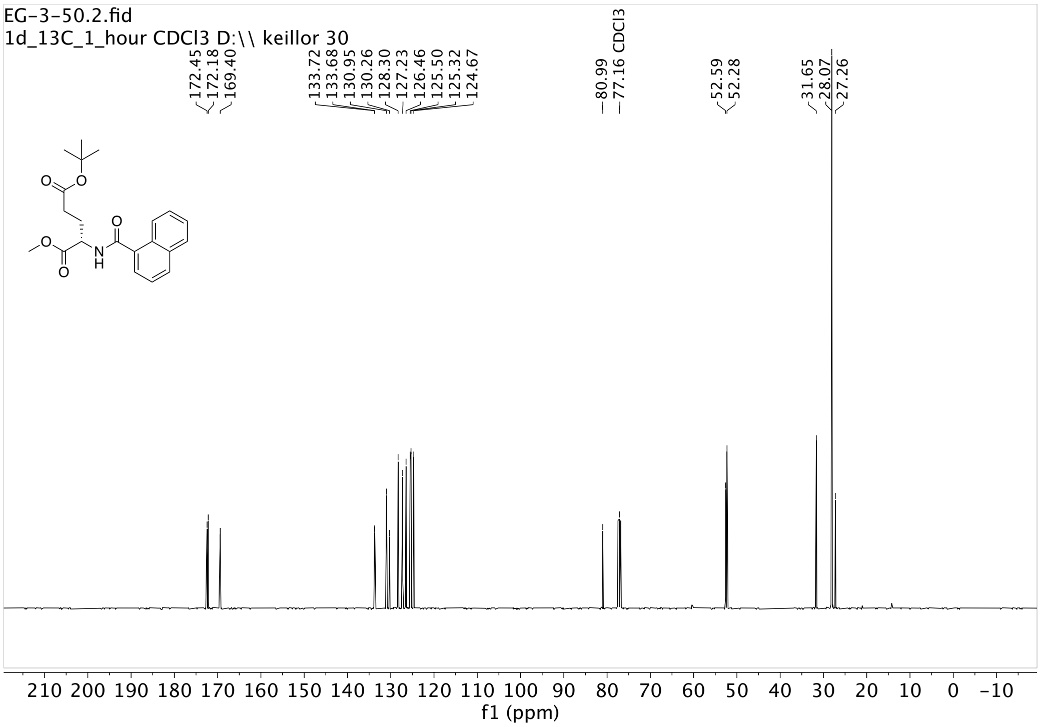

EG-3-50.2.fid
1d_13C_1_hour CDCl3 D:\\ keillor 30
172.45 172.18 169.40 133.72 133.68 130.95 130.26 128.30 127.23 126.46 125.50 125.32 124.67 80.99 77.16 CDCl3 52.59 52.28 31.65 28.07 27.26
210 200 190 180 170 160 150 140 130 120 110 100 90 80 70 60 50 40 30 20 10 0 -10
f1 (ppm)

## *Compound 6*

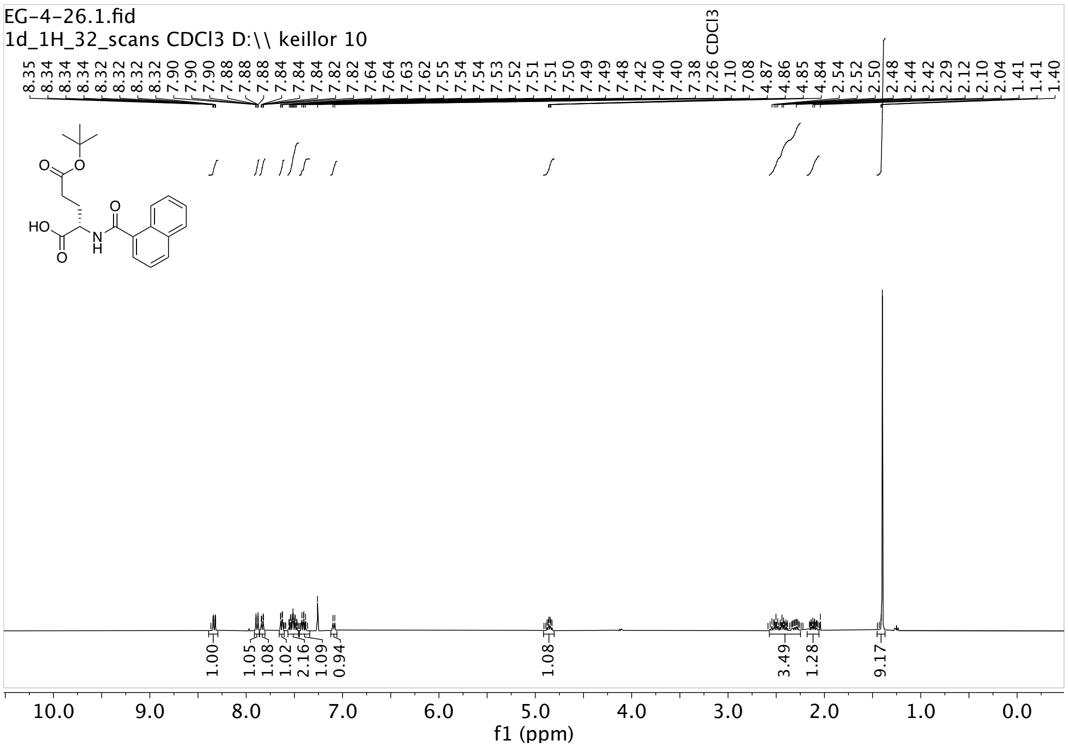

EG-4-26.1.fid
1d_1H_32_scans CDCl3 D:\\ keillor 10
8.35 8.34 8.34 8.34 8.32 8.32 8.32 8.32 7.90 7.90 7.88 7.88 7.88 7.84 7.84 7.84 7.82 7.82 7.64 7.64 7.63 7.62 7.55 7.54 7.54 7.54 7.53 7.52 7.51 7.51 7.50 7.49 7.49 7.48 7.42 7.40 7.40 7.38 7.26 CDCl3 7.10 7.08 4.87 4.86 4.85 4.84 2.54 2.52 2.50 2.48 2.44 2.42 2.29 2.12 2.10 2.04 1.41 1.41 1.40
1.00 1.05 1.08 1.02 2.16 1.09 0.94 1.08 3.49 1.28 9.17
10.0 9.0 8.0 7.0 6.0 5.0 4.0 3.0 2.0 1.0 0.0
f1 (ppm)


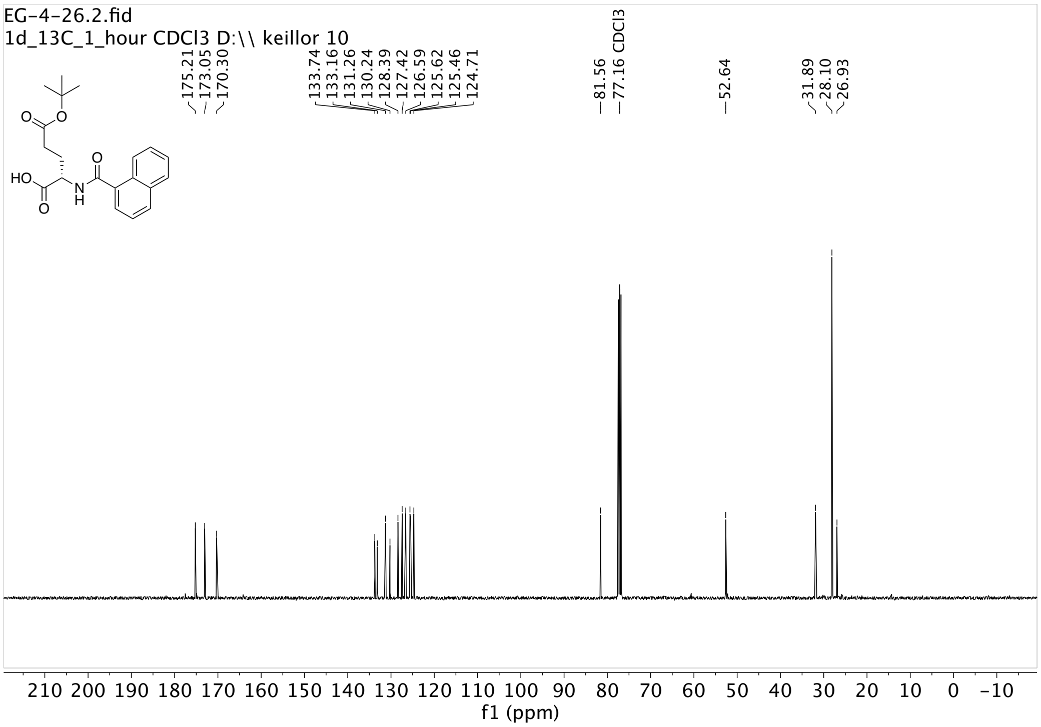

EG-4-26.2.fid
1d_13C_1_hour CDCl3 D:\\ keillor 10
175.21 173.05 170.30 133.74 133.16 131.26 130.24 128.39 127.42 126.59 125.62 125.46 124.71 81.56 77.16 CDCl3 52.64 31.89 28.10 26.93
210 200 190 180 170 160 150 140 130 120 110 100 90 80 70 60 50 40 30 20 10 0 -10
f1 (ppm)

## *Compound 10*

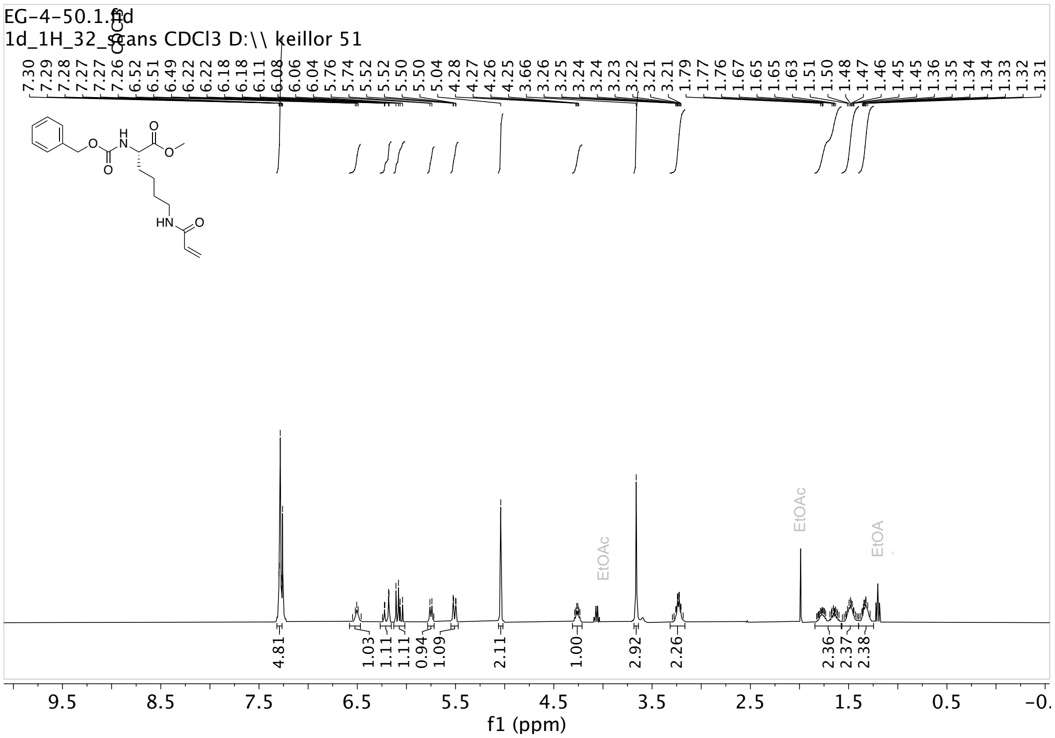

EG-4-50.1.fid
1d_1H_32_scans CDCl3 D:\\ keillor 51
CDCl3
7.30 7.29 7.28 7.27 7.27 7.26 6.52 6.51 6.49 6.22 6.22 6.18 6.18 6.11 6.08 6.06 6.04 5.76 5.74 5.52 5.52 5.52 5.50 5.50 5.04 4.28 4.27 4.26 4.25 3.66 3.26 3.25 3.24 3.24 3.23 3.22 3.21 3.21 1.79 1.77 1.76 1.67 1.65 1.65 1.63 1.51 1.50 1.48 1.47 1.46 1.45 1.45 1.36 1.35 1.34 1.34 1.33 1.32 1.31
EtOAc
EtOAc
EtOA
4.81
1.03
1.11
1.11
0.94
1.09
2.11
1.00
2.92
2.26
2.36
2.37
2.38
9.5 8.5 7.5 6.5 5.5 4.5 3.5 2.5 1.5 0.5 -0.
f1 (ppm)


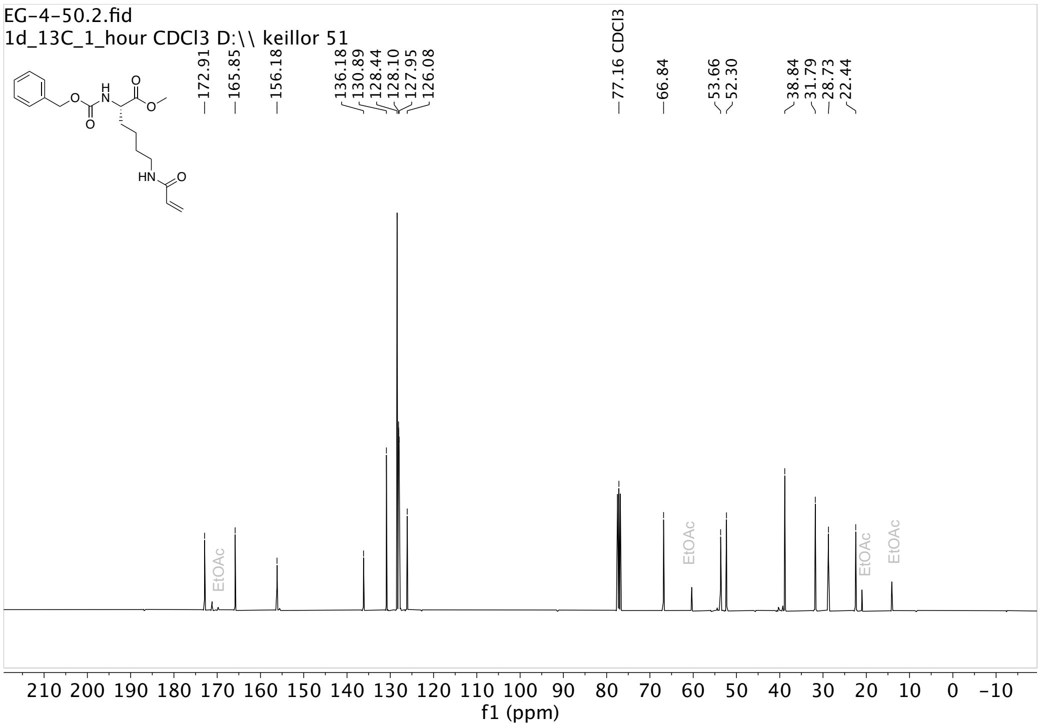

EG-4-50.2.fid
1d_13C_1_hour CDCl3 D:\\ keillor 51
172.91
165.85
156.18
136.18 130.89 128.44 128.10 127.95 126.08
77.16 CDCl3
66.84
53.66 52.30
38.84 31.79 28.73 22.44
EtOAc
EtOAc
EtOAc
EtOAc
210 200 190 180 170 160 150 140 130 120 110 100 90 80 70 60 50 40 30 20 10 0 -10
f1 (ppm)

## *Compound 11*

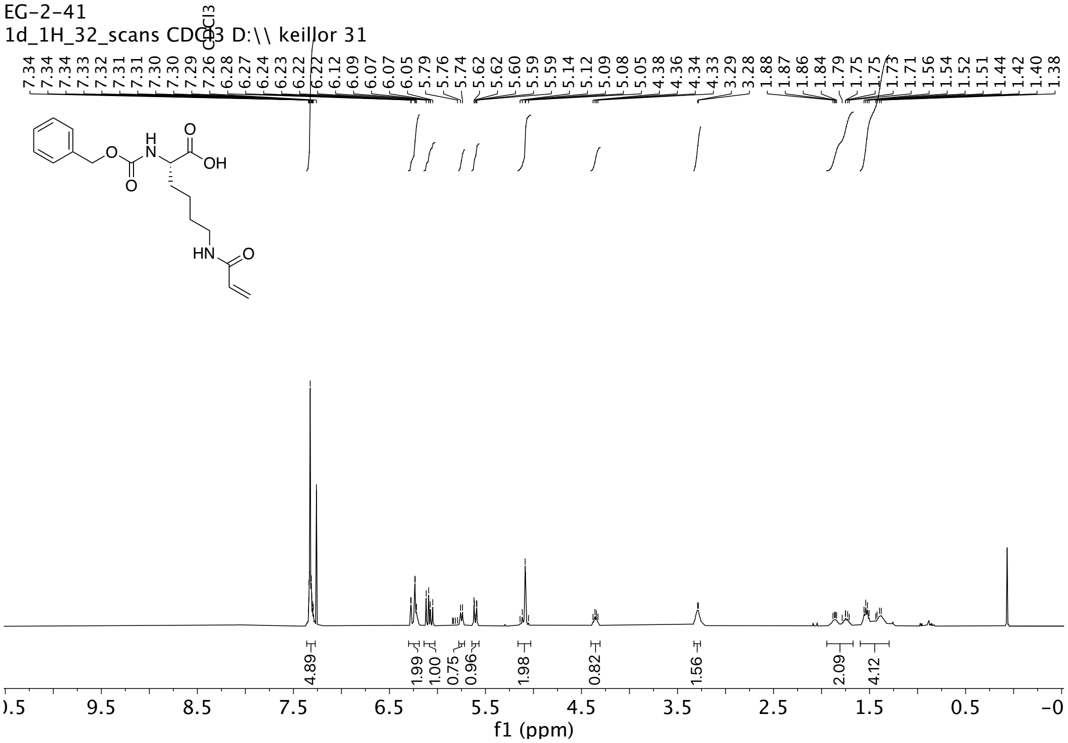
EG-2-41
1d_1H_32_scans CDCl3 D:\\ keillor 31
CDCl3
7.34 7.34 7.34 7.33 7.32 7.31 7.31 7.30 7.30 7.29 7.26 6.28 6.27 6.24 6.23 6.22 6.22 6.12 6.09 6.07 6.07 6.05 5.79 5.76 5.74 5.62 5.62 5.60 5.59 5.59 5.14 5.12 5.09 5.08 5.05 4.38 4.36 4.34 4.33 3.29 3.28 1.88 1.87 1.86 1.84 1.79 1.75 1.75 1.73 1.71 1.56 1.54 1.52 1.51 1.44 1.42 1.40 1.38
H
N
O
OH
O
O
HN
O
4.89
1.99
1.00
0.75
0.96
1.98
0.82
1.56
2.09
4.12
9.5 8.5 7.5 6.5 5.5 4.5 3.5 2.5 1.5 0.5
f1 (ppm)

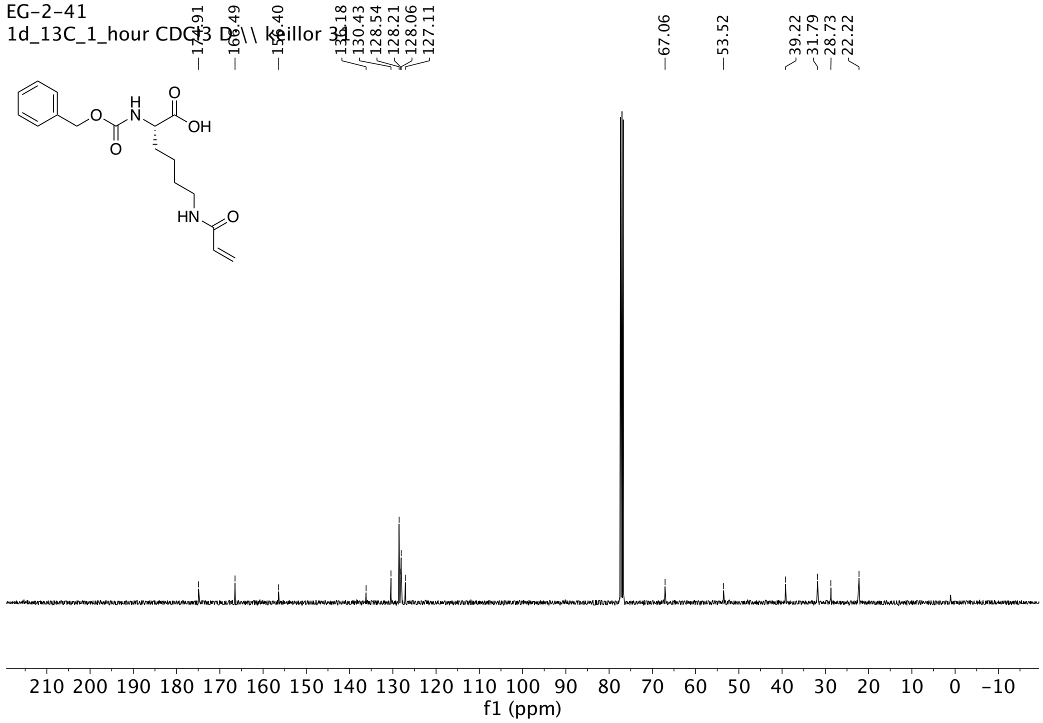
EG-2-41
1d_13C_1_hour CDCl3 D:\\ keillor 31
130.43
128.54
128.21
128.06
127.11
67.06
53.52
39.22
31.79
28.73
22.22
H
N
O
OH
O
O
HN
O
210 200 190 180 170 160 150 140 130 120 110 100 90 80 70 60 50 40 30 20 10 0 -10
f1 (ppm)

*Compound 13*

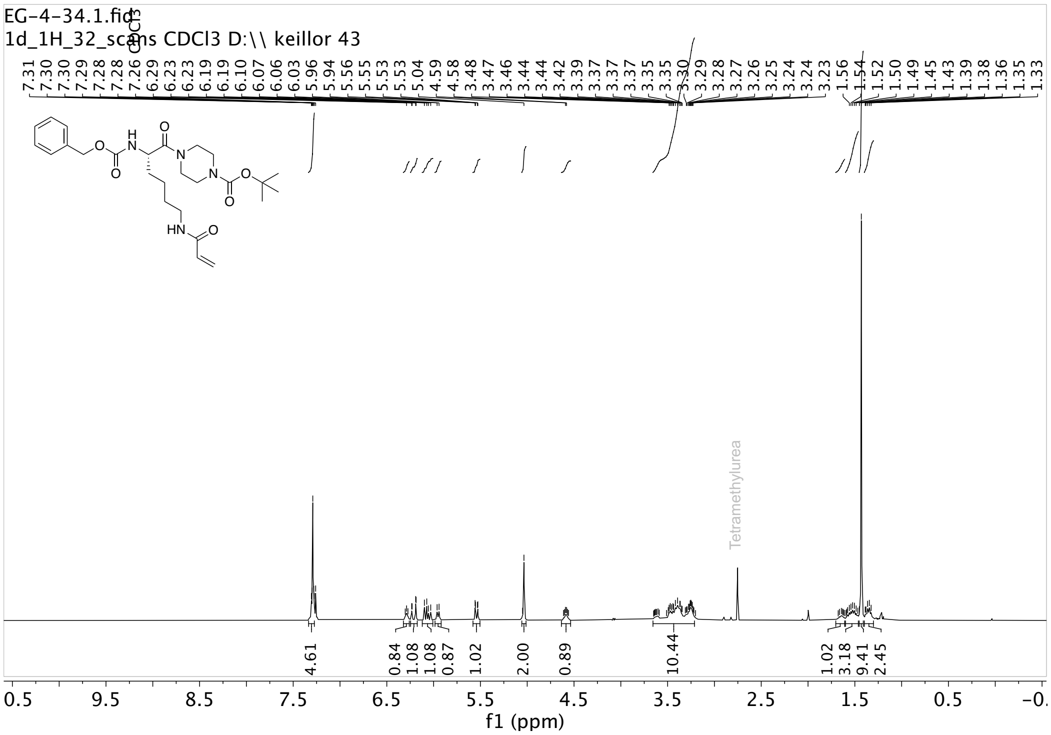
EG-4-34.1.fid
1d_1H_32_scans CDCl3 D:\\ keillor 43
Tetramethylurea
f1 (ppm)

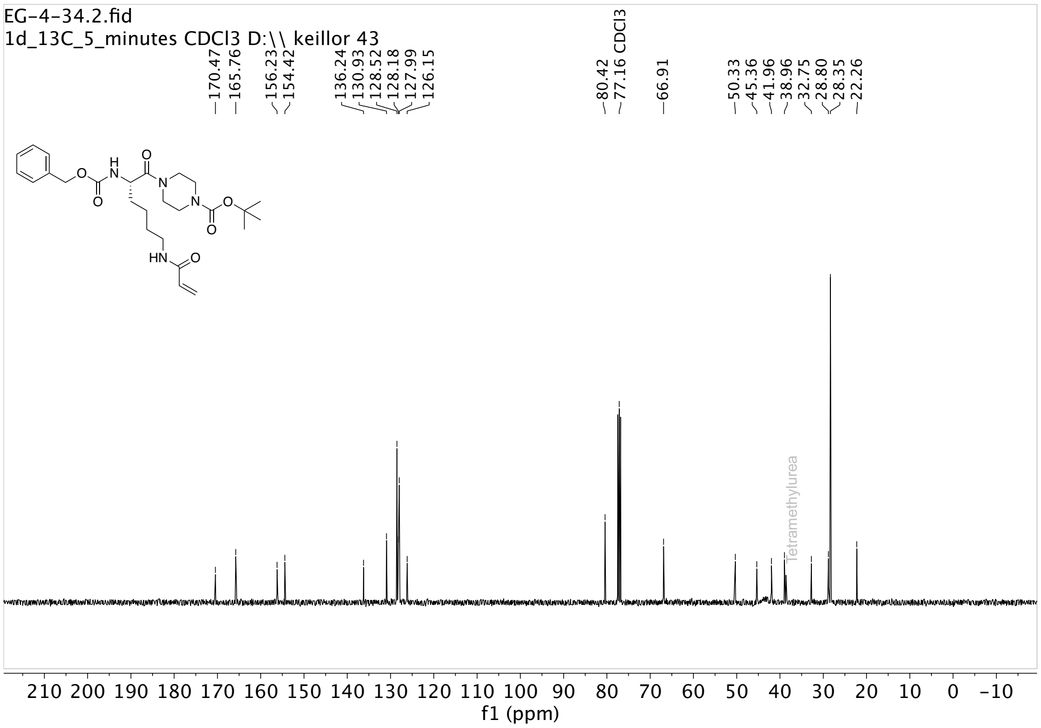
EG-4-34.2.fid
1d_13C_5_minutes CDCl3 D:\\ keillor 43
170.47
165.76
156.23
154.42
136.24
130.93
128.52
128.18
127.99
126.15
80.42
77.16 CDCl3
66.91
50.33
45.36
41.96
38.96
32.75
28.80
28.35
22.26
Tetramethylurea
f1 (ppm)

*Compound 15*

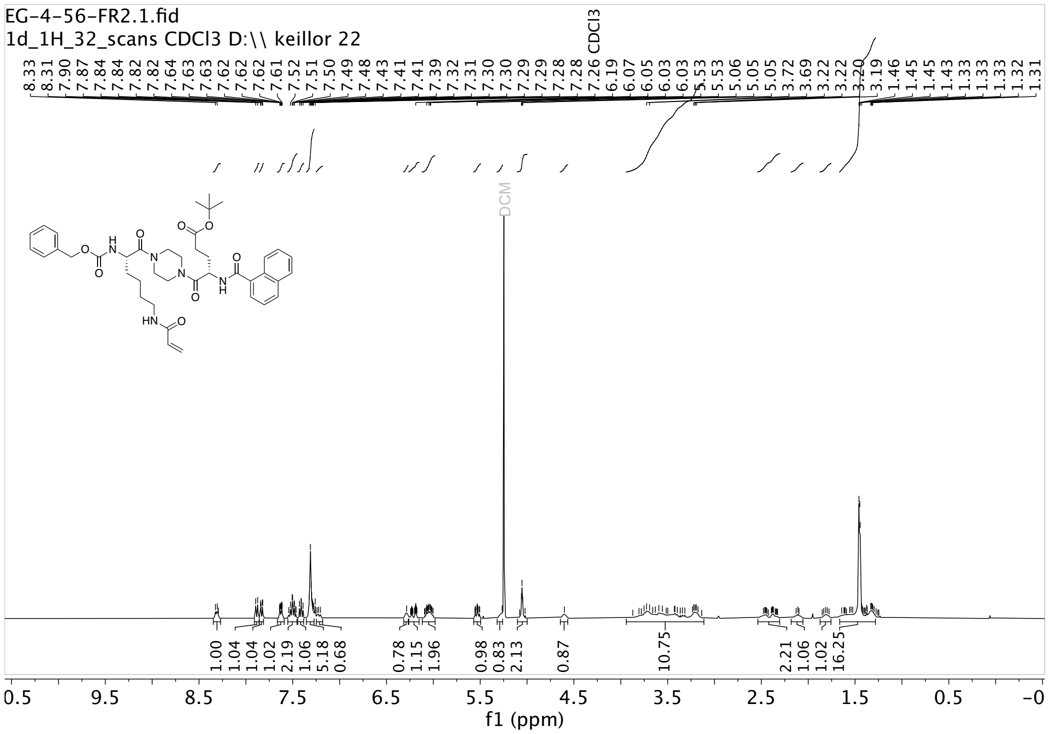
EG-4-56-FR2.1.fid
1d_1H_32_scans CDCl3 D:\\ keillor 22
DCM
f1 (ppm)

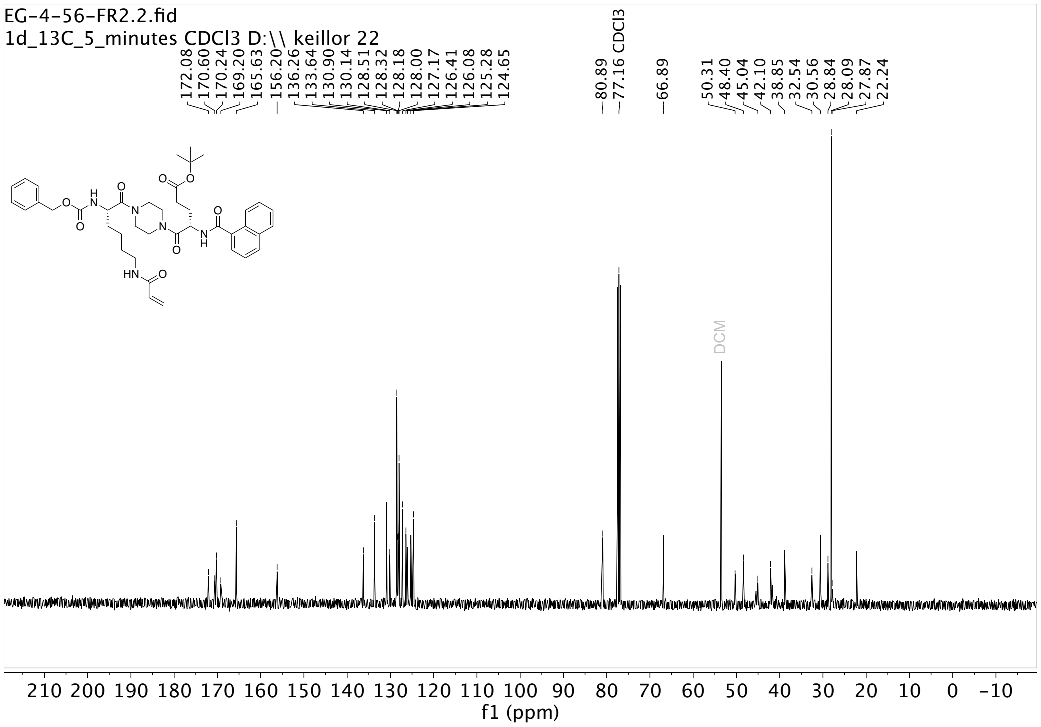
EG-4-56-FR2.2.fid
1d_13C_5_minutes CDCl3 D:\\ keillor 22
DCM
f1 (ppm)

## *Compound 16*

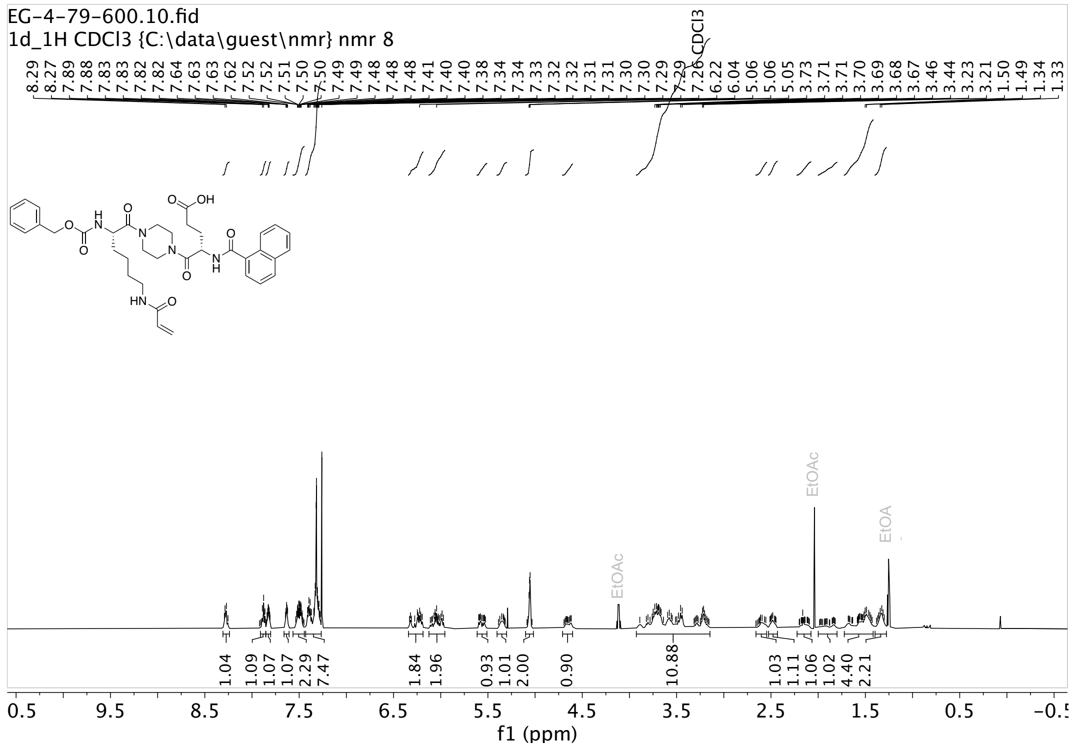

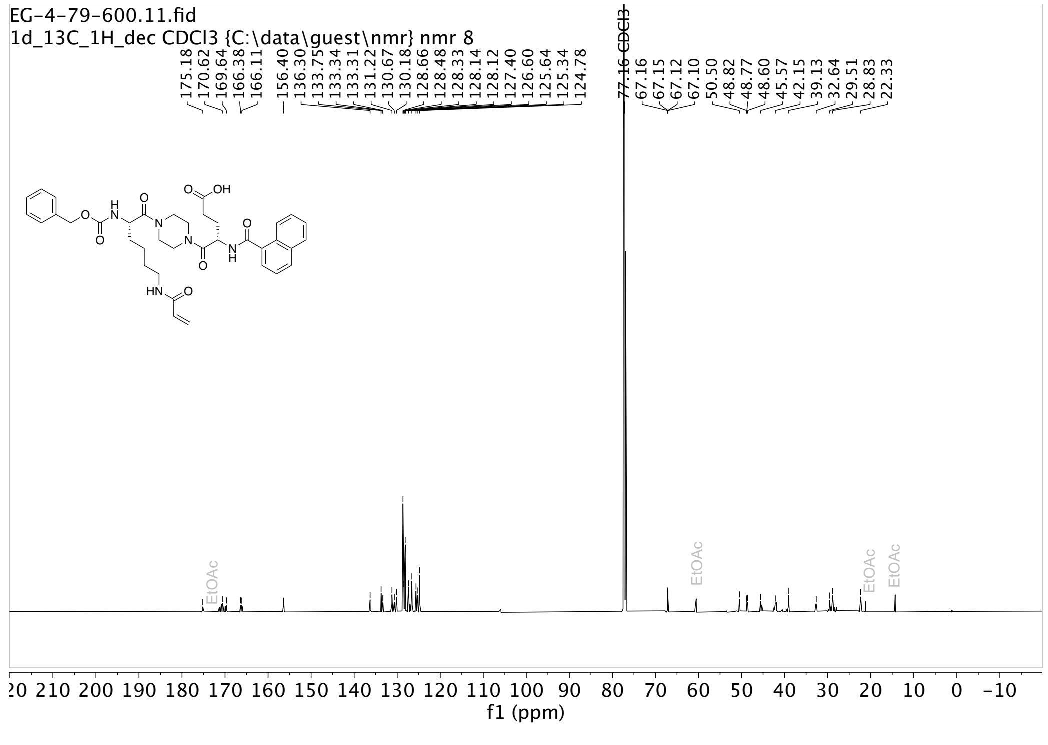

# *Compound 25*

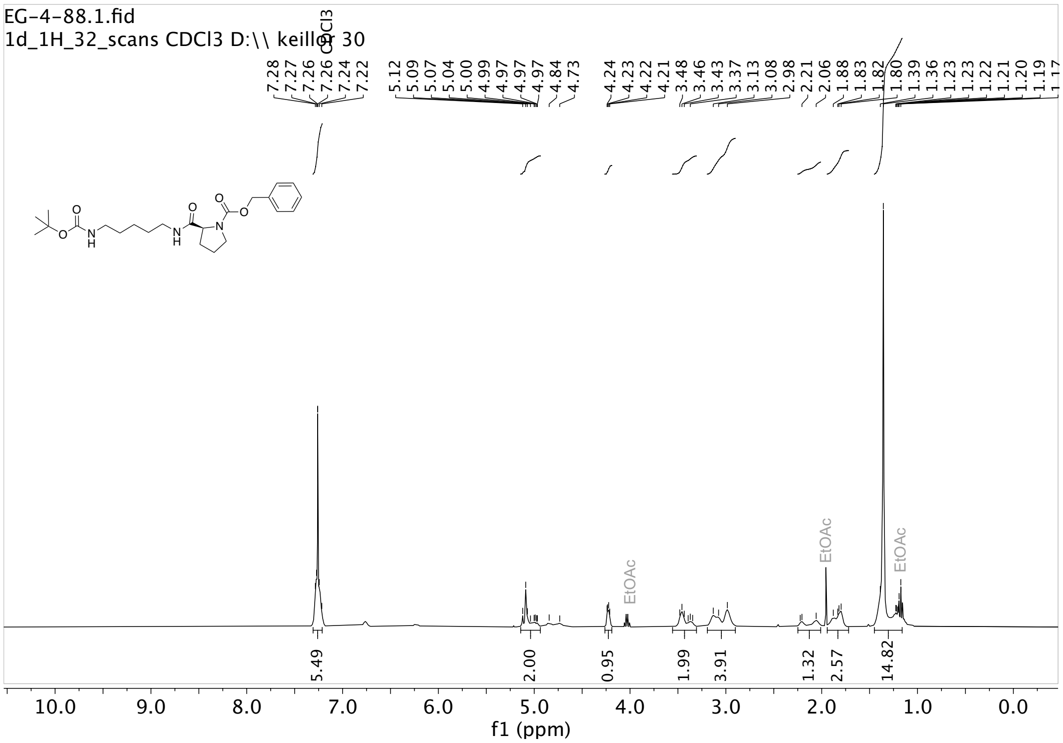

EG-4-88.1.fid
1d_1H_32_scans CDCl3 D:\\ keillor 30
7.28
7.27
7.26
7.26 CDCl3
7.24
7.22
5.12
5.09
5.07
5.04
5.00
4.99
4.97
4.97
4.97
4.84
4.73
4.24
4.23
4.22
4.21
3.48
3.46
3.43
3.37
3.13
3.08
2.98
2.21
2.06
1.88
1.83
1.82
1.80
1.39
1.36
1.23
1.23
1.22
1.21
1.20
1.19
1.17
EtOAc
EtOAc
EtOAc
5.49
2.00
0.95
1.99
3.91
1.32
2.57
14.82
10.0
9.0
8.0
7.0
6.0
5.0
4.0
3.0
2.0
1.0
0.0
f1 (ppm)


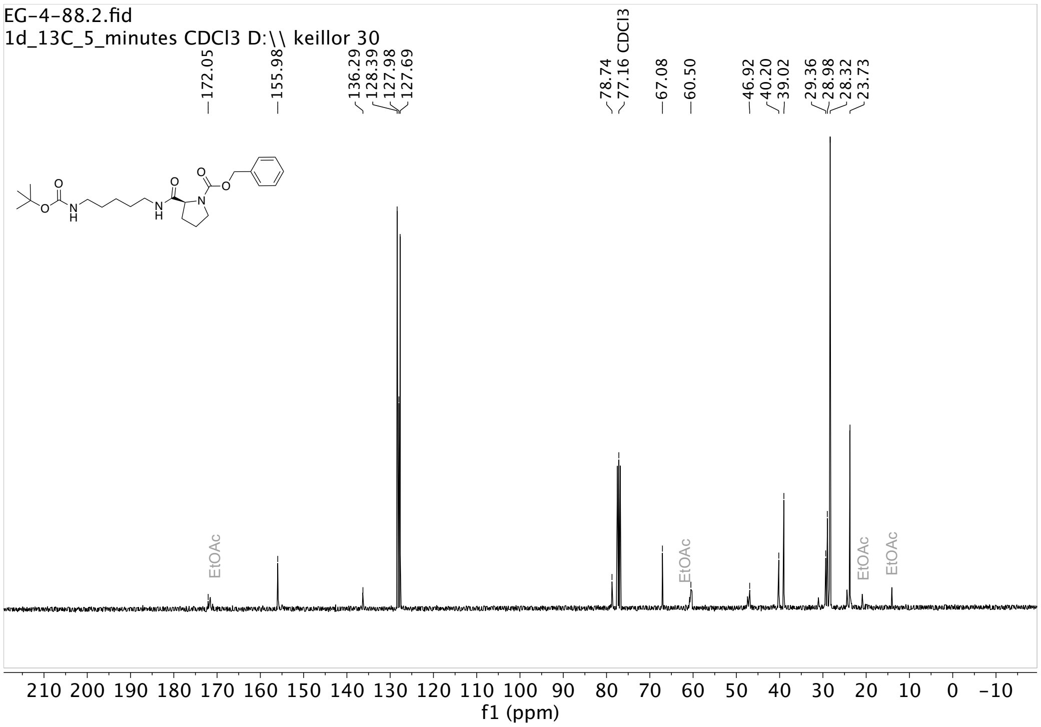

EG-4-88.2.fid
1d_13C_5_minutes CDCl3 D:\\ keillor 30
172.05
155.98
136.29
128.39
127.98
127.69
78.74
77.16 CDCl3
67.08
60.50
46.92
40.20
39.02
29.36
28.98
28.32
23.73
EtOAc
EtOAc
EtOAc
EtOAc
210 200 190 180 170 160 150 140 130 120 110 100 90 80 70 60 50 40 30 20 10 0 −10
f1 (ppm)

## *Compound 27*

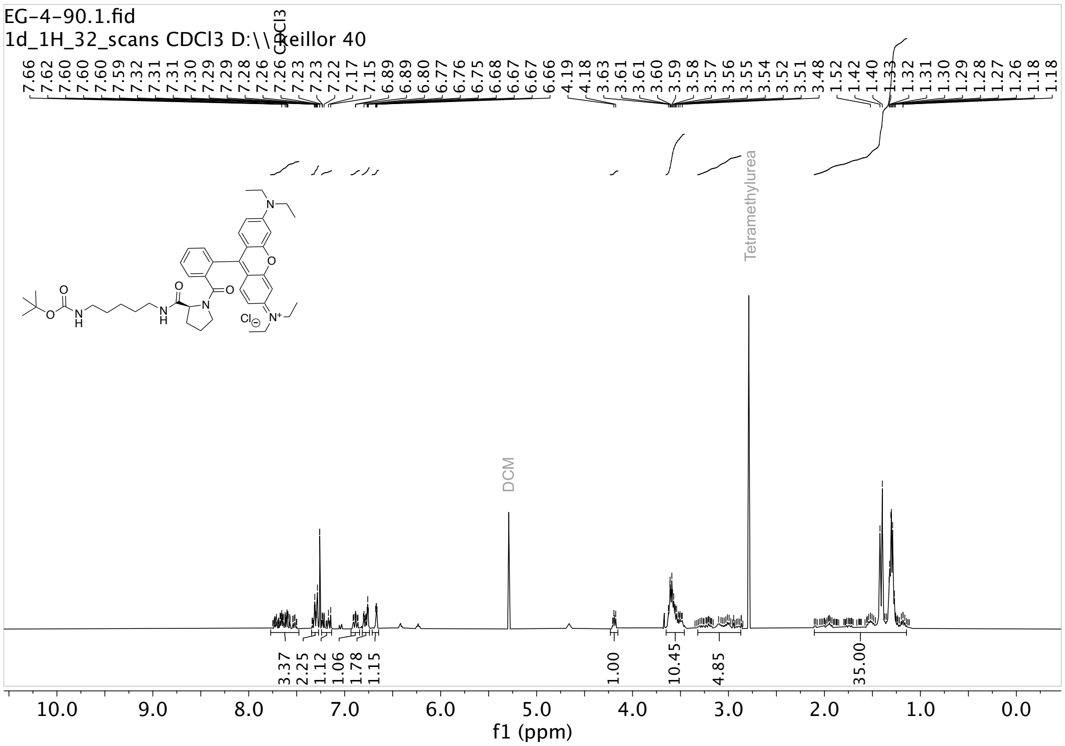
EG-4-90.1.fid
1d_1H_32_scans CDCl3 D:\\ keillor 40
7.66 7.62 7.60 7.60 7.60 7.59 7.32 7.31 7.31 7.30 7.29 7.29 7.28 7.26 7.26 CDCl3 7.23 7.23 7.22 7.17 7.15 6.89 6.89 6.80 6.77 6.76 6.75 6.68 6.67 6.67 6.66 4.19 4.18 3.63 3.61 3.61 3.60 3.59 3.58 3.57 3.56 3.55 3.54 3.52 3.51 3.48 1.52 1.42 1.40 1.33 1.32 1.31 1.30 1.29 1.28 1.27 1.26 1.18 1.18
Tetramethylurea
DCM
3.37 2.25 1.12 1.06 1.78 1.15 1.00 10.45 4.85 35.00
10.0 9.0 8.0 7.0 6.0 5.0 4.0 3.0 2.0 1.0 0.0
f1 (ppm)

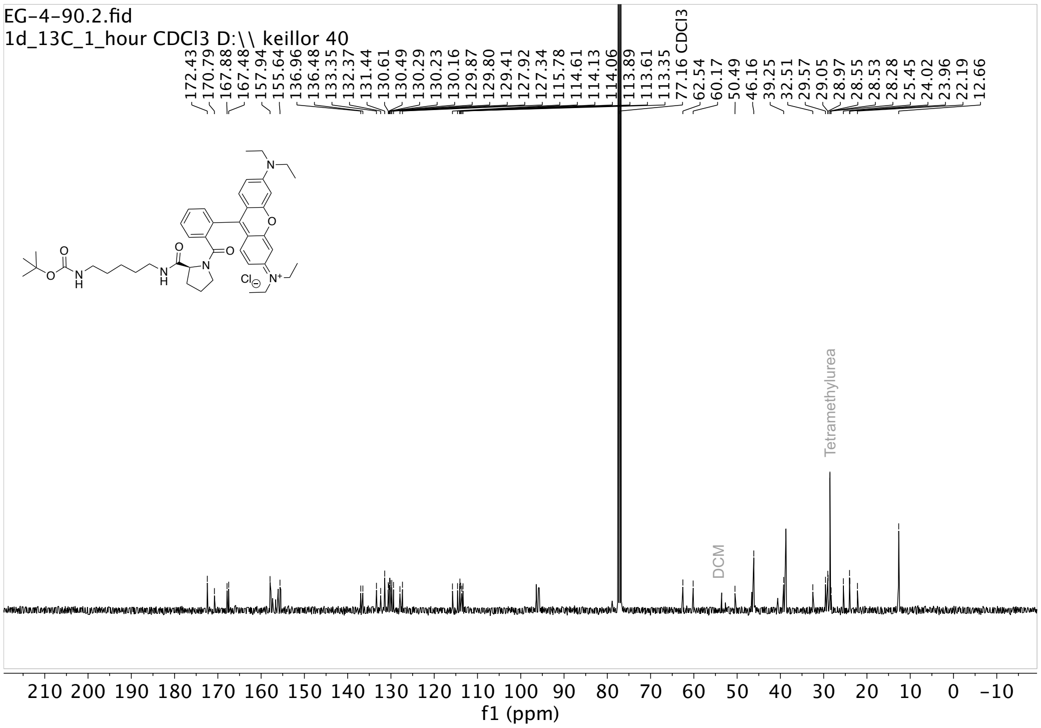
EG-4-90.2.fid
1d_13C_1_hour CDCl3 D:\\ keillor 40
172.43 170.79 167.88 167.48 157.94 155.64 136.96 136.48 133.35 132.37 131.44 130.61 130.49 130.29 130.23 130.16 129.87 129.80 129.41 127.92 127.34 115.78 114.61 114.13 114.06 113.89 113.61 113.35 77.16 CDCl3 62.54 60.17 50.49 46.16 39.25 32.51 29.57 29.05 28.97 28.55 28.53 28.28 25.45 24.02 23.96 22.19 12.66
Tetramethylurea
DCM
210 200 190 180 170 160 150 140 130 120 110 100 90 80 70 60 50 40 30 20 10 0 -10
f1 (ppm)

## *Compound 31*

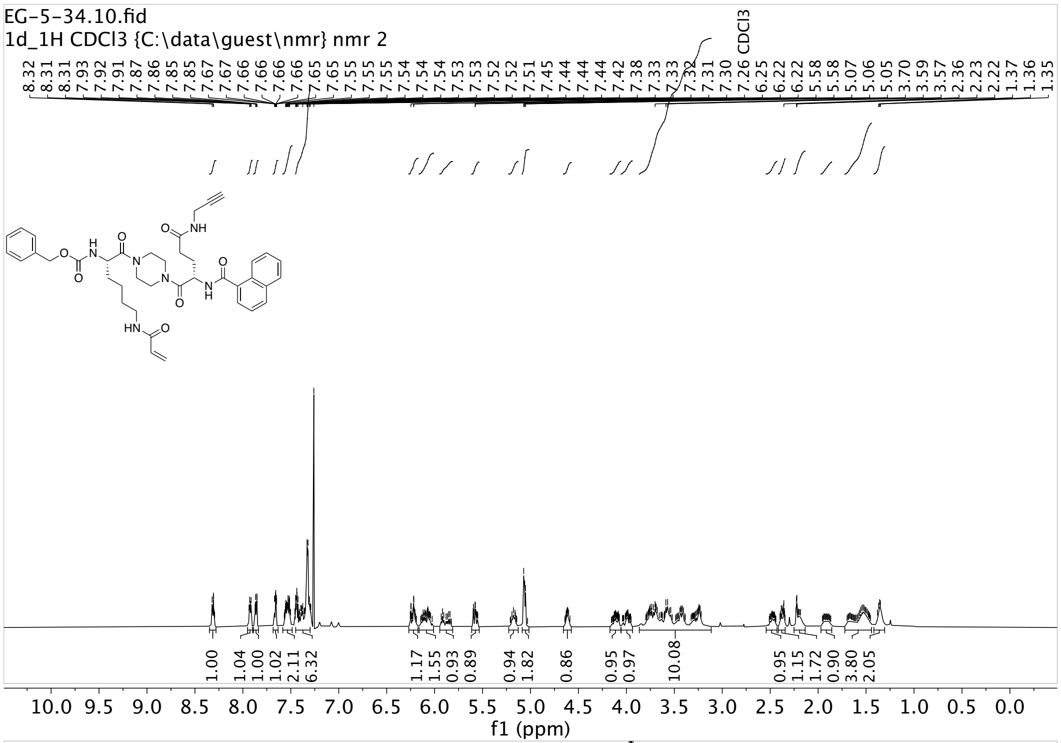
EG-5-34.10.fid
1d_1H CDCl3 {C:\data\guest\nmr} nmr 2
f1 (ppm)

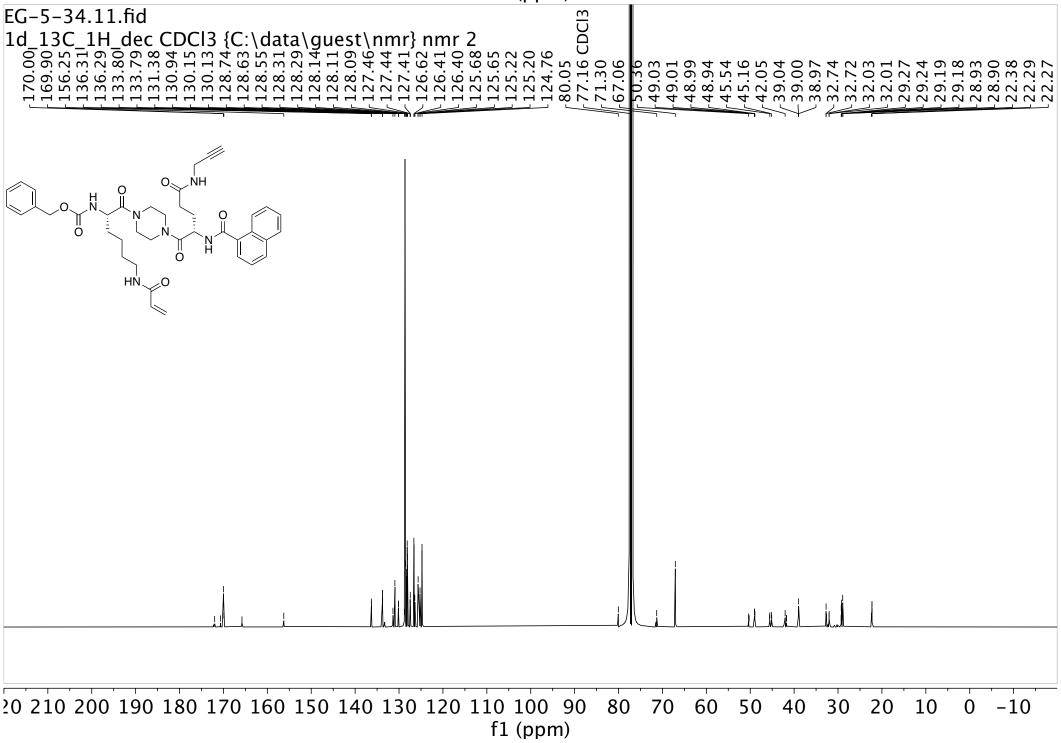
EG-5-34.11.fid
1d_13C_1H_dec CDCl3 {C:\data\guest\nmr} nmr 2
f1 (ppm)

## *Compound 32*

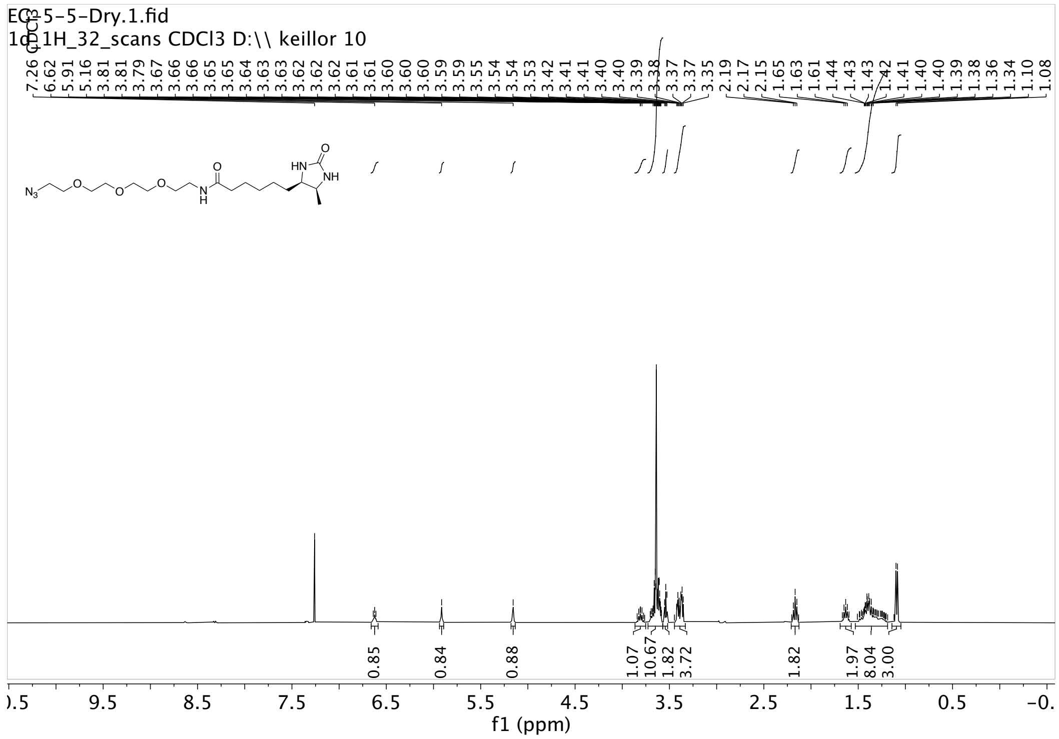
EG-5-5-Dry.1.fid
1d_1H_32_scans CDCl3 D:\\ keillor 10
CDCl3
7.26
6.62
5.91
5.16
3.81
3.81
3.79
3.67
3.66
3.66
3.65
3.65
3.64
3.63
3.63
3.62
3.62
3.62
3.61
3.61
3.60
3.60
3.59
3.59
3.55
3.54
3.54
3.53
3.42
3.41
3.41
3.40
3.40
3.39
3.38
3.37
3.37
3.35
2.19
2.17
2.15
1.65
1.63
1.61
1.44
1.43
1.43
1.42
1.41
1.40
1.40
1.39
1.38
1.36
1.34
1.10
1.08
N3
O
O
O
N
H
O
HN
NH
O
0.85
0.84
0.88
1.07
10.67
1.82
3.72
1.82
1.97
8.04
3.00
9.5
8.5
7.5
6.5
5.5
4.5
3.5
2.5
1.5
0.5
f1 (ppm)

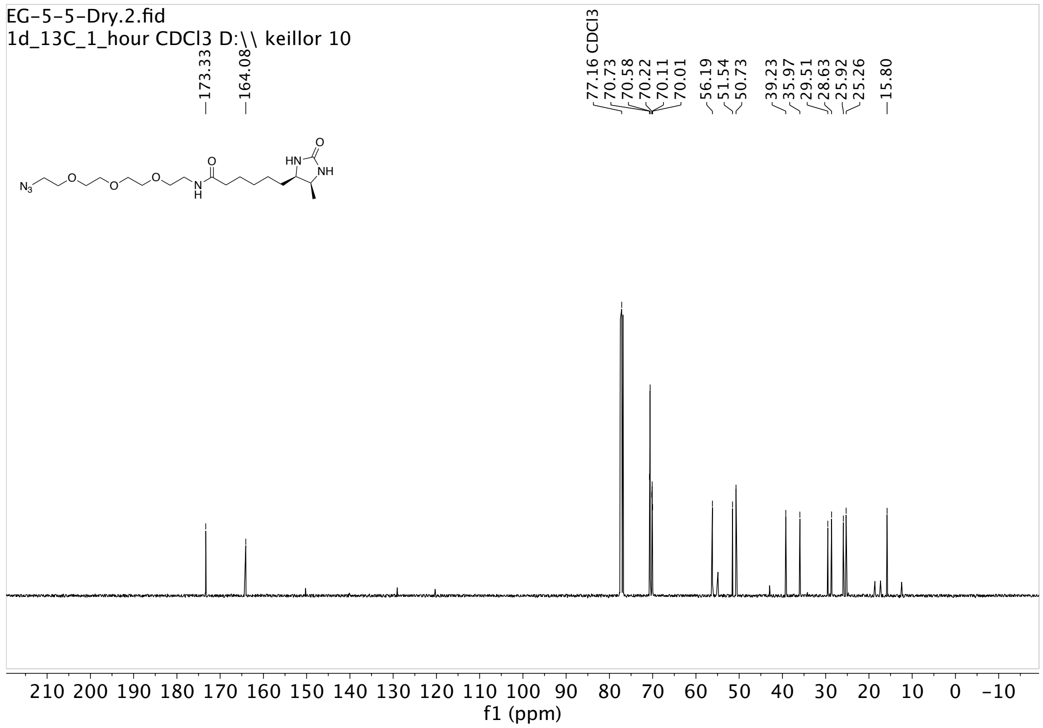
EG-5-5-Dry.2.fid
1d_13C_1_hour CDCl3 D:\\ keillor 10
173.33
164.08
77.16 CDCl3
70.73
70.58
70.22
70.11
70.01
56.19
51.54
50.73
39.23
35.97
29.51
28.63
25.92
25.26
15.80
N3
O
O
O
N
H
O
HN
NH
O
210 200 190 180 170 160 150 140 130 120 110 100 90 80 70 60 50 40 30 20 10 0 -10
f1 (ppm)

# HPLC traces of final compounds

## *Compound 17:*

### <Sample Information>

| | | | |
|---|---|---|---|
| Sample Name | : EG-4-29 | | |
| Sample ID | : EG-4-29 | | |
| Data Filename | : EG-4-29 | | |
| Method Filename | : 5-95% Method.lcm | | |
| Batch Filename | : EG-4-29 | | |
| Vial # | : 1-19 | Sample Type | : Unknown |
| Injection Volume | : 30 uL | | |
| Date Acquired | : 12/15/2021 11:59:11 AM | Acquired by | : Eric Gates |
| Date Processed | : 12/15/2021 12:14:15 PM | Processed by | : Eric Gates |

### <Chromatogram>

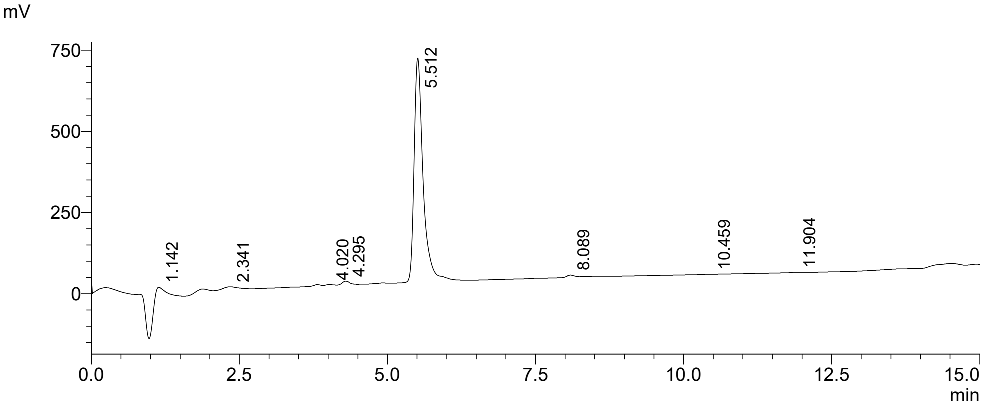


Peak Table

Detector A Channel 1 220nm

| Peak# | Ret. Time | Area% |
|---|---|---|
| 1 | 1.142 | 0.805 |
| 2 | 2.341 | 1.503 |
| 3 | 4.020 | 0.498 |
| 4 | 4.295 | 1.070 |
| 5 | 5.512 | 95.488 |
| 6 | 8.089 | 0.572 |
| 7 | 10.459 | 0.023 |
| 8 | 11.904 | 0.041 |
| Total | | 100.000 |

## *Compound 18 (NCEG2):*

### <Sample Information>

| | | | |
|---|---|---|---|
| Sample Name | : EG-5-11-Purity | | |
| Sample ID | : EG-5-11-Purity | | |
| Data Filename | : EG-5-11-Purity.lcd | | |
| Method Filename | : 5-95% Method.lcm | | |
| Batch Filename | : | | |
| Vial # | : 0-2 | Sample Type | : Unknown |
| Injection Volume | : 10 uL | | |
| Date Acquired | : 9/1/2022 11:01:55 AM | Acquired by | : Boddy lab |
| Date Processed | : 9/1/2022 11:21:26 AM | Processed by | : Boddy lab |

---

### <Chromatogram>

MS Spectrum

R.Time:5.767(Scan#:347)
MassPeaks:1646
Segment 1 - Event 1

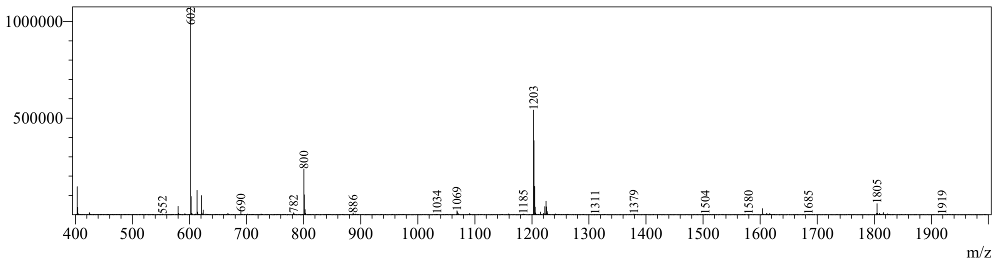


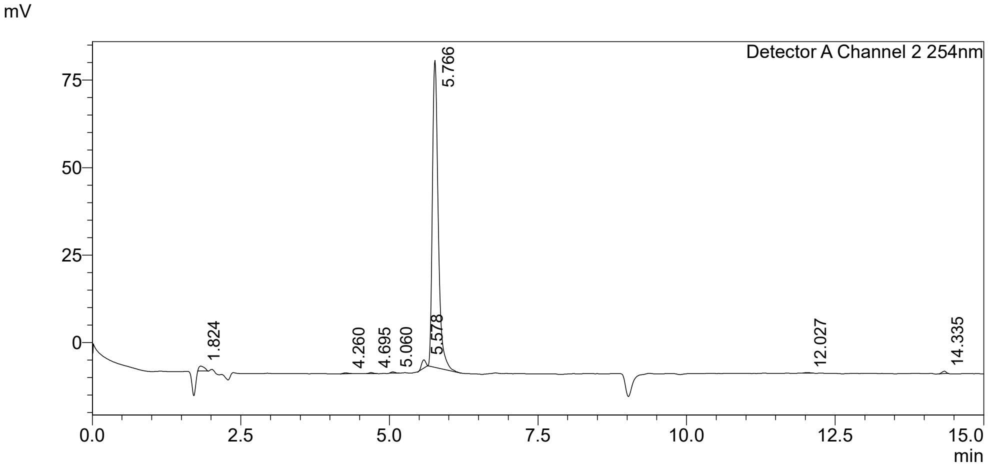


Peak Table
Detector A Channel 2 254nm

| Peak# | Ret. Time | Area% |
|---|---|---|
| 1 | 1.824 | 1.438 |
| 2 | 4.260 | 0.222 |
| 3 | 4.695 | 0.219 |
| 4 | 5.060 | 0.272 |
| 5 | 5.578 | 1.827 |
| 6 | 5.766 | 95.387 |
| 7 | 12.027 | 0.144 |
| 8 | 14.335 | 0.492 |
| Total | | 100.000 |

*Compound 22:*

## <Sample Information>

| | | | |
|---|---|---|---|
| Sample Name | : EG-4-65 | | |
| Sample ID | : EG-4-65 | | |
| Data Filename | : EG-4-65_002.lcd | | |
| Method Filename | : 5-95% Method.lcm | | |
| Batch Filename | : EG-4-64-65-87-Purity.lcb | | |
| Vial # | : 0-5 | Sample Type | : Unknown |
| Injection Volume | : 10 uL | | |
| Date Acquired | : 4/29/2022 10:20:06 AM | Acquired by | : Boddy lab |
| Date Processed | : 4/29/2022 10:35:07 AM | Processed by | : Boddy lab |

## <Chromatogram>

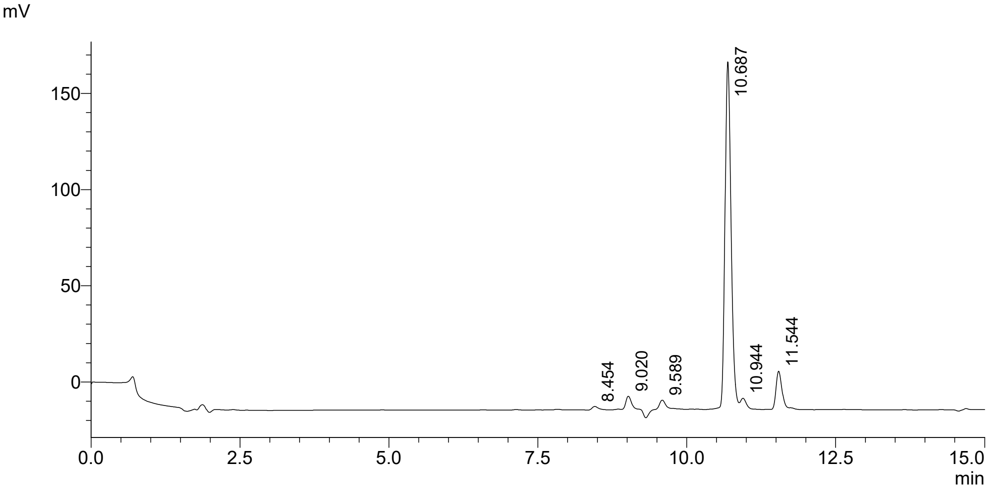


Peak Table
Detector A Channel 2 254nm

| Ret. Time | Area% |
|---|---|
| 8.454 | 0.580 |
| 9.020 | 2.489 |
| 9.589 | 1.625 |
| 10.687 | 85.300 |
| 10.944 | 1.605 |
| 11.544 | 8.401 |
| | 100.000 |

MS Spectrum

R.Time:10.683(Scan#:642)
MassPeaks:1273
Segment 1 - Event 1

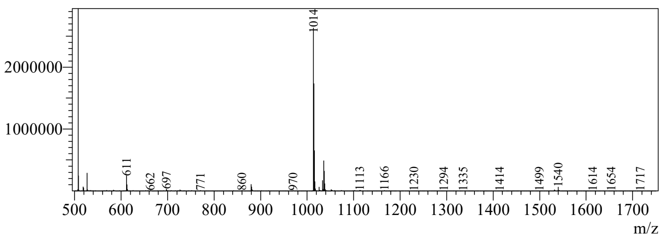

## *Compound 23:*

### <Sample Information>

| | | | |
|---|---|---|---|
| Sample Name | : EG-4-64 | | |
| Sample ID | : EG-4-64 | | |
| Data Filename | : EG-4-64_001.lcd | | |
| Method Filename | : 5-95% Method.lcm | | |
| Batch Filename | : EG-4-64-65-87-Purity.lcb | | |
| Vial # | : 0-4 | Sample Type | : Unknown |
| Injection Volume | : 10 uL | | |
| Date Acquired | : 4/29/2022 10:04:34 AM | Acquired by | : Eric Gates |
| Date Processed | : 4/29/2022 10:19:36 AM | Processed by | : Eric Gates |

### <Chromatogram>

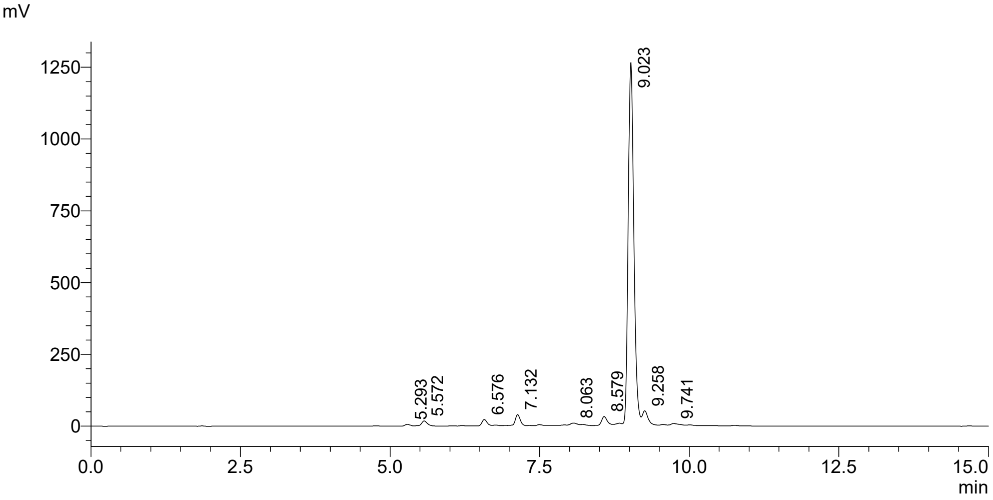


Peak Table
Detector A Channel 2 254nm

| Ret. Time | Area% |
|---|---|
| 5.293 | 0.255 |
| 5.572 | 0.943 |
| 6.576 | 1.268 |
| 7.132 | 2.143 |
| 8.063 | 0.425 |
| 8.579 | 1.827 |
| 9.023 | 89.915 |
| 9.258 | 3.059 |
| 9.741 | 0.166 |
| | 100.000 |

MS Spectrum

R.Time:9.017(Scan#:542)
MassPeaks:1293
Segment 1 - Event 1

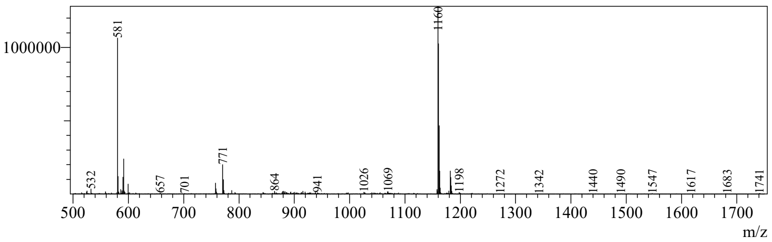

## *Compound 29 (NCEG-RHB):*

### <Sample Information>

| | | | |
|---|---|---|---|
| Sample Name | : EG-5-2-Purity | | |
| Sample ID | : EG-5-2-Purity | | |
| Data Filename | : EG-5-2-Purity_002.lcd | | |
| Method Filename | : 5-95% Method.lcm | | |
| Batch Filename | : EG-4-77-EG-5-2-6-Purity.lcb | | |
| Vial # | : 0-3 | Sample Type | : Unknown |
| Injection Volume | : 10 uL | | |
| Date Acquired | : 9/19/2022 11:35:22 AM | Acquired by | : Boddy lab |
| Date Processed | : 9/19/2022 11:50:24 AM | Processed by | : Boddy lab |

### <Chromatogram>

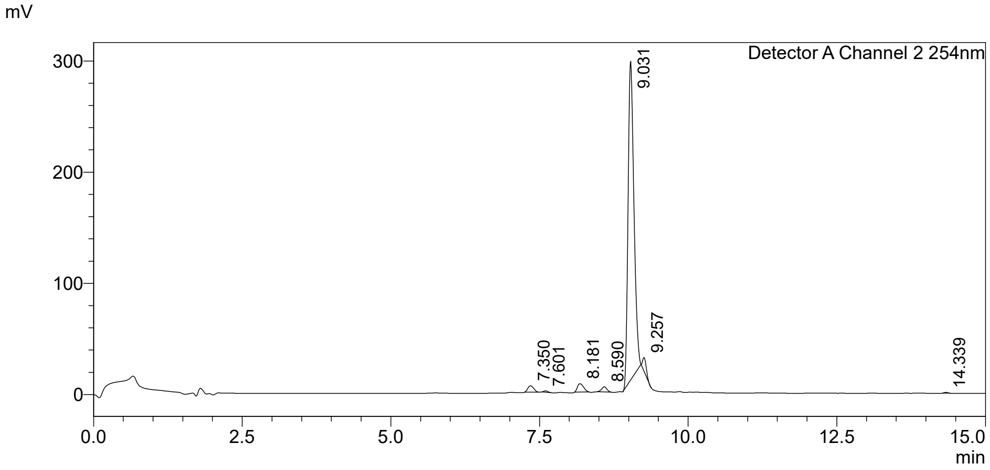


Peak Table
Detector A Channel 2 254nm

| Peak# | Ret. Time | Area% |
|---|---|---|
| 1 | 7.350 | 1.839 |
| 2 | 7.601 | 0.242 |
| 3 | 8.181 | 2.447 |
| 4 | 8.590 | 1.270 |
| 5 | 9.031 | 91.881 |
| 6 | 9.257 | 2.184 |
| 7 | 14.339 | 0.137 |
| Total | | 100.000 |

## *Compound 31:*

### <Sample Information>

| | | | |
|---|---|---|---|
| Sample Name | : EG-4-77-Purity | | |
| Sample ID | : EG-4-77-Purity | | |
| Data Filename | : EG-4-77-Purity_001.lcd | | |
| Method Filename | : 5-95% Method.lcm | | |
| Batch Filename | : EG-4-77-EG-5-2-6-Purity.lcb | | |
| Vial # | : 0-2 | Sample Type | : Unknown |
| Injection Volume | : 10 uL | | |
| Date Acquired | : 9/19/2022 11:19:51 AM | Acquired by | : Boddy lab |
| Date Processed | : 9/19/2022 11:49:31 AM | Processed by | : Boddy lab |

### <Chromatogram>

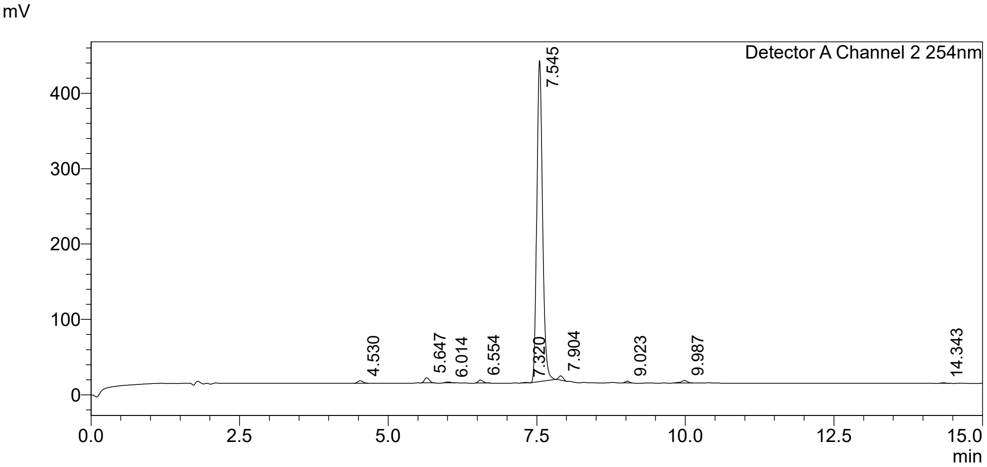


Peak Table
Detector A Channel 2 254nm

| Peak# | Ret. Time | Area% |
|---|---|---|
| 1 | 4.530 | 0.673 |
| 2 | 5.647 | 1.273 |
| 3 | 6.014 | 0.224 |
| 4 | 6.554 | 0.787 |
| 5 | 7.320 | 0.115 |
| 6 | 7.545 | 94.734 |
| 7 | 7.904 | 0.937 |
| 8 | 9.023 | 0.317 |
| 9 | 9.987 | 0.825 |
| 10 | 14.343 | 0.116 |
| Total | | 100.000 |

## *Compound 33:*

### <Sample Information>

| | | | |
|---|---|---|---|
| Sample Name | : EG-5-6-Purity | | |
| Sample ID | : EG-5-6-Purity | | |
| Data Filename | : EG-5-6-Purity_003.lcd | | |
| Method Filename | : 5-95% Method.lcm | | |
| Batch Filename | : EG-4-77-EG-5-2-6-Purity.lcb | | |
| Vial # | : 0-4 | Sample Type | : Unknown |
| Injection Volume | : 10 uL | | |
| Date Acquired | : 9/19/2022 11:50:54 AM | Acquired by | : Boddy lab |
| Date Processed | : 9/19/2022 12:05:56 PM | Processed by | : Boddy lab |

### <Chromatogram>

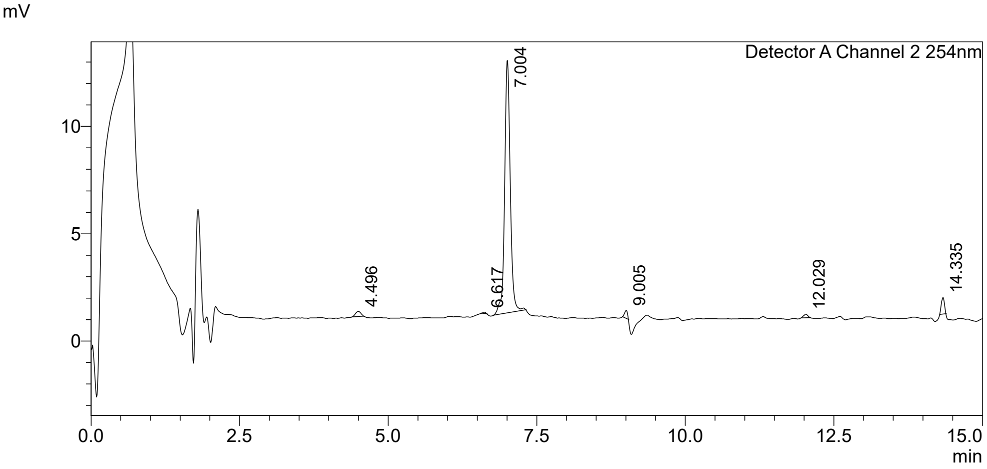


Peak Table
Detector A Channel 2 254nm

| Peak# | Ret. Time | Area% |
|---|---|---|
| 1 | 4.496 | 1.831 |
| 2 | 6.617 | 0.238 |
| 3 | 7.004 | 92.413 |
| 4 | 9.005 | 1.459 |
| 5 | 12.029 | 0.916 |
| 6 | 14.335 | 3.142 |
| Total | | 100.000 |

**References**

53. Hauser, S.; Sommerfeld, P.; Wodtke, J.; Hauser, C.; Schlitterlau, P.; Pietzsch, J.; Löser, R.; Pietsch, M.; Wodtke, R. Application of a Fluorescence Anisotropy-Based Assay to Quantify Transglutaminase 2 Activity in Cell Lysates. *Int J Mol Sci* **2022**, *23*, 4475, doi:10.3390/ijms23094475.
55. Atherton, E.; Logan, C.J.; Sheppard, R.C. Peptide Synthesis. Part 2. Procedures for Solid-Phase Synthesis Using Nα-Fluorenylmethoxycarbonylamino-Acids on Polyamide Supports. Synthesis of Substance P and of Acyl Carrier Protein 65-74 Decapeptide. *J Chem Soc Perkin Trans 1* **1981**, 538–546, doi:10.1039/P19810000538.
56. Gude, M.; Ryf, J.; White, P.D. An Accurate Method for the Quantitation of Fmoc-Derivatized Solid Phase Supports. *Letters in Peptide Science* **2002**, *9*, 203–206, doi:10.1007/BF02538384.
